# Supplementary material for: Reverse metabolomics for the discovery of chemical structures from humans
Source: Nature. Author manuscript; Available in PMC 2024 Mar 8. (PMC10849969; doi:10.1038/s41586-023-06906-8)
Supplement: Supplementary Information [file NIHMS1962612-supplement-Supplementary_Information.docx]

**SUPPORTING INFORMATION**

**Authors:** Emily C. Gentry^1,2^, Stephanie L. Collins^15^, Morgan Panitchpakdi^1,2^, Pedro Belda-Ferre^3,11^, Allison K. Stewart^8^, Marvic Carrillo Terrazas^19^, Hsueh-han Lu^19^, Simone Zuffa^1,2^, Tingting Yan^21^, Julian Avila-Pacheco^4^, Damian R. Plichta^4^, Allegra T. Aron^1,2^, Mingxun Wang^1,2^, Alan K. Jarmusch^1,2,14^, Fuhua Hao^10^, Mashette Syrkin-Nikolau^17^, Hera Vlamakis^4,5^, Ashwin N. Ananthakrishnan^16^, Brigid Boland^18^, Amy Hemperly^17^, Niels Vande Casteele^18^, Frank J. Gonzalez^21^, Clary B. Clish^4^, Ramnik J. Xavier,^4,5,6,7^, Hiutung Chu^19,20^, Erin S. Baker^8,9^, Andrew D. Patterson^10^, Rob Knight^3,11-13^, Dionicio Siegel^1^, Pieter C. Dorrestein^1,2^#

**Affiliations:**

^1^ Skaggs School of Pharmacy and Pharmaceutical Sciences, University of California San Diego, La Jolla, San Diego, CA, USA

**^2^** Collaborative Mass Spectrometry Innovation Center, Skaggs School of Pharmacy and Pharmaceutical Sciences, University of California San Diego, La Jolla, CA, USA

^3^ Department of Pediatrics, University of California San Diego, La Jolla, CA, USA

^4^ Broad Institute of MIT and Harvard, Cambridge, Massachusetts, USA

^5^ Center for Microbiome Informatics and Therapeutics, Massachusetts Institute of Technology, Cambridge, MA 02139, USA

^6^ Center for Computational and Integrative Biology Massachusetts General Hospital and Harvard Medical School, Boston, MA 02114, USA

^7^ Department of Molecular Biology, Massachusetts General Hospital and Harvard Medical School, Boston, MA 02114, USA

^8^ Department of Chemistry, North Carolina State University, Raleigh, NC, USA

^9^ Comparative Medicine Institute, North Carolina State University, Raleigh, NC, USA

^10^ Center for Molecular Toxicology and Carcinogenesis, Department of Veterinary and Biomedical Sciences, The Pennsylvania State University, University Park, PA, USA

^11^ Department of Computer Science, Jacobs School of Engineering, University of California, San Diego, California, USA

^12^ Center for Microbiome Innovation, Jacobs School of Engineering, University of California, San Diego, California, USA

^13^ Department of Bioengineering, University of California, San Diego, California, USA

^14^ Immunity, Inflammation, and Disease Laboratory, Division of Intramural Research, National Institute of Environmental Health Sciences, National Institutes of Health, Research Triangle Park, NC 27709, USA

^15^ Department of Biochemistry and Molecular Biology, The Pennsylvania State University, University Park, PA, USA

^16^ ​​Division of Gastroenterology, Massachusetts General Hospital, Boston, MA, USA

^17^ Division of Gastroenterology, Department of Pediatrics, Rady Children's Hospital University of California, San Diego, La Jolla, California

^18^ Division of Gastroenterology, University of California, San Diego, La Jolla, California, USA.

^19^ Department of Pathology, University of California, San Diego, La Jolla, California, USA

^20^ CU-UCSD, Center for Mucosal Immunology, Allergy and Vaccine Development, University of California, San Diego, La Jolla, California, USA

^21^ Laboratory of Metabolism, Center for Cancer Research, National Cancer Institute, National Institutes of Health, Bethesda, MD 20892, United States of America.

**TABLE OF CONTENTS**

**S1-S5: Supplementary Methods + characterization data of pure compounds.**

**S5-S8: Supplementary Tables on spectral match count.**

**S9-S22: Supplementary ^1^H and ^13^C NMR Data of pure synthetic standards.**

**S23: Gating strategy for flow cytometry**

**Supplementary Methods**

*Data pre-processing for IBD200 dataset (MSV000084908)*

MS1 feature detection and MS/MS pairing was performed using MZmine 2.37corr17.7_kai_merge2[1,2](https://www.zotero.org/google-docs/?6QGlKw). An intensity threshold of 500 and 50 were set for MS1 and MS2 detection, respectively, with centroid data. MS1 chromatogram construction was performed using the ADAP chromatogram builder, where the minimum group size was set to 3, group intensity threshold was 500, minimum highest intensity was 1500, and mass tolerance was 0.01 *m/z* or 20ppm. Chromatogram deconvolution was then performed using a local minimum search algorithm with a chromatographic threshold of 90%, a search minimum in retention time (RT) range of 0.2 min, minimum relative height of 1%, minimum absolute threshold height of 1.5E3, minimum ratio for top/edge of 1, and a peak duration of 0.01-2 min. Pairing between MS1 and MS2 was performed with a mass tolerance of 0.01 *m/z* or 20ppm and RT range of 0.3 min. Isotope peaks were grouped, then features from different samples were aligned using the same mass and RT tolerances; alignment was performed by placing a weight of 75 on *m/z* and 25 on RT. Gap filling was performed with an intensity tolerance of 25%, an m/z tolerance of 0.005 Da or 10 ppm, and a retention time tolerance of 0.2 min. A peak area feature table was exported as a .csv file and consensus MS/MS spectral data were exported in .mgf format.

*Media preparation*

Fecal culture medium (FCM) was prepared according to a protocol by McDonald *et al.*[3](https://www.zotero.org/google-docs/?Uj6JNV) autoclaving for 20 min at 121 °C. The media was brought into an anaerobic chamber and left overnight with the lid slightly ajar to remove any remaining oxygen from the media. Then, an aqueous solution of L-cysteine was pushed through a 0.2uM syringe filter into the media solution for a concentration of 0.5% L-Cysteine (w/v) to maintain anoxic solution.

*Data pre-processing for HMP culture extracts*

MS1 feature detection and MS/MS pairing was performed using MZmine 2.37corr17.7_kai_merge2[1,2](https://www.zotero.org/google-docs/?tr3wSC). An intensity threshold of 1000 and 50 were set for MS1 and MS2 detection, respectively, with centroid data. MS1 chromatogram construction was performed using the ADAP chromatogram builder, where the minimum group size was set to 3, group intensity threshold was 1000, minimum highest intensity was 3000, and mass tolerance was 0.01 *m/z* or 20ppm. Chromatogram deconvolution was then performed using a local minimum search algorithm with a chromatographic threshold of 90%, a search minimum in retention time (RT) range of 0.2 min, minimum relative height of 1%, minimum absolute threshold height of 3E3, minimum ratio for top/edge of 1, and a peak duration of 0.01-2 min. Pairing between MS1 and MS2 was performed with a mass tolerance of 0.01 *m/z* or 20ppm and RT range of 0.3 min. Isotope peaks were grouped, then features from different samples were aligned using the same mass and RT tolerances; alignment was performed by placing a weight of 75 on *m/z* and 25 on RT. A peak area feature table was exported as a .csv file and consensus MS/MS spectral data were exported in .mgf format.

*Synthesis of pure conjugated bile acids*

Materials: Organic solutions were concentrated under reduced pressure on a Büchi rotary evaporator using a water bath. Chromatographic purification of products was accomplished by flash chromatography on Silicycle F60 silica gel. All reactions were carried out in well ventilated fume hoods. Thin-layer chromatography (TLC) was performed on Silicycle 250 μm silica gel plates. Visualization of the developed chromatogram was performed by irradiation with 254 nm UV light or treatment with a solution of ceric ammonium molybdate stain followed by heating. Yields refer to purified compounds unless otherwise noted.

Instrumentation: ^1^H and ^13^C NMR spectra were recorded on a Bruker 600 (600 and 151 MHz for ^1^H and ^13^C, respectively) instrument, and are internally referenced to residual protiosolvent signals of CD_3_OD at δ 3.31 and 49.0 ppm and (CD_3_)_2_SO at δ 2.50 and 39.51 ppm. Data for ^1^H NMR are reported as follows: chemical shift (δ ppm), integration, multiplicity (s = singlet, br s = broad singlet, d = doublet, t = triplet, q = quartet, m = multiplet), and coupling constant (Hz). Data for ^13^C NMR are reported in terms of chemical shift and no special nomenclature is used for equivalent carbons.

Glutamate conjugated cholic acid (Glu-CA): NaHCO_3_ was used as the inorganic base. Product was purified using 6-18% CH_3_OH in CH_2_Cl_2_ with 1% acetic acid to obtain a 21% isolated yield as an off-white amorphous solid. ^1^H NMR (600 MHz, CD_3_OD) δ 4.37 (dd, *J* = 8.8, 5.0 Hz, 1H), 3.96 (t, *J* = 3.0 Hz, 1H), 3.80 (q, *J* = 3.0 Hz, 1H), 3.37 (tt, *J* = 11.2, 4.4 Hz, 1H), 2.38 (t, *J* = 7.7 Hz, 2H), 2.37 – 2.22 (m, 3H), 2.18 (ddd, *J* = 13.4, 9.3, 6.6 Hz, 2H), 2.05 – 1.78 (m, 6H), 1.79 – 1.70 (m, 1H), 1.69 – 1.62 (m, 1H), 1.63 – 1.50 (m, 5H), 1.49 – 1.29 (m, 6H), 1.12 (qd, *J* = 11.8, 5.7 Hz, 1H), 1.04 (d, *J* = 6.5 Hz, 3H), 0.98 (td, *J* = 14.2, 3.5 Hz, 1H), 0.92 (s, 3H), 0.72 (s, 3H); ^13^C NMR (151 MHz, (CD_3_)_2_SO) δ 174.41, 172.68, 71.17, 70.57, 66.38, 51.74, 46.31, 45.86, 41.61, 41.46, 35.40, 35.29, 34.97, 34.49, 32.55, 31.82, 31.04, 31.00, 30.47, 28.63, 27.43, 27.24, 26.30, 22.93, 22.72, 17.23, 12.45; HRMS (ESI) exact mass calculated for [M+H]+ (C_29_H_48_NO_8_) requires m/z 538.3375, found 538.3376 with a difference of 0.19 ppm.

Glutamate conjugated chenodeoxycholic acid (Glu-CDCA): NaHCO_3_ was used as the inorganic base. Product was purified using 6-12% CH_3_OH in CH_2_Cl_2_ with 1% acetic acid to obtain a 50% yield as an off-white amorphous solid. ^1^H NMR (600 MHz, CD_3_OD) δ 4.37 (t, *J* = 6.7 Hz, 1H), 3.79 (t, *J* = 3.3 Hz, 1H), 3.37 (tt, *J* = 10.5, 4.2 Hz, 1H), 2.38 (t, *J* = 7.5 Hz, 2H), 2.35 – 2.22 (m, 2H), 2.21 – 2.12 (m, 2H), 2.03 – 1.99 (m, 1H), 1.97 – 1.80 (m, 5H), 1.74 (dt, *J* = 13.3, 8.0 Hz, 1H), 1.69 – 1.58 (m, 2H), 1.54 – 1.45 (m, 5H), 1.39 – 1.27 (m, 7H), 1.23 – 1.15 (m, 2H), 1.15 – 1.06 (m, 1H), 0.99 (d, *J* = 6.1 Hz, 3H), 0.93 (s, 3H), 0.70 (s, 3H); ^13^C NMR (151 MHz, (CD_3_)_2_SO) δ 174.34, 172.57, 70.43, 66.27, 55.71, 51.68, 50.08, 42.00, 41.49, 40.82, 35.37, 35.11, 34.88, 34.81, 32.37, 32.32, 31.67, 30.87, 30.59, 27.89, 27.13, 23.25, 22.79, 20.33, 18.43, 11.73; HRMS (ESI) exact mass calculated for [M+H]+ (C_29_H_48_NO_7_) requires m/z 522.3426, found 522.3423 with a difference of 0.57 ppm.

Glutamate conjugated deoxycholic acid (Glu-DCA): NaHCO_3_ was used as the inorganic base. Product was purified using 6-12% CH_3_OH in CH_2_Cl_2_ with 1% acetic acid to obtain a 55% yield as an off-white amorphous solid. ^1^H NMR (600 MHz, CD_3_OD) δ 4.37 (dd, *J* = 8.4, 4.8 Hz, 1H), 3.96 (t, *J* = 3.1 Hz, 1H), 3.57 – 3.48 (m, 1H), 2.38 (t, *J* = 7.6 Hz, 2H), 2.35 – 2.29 (m, 1H), 2.21 – 2.12 (m, 2H), 1.96 – 1.75 (m, 8H), 1.66 – 1.57 (m, 3H), 1.55 – 1.50 (m, 2H), 1.48 – 1.23 (m, 9H), 1.17 (qd, *J* = 13.0, 3.9 Hz, 1H), 1.09 (dd, *J* = 12.1, 5.8 Hz, 1H), 1.03 (d, *J* = 6.5 Hz, 3H), 0.98 (td, *J* = 14.1, 3.4 Hz, 1H), 0.93 (s, 3H), 0.71 (s, 3H); ^13^C NMR (151 MHz, (CD_3_)_2_SO) δ 174.36, 174.08, 172.70, 71.16, 70.07, 51.71, 47.55, 46.35, 46.08, 41.70, 36.34, 35.75, 35.22, 35.18, 33.91, 33.02, 32.48, 31.76, 30.84, 30.28, 28.68, 27.32, 27.08, 27.08, 26.20, 23.61, 23.18, 17.18, 12.54; HRMS (ESI) exact mass calculated for [M+H]+ (C_29_H_48_NO_7_) requires m/z 522.3426, found 522.3427 with a difference of 0.19 ppm.

Isoleucine conjugated cholic acid (Ile-CA): Purified according to literature procedure[4](https://www.zotero.org/google-docs/?RBPPH3) and characterization data is consistent with reported data.

Leucine conjugated cholic acid (Leu-CA): Purified according to literature procedure^4^ and characterization data is consistent with reported data.

Methionine conjugated chenodeoxycholic acid (Met-CDCA): NaHCO_3_ was used as the inorganic base. Product was purified using 3-6% CH_3_OH in CH_2_Cl_2_ with 1% acetic acid to obtain a 79% yield as a white amorphous solid. ^1^H NMR 600 MHz, CD_3_OD) δ 4.53 (dd, *J* = 9.3, 4.6 Hz, 1H), 3.79 (q, *J* = 2.9 Hz, 1H), 3.37 (tt, *J* = 10.9, 4.2 Hz, 1H), 2.62 – 2.47 (m, 2H), 2.36 – 2.23 (m, 2H), 2.21 – 2.10 (m, 2H), 2.09 (s, 3H), 2.05 – 1.79 (m, 7H), 1.78 – 1.70 (m, 1H), 1.68 – 1.59 (m, 2H), 1.56 – 1.42 (m, 5H), 1.40 – 1.27 (m, 5H), 1.24 – 1.15 (m, 2H), 1.14-1.06 (m, 1H), 1.03 – 0.94 (m, 4H), 0.93 (s, 3H), 0.69 (s, 3H); ^13^C NMR (151 MHz, CD_3_OD) δ 176.93, 175.25, 72.83, 69.05, 57.34, 52.54, 51.50, 43.66, 43.12, 41.02, 40.73, 40.41, 36.80, 36.54, 36.19, 35.86, 34.01, 33.81, 33.21, 32.10, 31.32, 31.29, 29.28, 24.62, 23.42, 21.78, 18.94, 15.24, 12.23; HRMS (ESI) exact mass calculated for [M+H]+ (C_29_H_50_NO_5_S) requires m/z 524.3404, found 524.3406 with a difference of 0.38 ppm.

Methionine conjugated deoxycholic acid (Met-DCA): NaHCO_3_ was used as the inorganic base. Product was purified using 3-6% CH_3_OH in CH_2_Cl_2_ with 1% acetic acid to obtain a 76% yield as a white amorphous solid. ^1^H NMR (600 MHz, CD_3_OD) δ 4.53 (dd, *J* = 9.3, 4.6 Hz, 1H), 3.96 (t, *J* = 3.0 Hz, 1H), 3.53 (tt, *J* = 11.2, 4.6 Hz, 1H), 2.62 – 2.55 (m, 1H), 2.55 – 2.48 (m, 1H), 2.37 – 2.28 (m, 1H), 2.24 – 2.10 (m, 2H), 2.09 (s, 3H), 1.99 – 1.93 (m, 1H), 1.93 – 1.73 (m, 7H), 1.65 – 1.57 (m, 3H), 1.57 – 1.49 (m, 2H), 1.49 – 1.37 (m, 6H), 1.36 – 1.30 (m, 1H), 1.30 – 1.25 (m, 2H), 1.22 – 1.06 (m, 2H), 1.03 (d, *J* = 6.5 Hz, 3H), 0.98 (td, *J* = 14.2, 3.2 Hz, 1H), 0.93 (s, 3H), 0.71 (s, 3H). ^13^C NMR (151 MHz, CD_3_OD) δ 177.0099, 175.27, 74.06, 72.52, 52.54, 49.24, 48.11, 47.54, 43.58, 37.42, 37.15, 36.72, 36.41, 35.27, 34.78, 33.80, 33.17, 32.11, 31.27, 31.01, 29.86, 28.67, 28.38, 27.43, 24.86, 23.72, 17.68, 15.22, 13.24; HRMS (ESI) exact mass calculated for [M+H]+ (C_29_H_50_NO_5_S) requires m/z 524.3404, found 524.3405 with a difference of 0.19 ppm.

Phenylalanine conjugated cholic acid (Phe-CA): Purified according to literature procedure^4^ and characterization data is consistent with reported data.

Phenylalanine conjugated chenodeoxycholic acid (Phe-CDCA): NaHCO_3_ was used as the inorganic base. Product was purified using 3-6% CH_3_OH in CH_2_Cl_2_ with 1% acetic acid to obtain a 94% yield as a white amorphous solid. ^1^H NMR (600 MHz, CD_3_OD) δ 7.29 – 7.17 (m, 5H), 4.64 (dd, *J* = 9.5, 4.9 Hz, 1H), 3.79 (q, *J* = 2.9 Hz, 1H), 3.41 – 3.33 (m, 1H), 3.22 (dd, *J* = 14.0, 4.8 Hz, 1H), 2.93 (dd, *J* = 13.9, 9.5 Hz, 1H), 2.27 (q, *J* = 11.7 Hz, 1H), 2.23 – 2.17 (m, 1H), 2.10 – 2.03 (m, 1H), 1.98 – 1.94 (m, 1H), 1.90 – 1.81 (m, 3H), 1.76 – 1.59 (m, 4H), 1.55 – 1.43 (m, 4H), 1.40 – 1.27 (m, 5H), 1.26 – 1.04 (m, 5H), 1.02 – 0.94 (m, 1H), 0.94 – 0.91 (m, 6H), 0.66 (s, 3H); ^13^C NMR (151 MHz, (CD_3_)_2_SO) δ 173.41, 172.64, 137.99, 129.15, 128.10, 126.32, 70.41, 66.23, 55.60, 53.59, 50.05, 41.94, 41.47, 36.87, 35.36, 35.01, 34.87, 34.78, 32.34, 32.15, 31.54, 30.59, 27.79, 23.22, 22.77, 20.31, 18.35, 11.70; HRMS (ESI) exact mass calculated for [M+H]+ (C_33_H_50_NO_5_) requires m/z 540.3684, found 540.3683 with a difference of 0.19 ppm.

Phenylalanine conjugated deoxycholic acid (Phe-DCA): NaHCO_3_ was used as the inorganic base. Product was purified using 3-6% CH_3_OH in CH_2_Cl_2_ with 1% acetic acid to obtain a 99% yield as a white amorphous solid. ^1^H NMR (600 MHz, CD_3_OD) δ 7.30 – 7.17 (m, 5H), 4.65 (dd, *J* = 9.3, 4.9 Hz, 1H), 3.94 (t, *J* = 3.0 Hz, 1H), 3.57 – 3.48 (m, 1H), 3.21 (dd, *J* = 13.9, 4.9 Hz, 1H), 2.94 (dd, *J* = 13.9, 9.3 Hz, 1H), 2.25 – 2.17 (m, 1H), 2.11 – 2.03 (m, 1H), 1.95 – 1.84 (m, 2H), 1.85 – 1.74 (m, 4H), 1.71 – 1.62 (m, 1H), 1.64 – 1.55 (m, 3H), 1.54 – 1.48 (m, 2H), 1.50 – 1.32 (m, 6H), 1.31 – 1.24 (m, 1H), 1.24 – 1.11 (m, 3H), 1.12 – 1.01 (m, 1H), 1.02 – 0.93 (m, 4H), 0.93 (s, 3H), 0.67 (s, 3H); ^13^C NMR (151 MHz, (CD_3_)_2_SO) δ 173.42, 172.74, 137.97, 129.15, 128.11, 126.33, 71.11, 70.04, 53.56, 48.66, 47.52, 46.22, 46.01, 41.68, 36.86, 36.33, 35.72, 35.21, 35.08, 33.87, 32.98, 32.27, 31.63, 30.27, 28.66, 27.21, 27.05, 26.18, 23.57, 23.14, 21.14, 17.10, 12.49; HRMS (ESI) exact mass calculated for [M+H]+ (C_33_H_50_NO_5_) requires m/z 540.3684, found 540.3684 with a difference of 0.00 ppm.

Threonine conjugated chollc acid (Thr-CA): NaHCO_3_ was used as the inorganic base. Product was purified using 6-12% CH_3_OH in CH_2_Cl_2_ with 1% acetic acid to obtain a 97% yield as a white amorphous solid. ^1^H NMR (600 MHz, CD_3_OD) δ 4.37 (s, 1H), 4.28 (s, 1H), 3.96 (t, *J* = 3.0 Hz, 1H), 3.80 (q, *J* = 3.0 Hz, 1H), 3.37 (tt, *J* = 11.4, 4.5 Hz, 1H), 2.39 (ddd, *J* = 14.7, 10.1, 5.1 Hz, 1H), 2.33 – 2.19 (m, 3H), 2.04 – 1.96 (m, 2H), 1.96 – 1.79 (m, 4H), 1.78 – 1.71 (m, 1H), 1.68 – 1.63 (m, 1H), 1.63 – 1.51 (m, 5H), 1.48 – 1.28 (m, 5H), 1.18 (d, *J* = 6.3 Hz, 3H), 1.16 – 1.07 (m, 1H), 1.05 (d, *J* = 6.5 Hz, 3H), 0.98 (td, *J* = 14.2, 3.5 Hz, 1H), 0.92 (s, 3H), 0.72 (s, 3H). ^13^C NMR (151 MHz, CD_3_OD) δ 176.98, 74.04, 72.86, 69.08, 68.75, 48.08, 47.48, 43.15, 42.96, 40.97, 40.42, 36.91, 36.46, 35.87, 35.82, 34.08, 33.21, 31.14, 29.52, 28.68, 27.84, 24.22, 23.14, 20.26, 17.77, 13.00; ^13^C NMR (151 MHz, (CD_3_)_2_SO) δ 173.13, 71.28, 70.66, 66.73, 66.47, 46.41, 45.93, 41.67, 41.52, 35.46, 35.43, 35.02, 34.55, 32.72, 31.97, 30.50, 28.67, 27.51, 26.36, 23.01, 22.77, 19.92, 17.29, 12.52; HRMS (ESI) exact mass calculated for [M+H]+ (C_28_H_48_NO_7_) requires m/z 510.3426, found 510.3424 with a difference of 0.39 ppm.

Tyrosine conjugated cholic acid (Tyr-CA): Purified according to literature procedure^4^ and characterization data is consistent with reported data.

Tyrosine conjugated chenodeoxycholic acid (Tyr-CDCA): NaOH was used as the inorganic base. Product was purified using 3-12% CH_3_OH in CH_2_Cl_2_ with 1% acetic acid to obtain a 74% yield as a white amorphous solid. ^1^H NMR (600 MHz, CD_3_OD) δ 7.03 (d, *J* = 8.5 Hz, 2H), 6.69 (d, *J* = 8.5 Hz, 2H), 4.58 (dd, *J* = 9.3, 4.9 Hz, 1H), 3.80 (q, *J* = 3.0 Hz, 1H), 3.41 – 3.33 (m, 1H), 3.11 (dd, *J* = 14.0, 4.9 Hz, 1H), 2.84 (dd, *J* = 14.0, 9.3 Hz, 1H), 2.31 – 2.17 (m, 2H), 2.11 – 2.02 (m, 1H), 2.02 – 1.93 (m, 2H), 1.91 – 1.81 (m, 3H), 1.77 – 1.58 (m, 4H), 1.55 – 1.43 (m, 4H), 1.43 – 1.04 (m, 9H), 0.98 (td, *J* = 14.2, 3.4 Hz, 1H), 0.93 (t, *J* = 3.3 Hz, 6H), 0.67 (s, 3H). ^13^C NMR (151 MHz, CD_3_OD) δ 176.67, 175.21, 157.21, 131.22, 129.19, 116.15, 72.85, 69.10, 57.31, 55.23, 51.49, 43.64, 43.13, 41.01, 40.72, 40.43, 37.62, 36.80, 36.52, 36.19, 35.85, 34.02, 33.84, 33.23, 31.32, 29.20, 24.61, 23.38, 21.76, 18.85, 12.17; HRMS (ESI) exact mass calculated for [M+H]+ (C_33_H_50_NO_6_) requires m/z 556.3633, found 556.3634 with a difference of 0.18 ppm.

**Supplementary Tables**

**Supplementary Table 1: Number of unique spectral matches per compound for Q-ToF data in positive ionization mode.**

|  | aMCA | bMCA | CA | CDCA | DCA | gMCA | HDCA | UDCA |
| --- | --- | --- | --- | --- | --- | --- | --- | --- |
| Ala | 241 | 4 | 342 | 418 | 420 | 74 | 264 | 40 |
| Arg | 180 | 101 | 409 | 38 | 38 | 103 | 44 | 72 |
| Asn | 152 | 159 | 55 | 2 | 35 | 159 | 0 | 2 |
| Asp | 39 | 36 | 39 | 0 | 8 | 39 | 2 | 0 |
| Cit | 33 | 33 | 64 | 224 | 226 | 33 | 224 | 224 |
| Cys | 0 | N/A | 4 | 0 | 0 | 0 | 0 | 0 |
| DOPA | 0 | 0 | 0 | 0 | 0 | 0 | 0 | 0 |
| Gln | 13 | 8 | 20 | 4 | 4 | 11 | 4 | 4 |
| Glu | 77 | 48 | 181 | 164 | 170 | 76 | 137 | N/A |
| His | 720 | 673 | 605 | 242 | 239 | 724 | 244 | 244 |
| Ile/Leu | 982 | 601 | 757 | 1563 | 1590 | 920 | 1376 | 1420 |
| Lys | 1181 | 963 | 1250 | 551 | 579 | 672 | 548 | 626 |
| Met | 115 | 115 | 10 | 204 | 214 | 115 | 217 | 207 |
| Orn | 305 | 199 | 215 | 80 | 83 | 203 | 73 | 84 |
| Phe | 1658 | 1453 | 1507 | 2629 | 2629 | 1544 | 2517 | 2624 |
| Pro | 0 | 0 | 0 | 0 | 0 | 0 | 0 | 0 |
| Ser | 1 | 3 | 44 | 30 | 32 | 24 | 30 | 30 |
| Thr | 349 | 328 | 139 | 7 | 22 | 340 | 0 | 0 |
| Trp | 446 | 420 | 518 | 1286 | 1286 | 502 | 1283 | 1283 |
| Tyr | 568 | 465 | 563 | 431 | 431 | 534 | 412 | 436 |
| Val | 0 | 0 | 48 | 8 | 4 | 0 | 4 | 4 |

**Supplementary Table 2: Number of unique spectral matches per compound for Orbitrap data in positive ionization mode.**

|  | aMCA | bMCA | CA | CDCA | DCA | gMCA | HDCA | UDCA |
| --- | --- | --- | --- | --- | --- | --- | --- | --- |
| Ala | 27 | 27 | 68 | 139 | 301 | 27 | 165 | 45 |
| Arg | 217 | 235 | 366 | 46 | 51 | 217 | 46 | 48 |
| Asn | 152 | 152 | 28 | 6 | 4 | 152 | 4 | 4 |
| Asp | 36 | 36 | 3 | 2 | 8 | 36 | 2 | 2 |
| Cit | 2 | 2 | 34 | 188 | 187 | 2 | 188 | 188 |
| Cys | N/A | N/A | N/A | N/A | 0 | N/A | N/A | N/A |
| DOPA | 0 | 0 | 0 | 0 | 0 | 0 | 0 | 0 |
| Gln | 8 | 2 | 15 | 2 | 2 | 8 | 2 | 2 |
| Glu | 51 | 36 | 113 | 149 | 178 | 111 | 136 | N/A |
| His | 595 | 591 | 595 | 244 | 244 | 595 | 244 | 225 |
| Ile/Leu | 595 | 574 | 712 | 1476 | 1548 | 584 | 1434 | 1414 |
| Lys | 517 | 464 | 856 | 548 | 570 | 141 | 542 | 548 |
| Met | 115 | 97 | 10 | 199 | 198 | 115 | 198 | 198 |
| Orn | 203 | 196 | 422 | 80 | 83 | 23 | 36 | 83 |
| Phe | 1509 | 639 | 1407 | 2513 | 2513 | 1274 | 2450 | 2526 |
| Pro | 0 | 0 | 0 | 0 | 0 | 0 | 0 | 0 |
| Ser | 8 | 4 | 37 | 36 | 32 | 11 | 28 | 30 |
| Thr | 231 | 235 | 108 | 5 | 35 | 229 | 5 | 10 |
| Trp | 499 | 471 | 506 | 1283 | 1284 | 495 | 1268 | 1283 |
| Tyr | 486 | 361 | 563 | 333 | 324 | 355 | 310 | 333 |
| Val | 0 | 15 | 16 | 26 | 42 | 0 | 4 | 4 |

**Supplementary Table 3: Number of unique spectral matches per compound for Q-ToF data in negative ionization mode.** There is very little negative mode data in the public domain resulting in very few matches.

|  | aMCA | bMCA | CA | CDCA | DCA | gMCA | HDCA | UDCA |
| --- | --- | --- | --- | --- | --- | --- | --- | --- |
| Ala | 0 | 0 | 0 | 0 | 0 | 0 | 0 | 0 |
| Arg | 0 | 0 | 0 | 0 | 0 | 0 | 0 | 0 |
| Asn | 0 | 0 | 0 | 0 | 2 | 0 | 0 | 0 |
| Asp | 0 | 0 | 0 | 0 | 0 | 0 | 0 | 0 |
| Cit | 0 | 0 | 0 | 0 | 0 | 0 | 0 | 0 |
| Cys | 0 | 0 | 0 | 0 | 0 | 0 | 0 | 0 |
| DOPA | 0 | 0 | 0 | 0 | 0 | 0 | 0 | 0 |
| Gln | 0 | 0 | 0 | 0 | 0 | 0 | 0 | 0 |
| Glu | 0 | 0 | 0 | 0 | 0 | 0 | 0 | 0 |
| His | 0 | 0 | 0 | 0 | 0 | 0 | 0 | 0 |
| Ile/Leu | 0 | 0 | 0 | 0 | 0 | 0 | 0 | 0 |
| Lys | 0 | 0 | 0 | 0 | 0 | 0 | 0 | 0 |
| Met | 0 | 0 | 0 | 0 | 0 | 0 | 0 | 0 |
| Orn | 0 | 0 | 0 | 0 | 0 | 0 | 0 | 0 |
| Phe | 0 | 0 | 0 | 0 | 0 | 0 | 0 | 4 |
| Pro | 0 | 0 | 0 | 0 | 0 | 0 | 0 | 0 |
| Ser | 0 | 0 | 0 | 0 | 0 | 0 | 0 | 0 |
| Thr | 0 | 0 | 0 | 0 | 0 | 0 | 0 | 0 |
| Trp | 0 | 0 | 0 | 0 | 0 | 0 | 0 | 0 |
| Tyr | 0 | 0 | 0 | 0 | 0 | 0 | 0 | 2 |
| Val | 0 | 0 | 0 | 0 | 0 | 0 | 0 | 0 |

**Supplementary Table 4: The collision values used for the MS/MS data acquisition on the QToF.**

**Supplementary Table 5: Universal Spectrum Identifiers (USIs) and MASST job IDs for all synthesized compounds.**

**Supplementary Table 6: Precursor masses, mass accuracies and retention times for conjugated bile acids detected in iHMP2 stool samples on a Thermo QE Orbitrap in negative mode.**

**Supplementary Table 7: Relative abundances of conjugated bile acids across bacterial strains.**

**Supplementary Table 8: Precursor masses, mass accuracies and retention times for conjugated bile acids detected in bacterial cultures on a Bruker Q-ToF in positive mode.**

**Supplementary Table 9: Representative precursor values, ppm mass accuracies, retention times, CCS values and key confirmatory MS/MS fragments from conjugated bile acids detected in bacterial cultures on Agilent 6560 IM-QTOF MS instrument in negative mode.**

**Supplementary Table 10: Precursor masses, mass accuracies and retention times for conjugated bile acids detected in human IBD fecal samples on a Bruker Q-ToF in positive mode.**

**Supplementary Data**

**^1^H and ^13^C NMR spectra of pure synthesized conjugated bile acids made by the Dorrestein/Siegel labs at UC-San Diego.
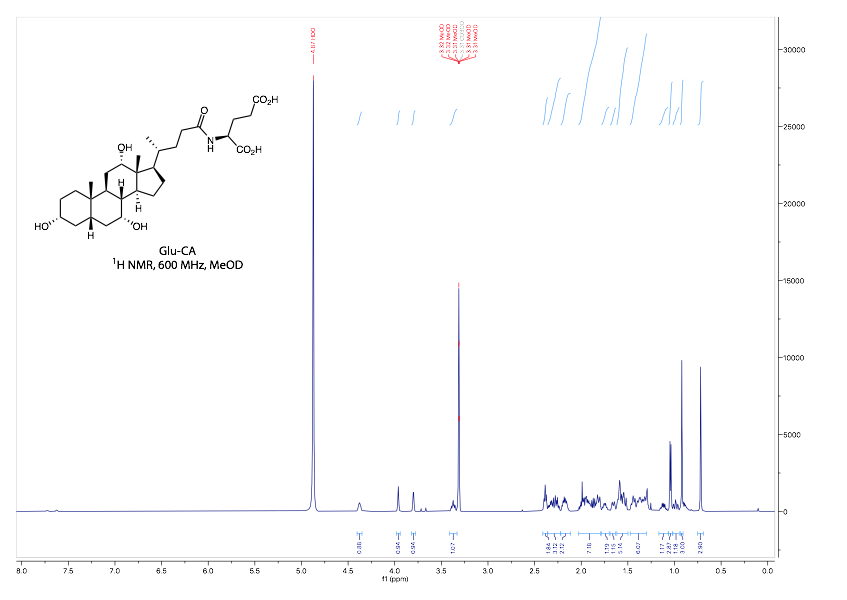

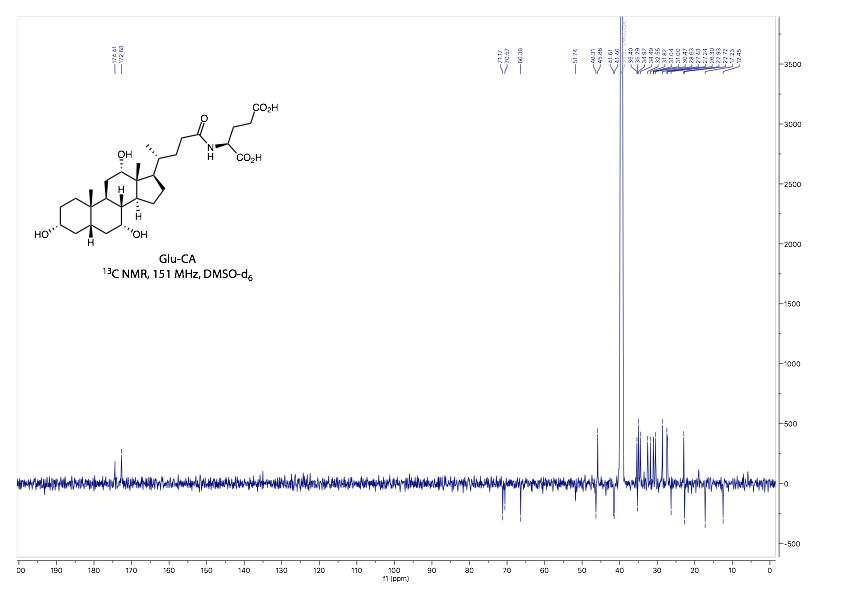
**

**
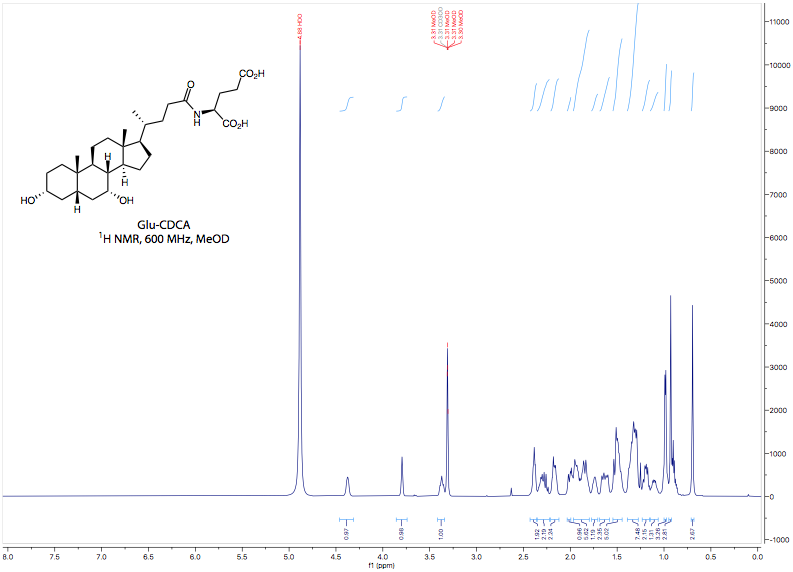

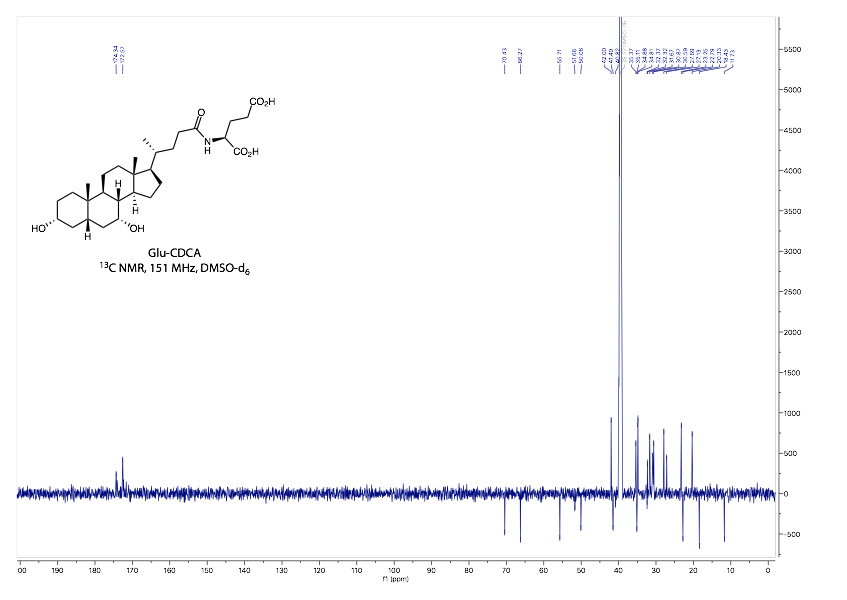
**

**
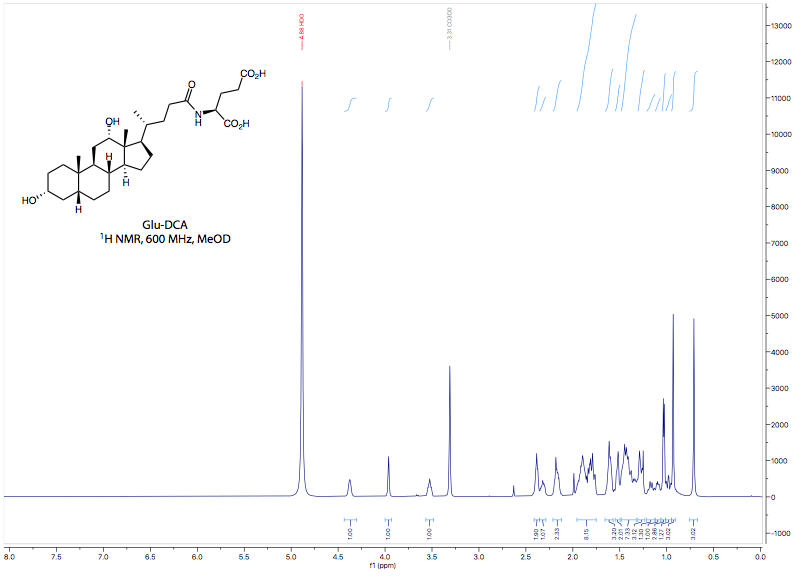

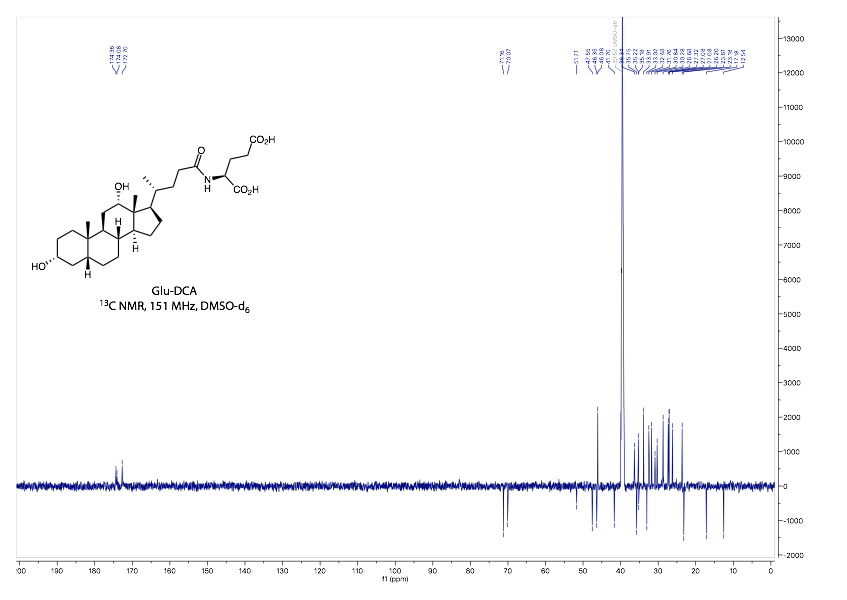

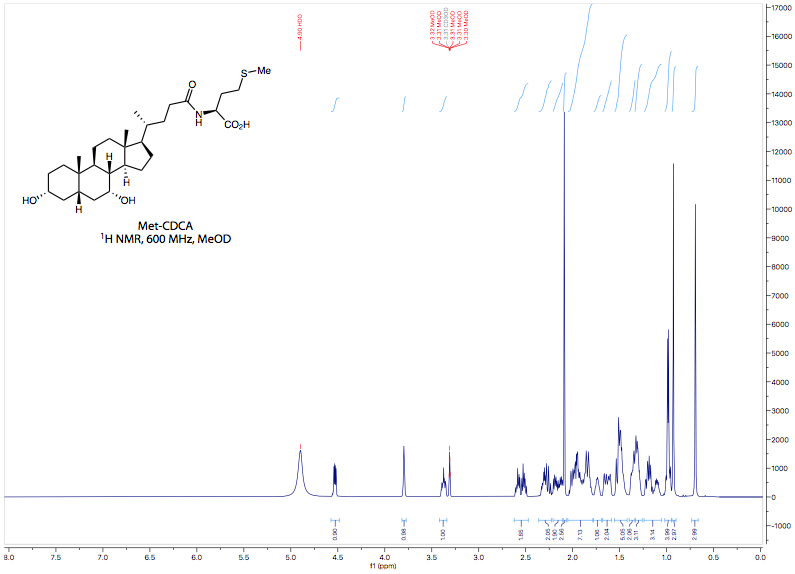

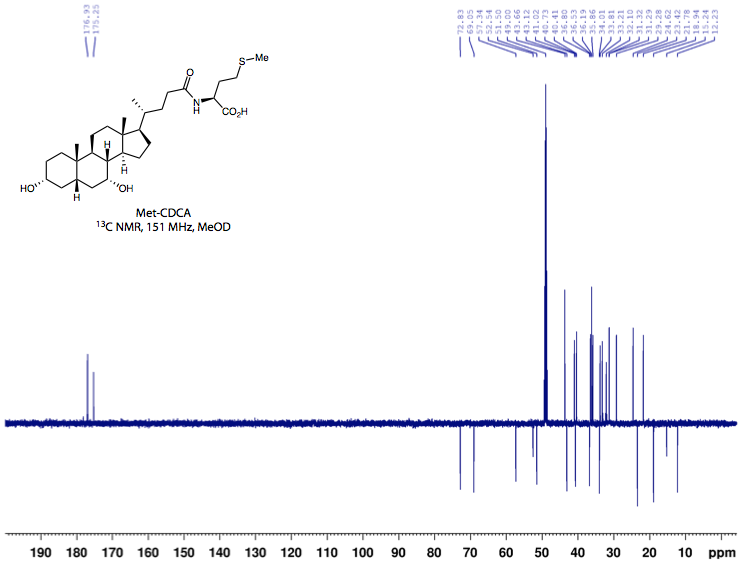
**

**
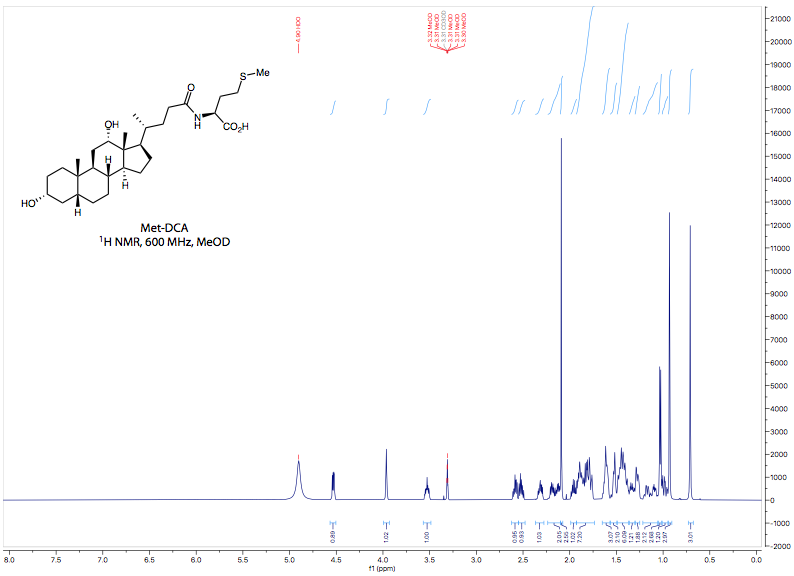
**

**
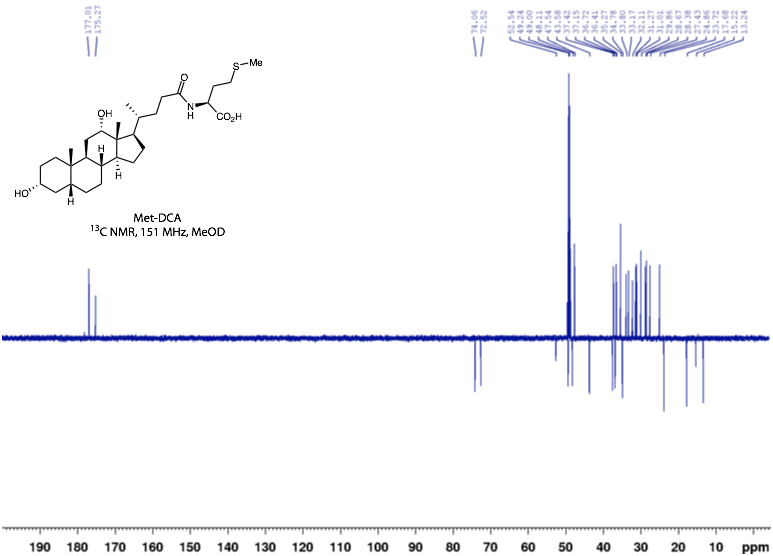
**

**
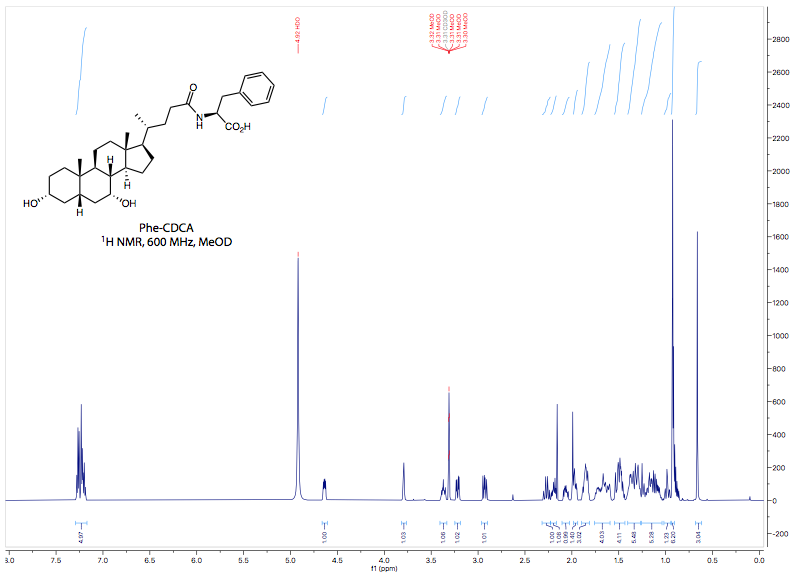
**

**
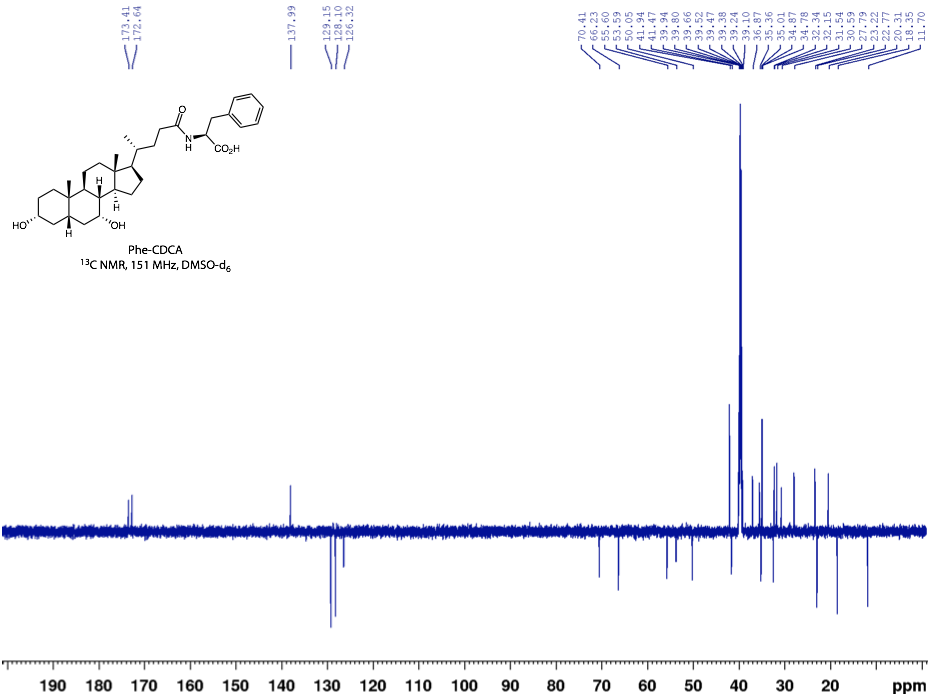
**

**
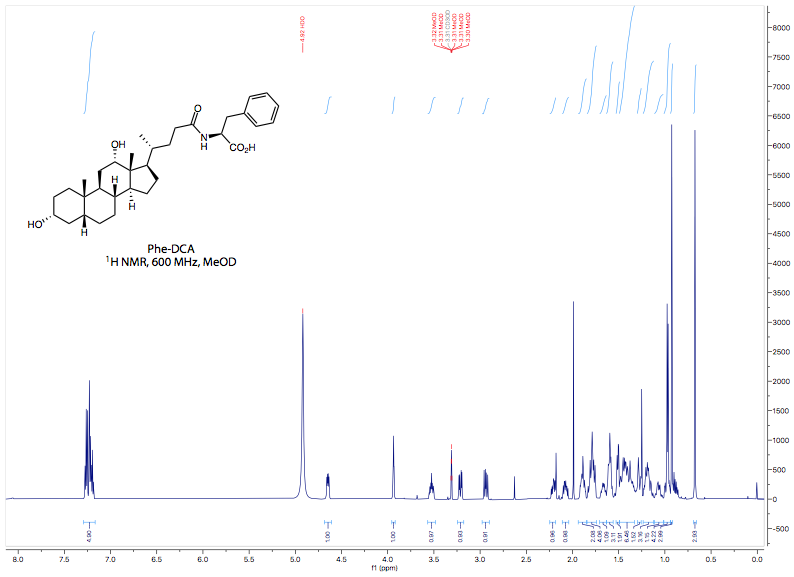

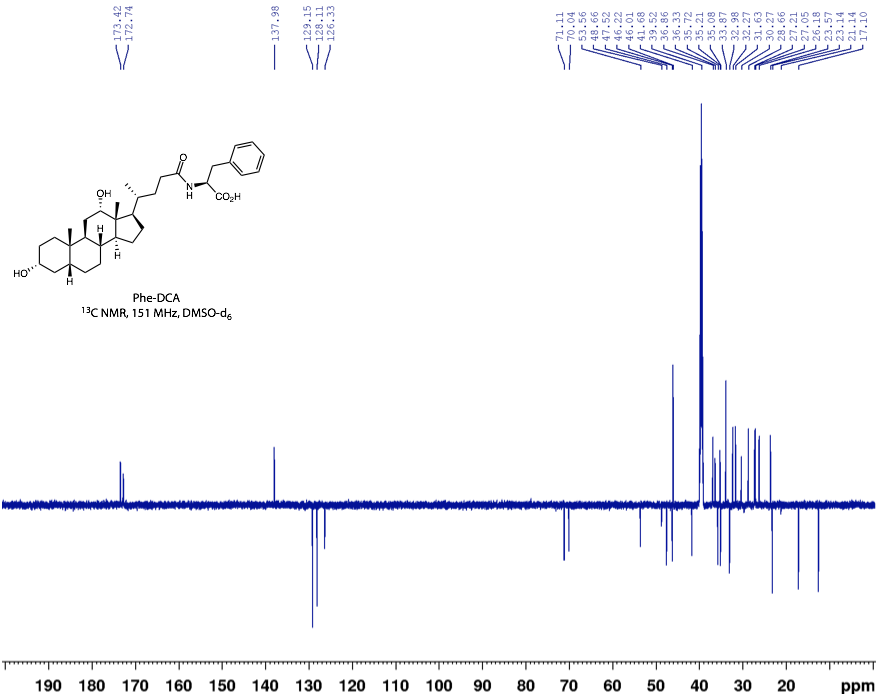
**
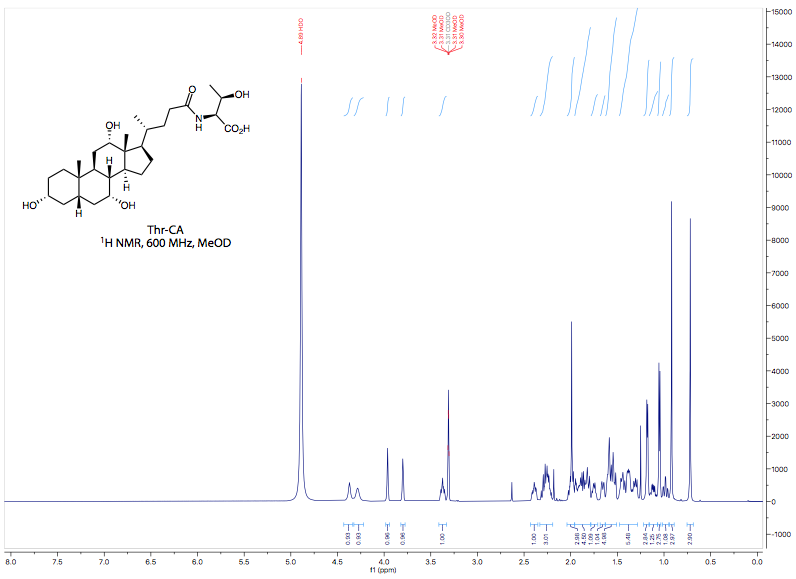

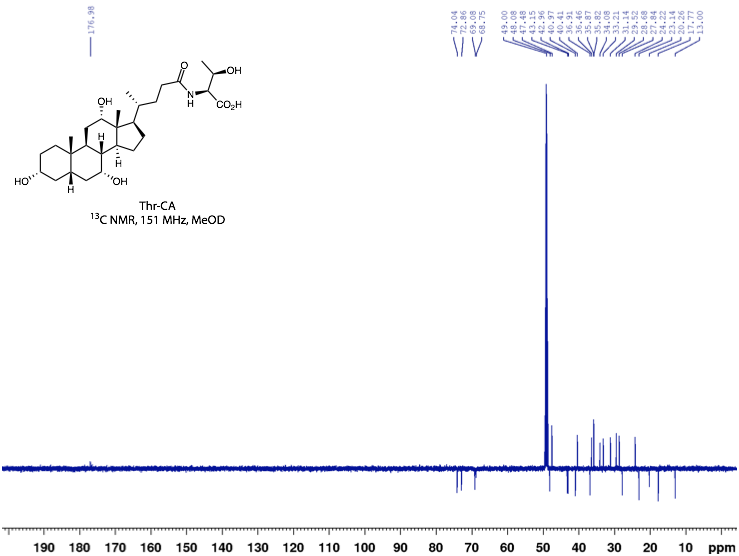

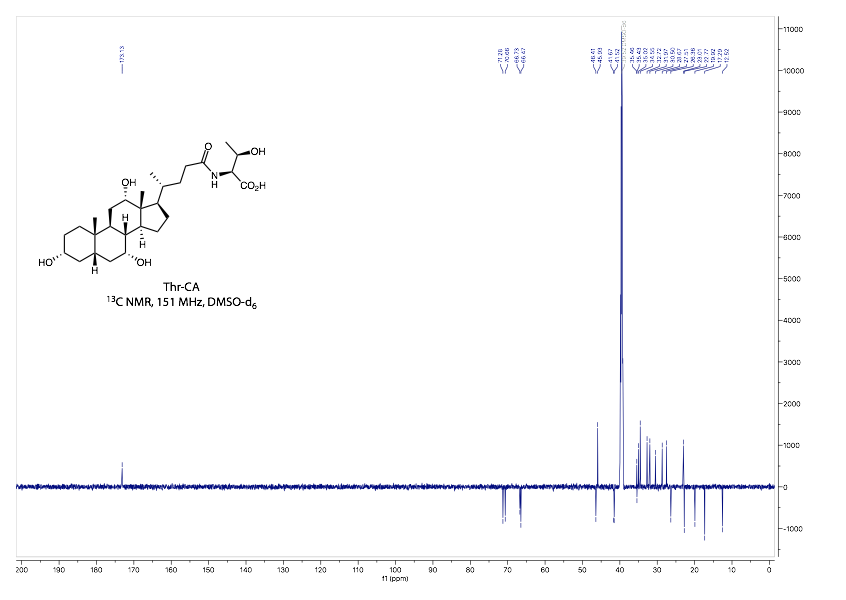


**
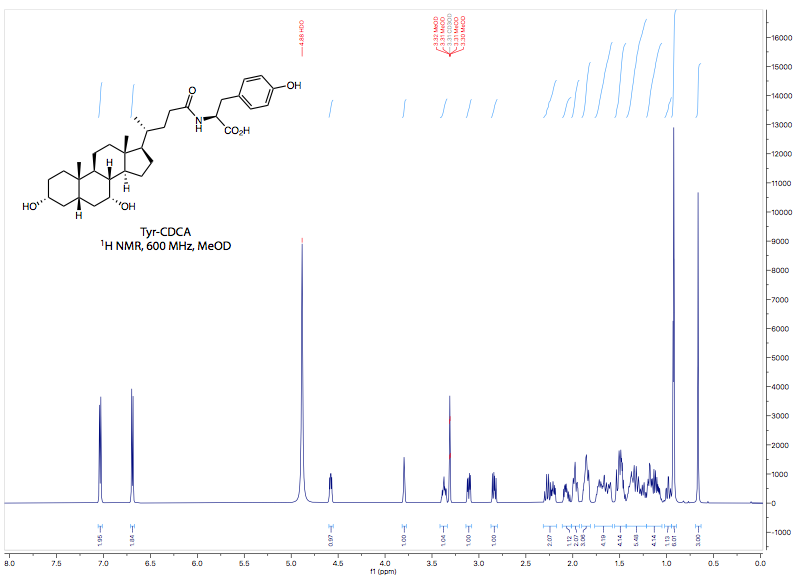

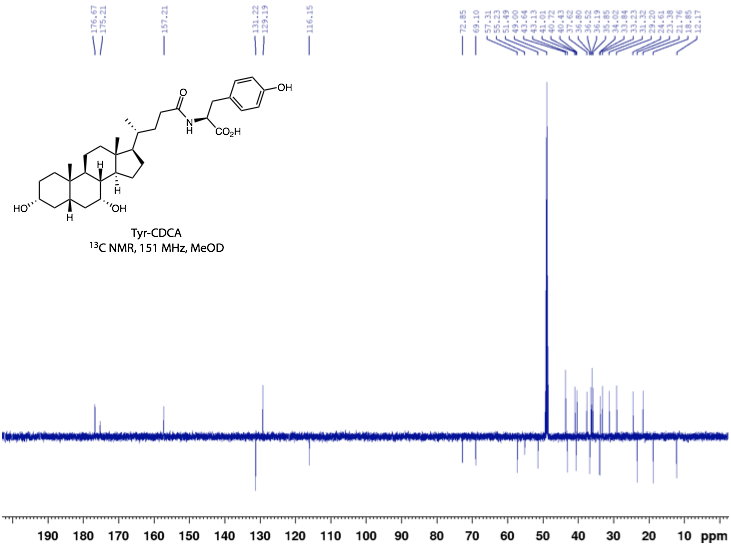
**

**NMR of pure synthetic standards made by the Patterson lab at Penn State.**


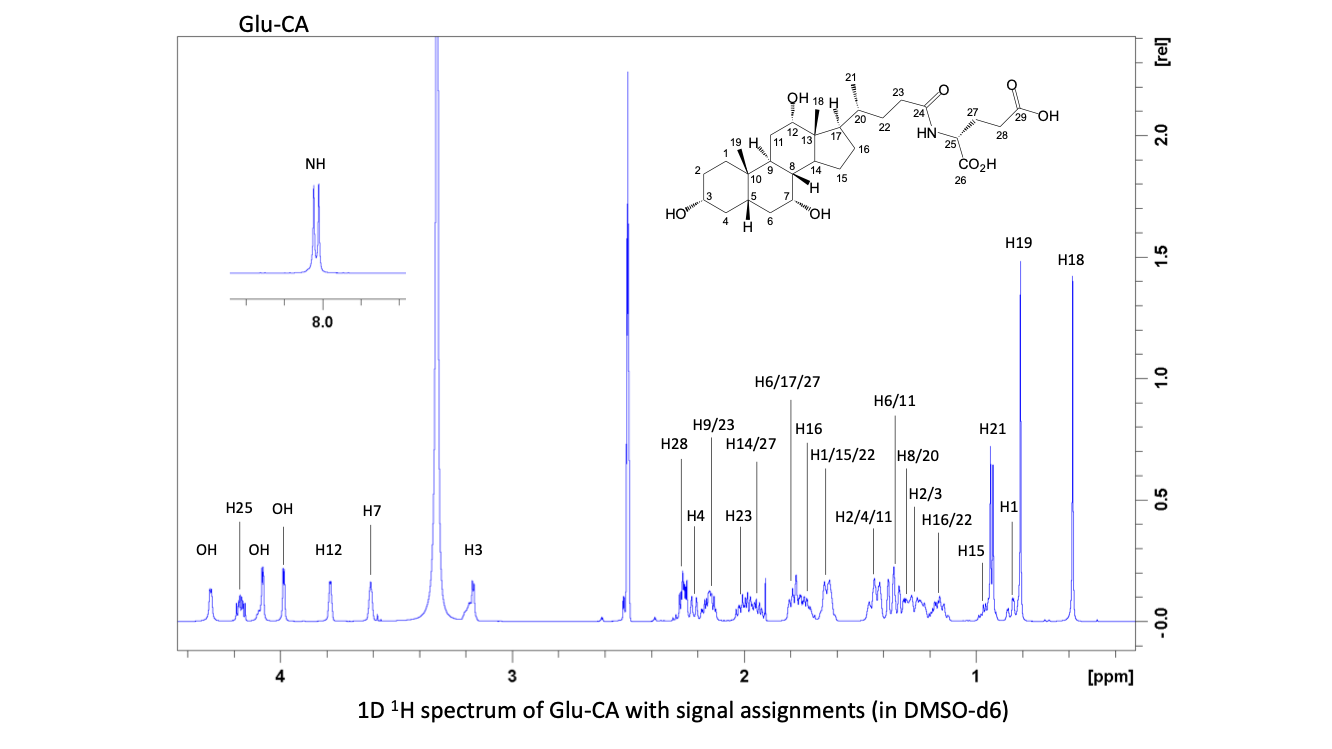


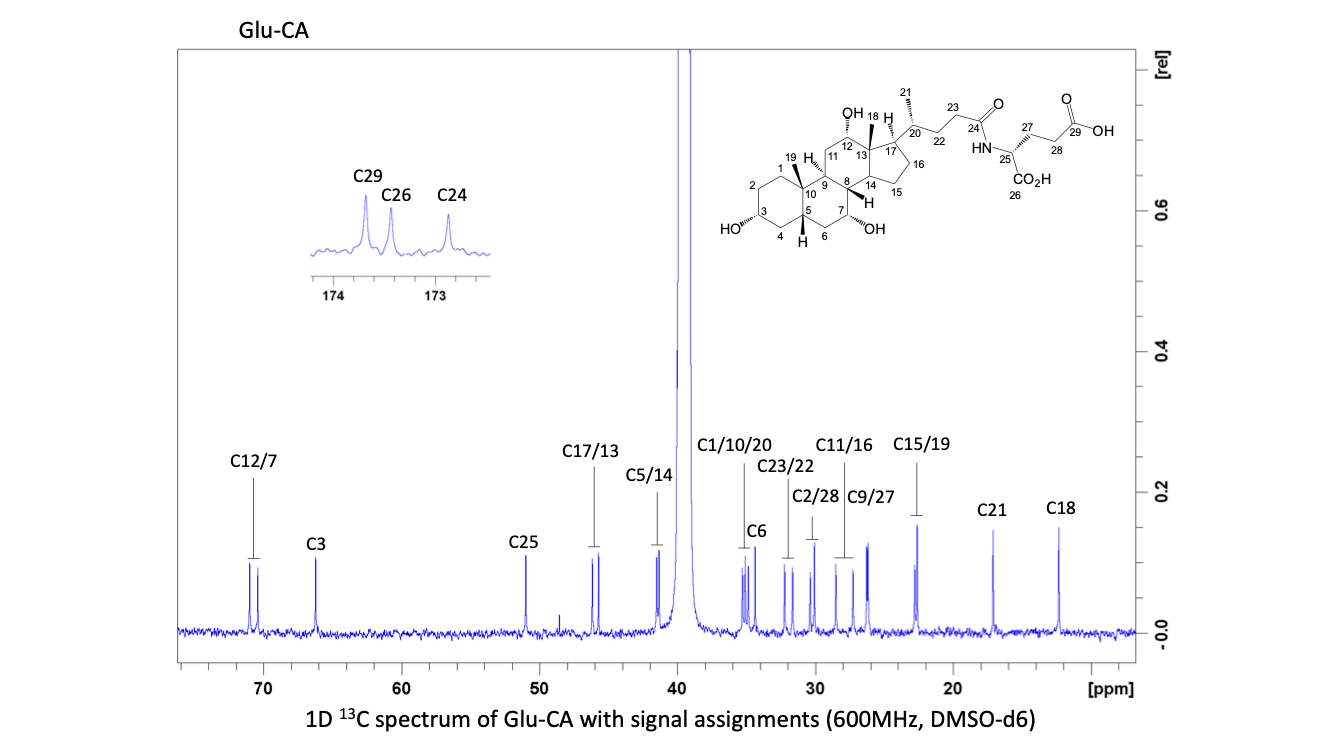


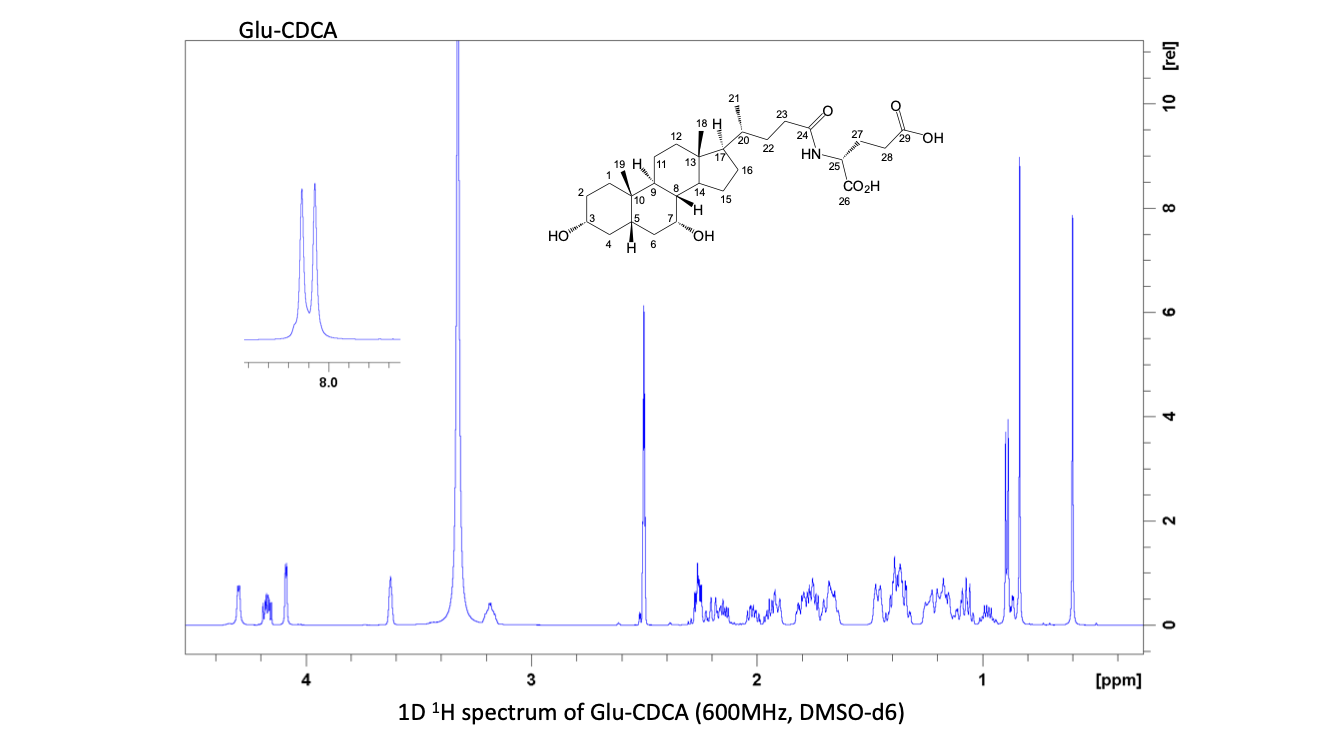


**
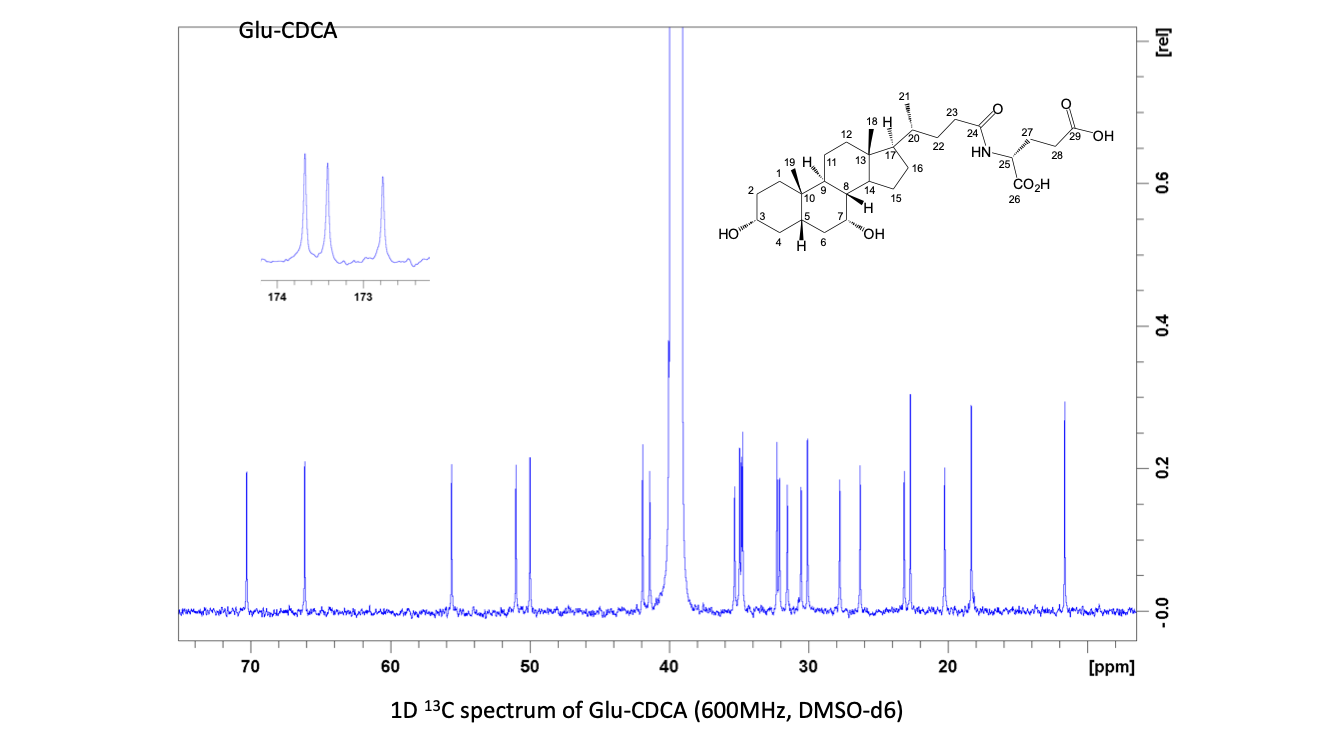
**

**
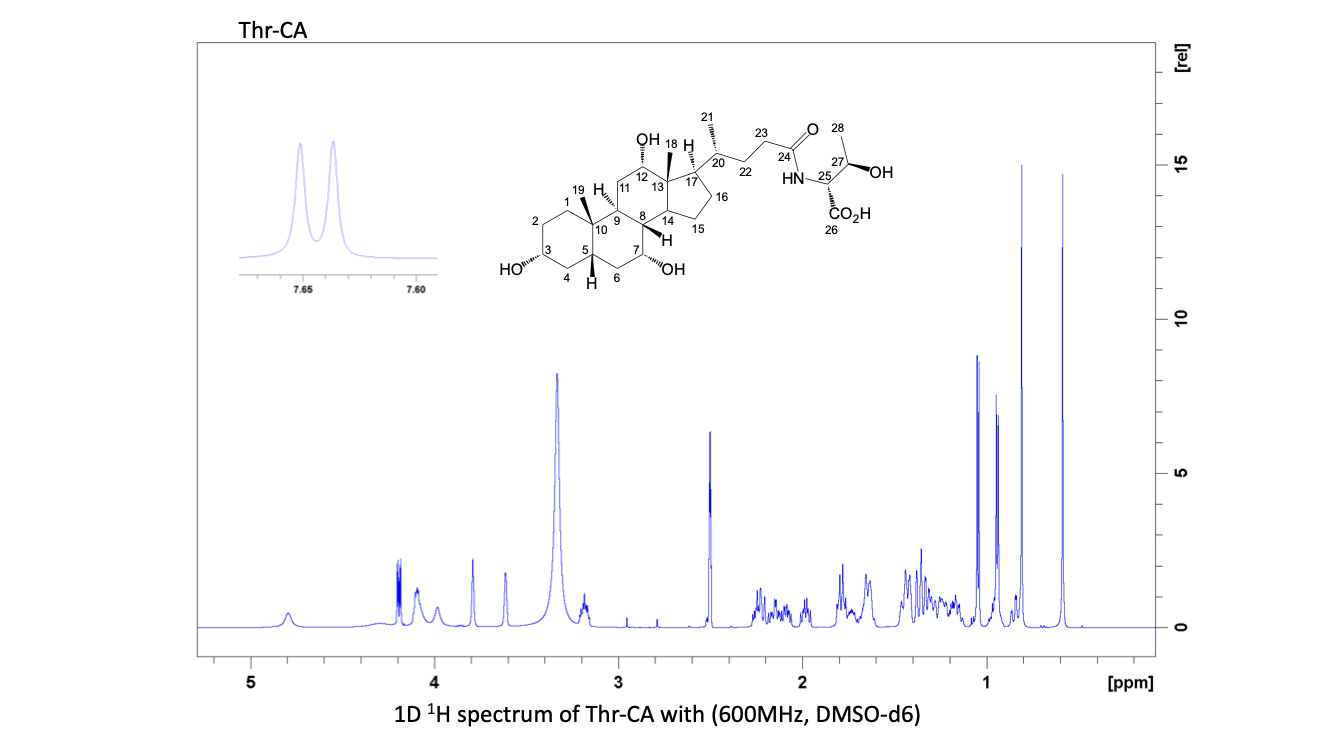
** **
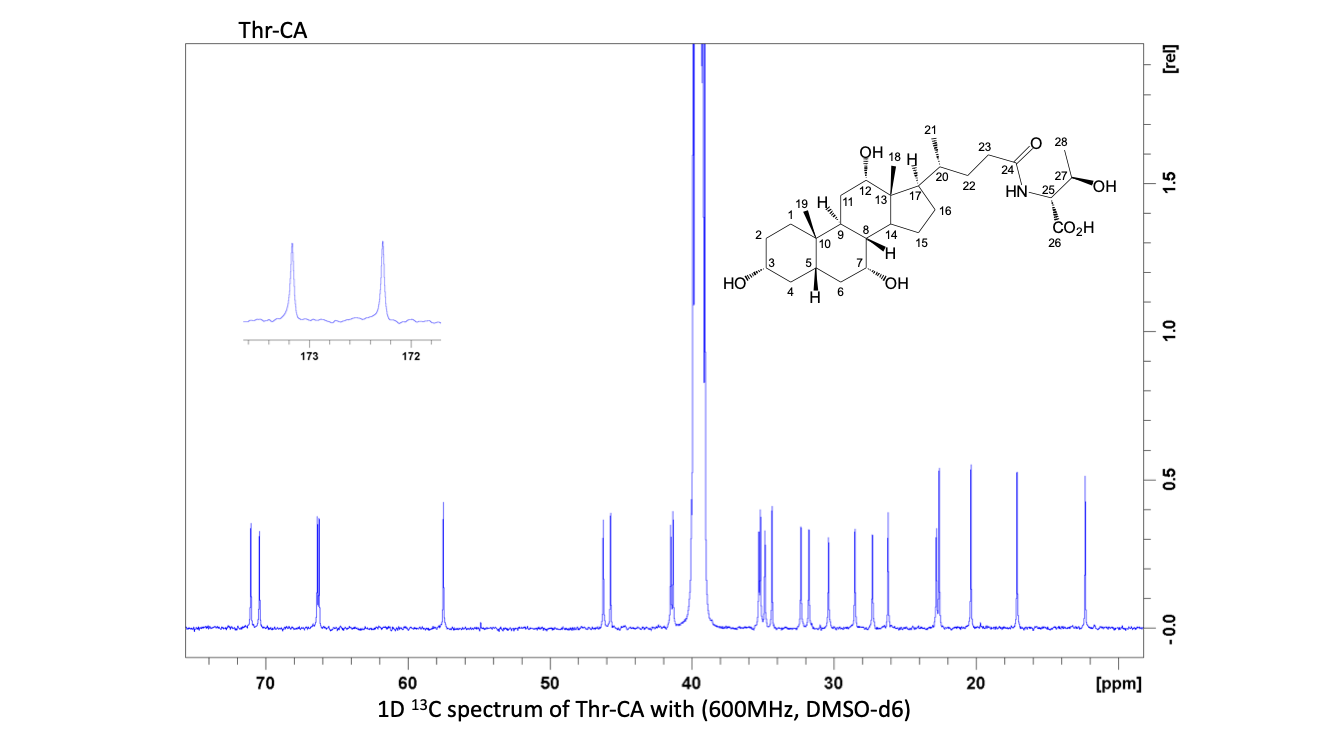
**

**
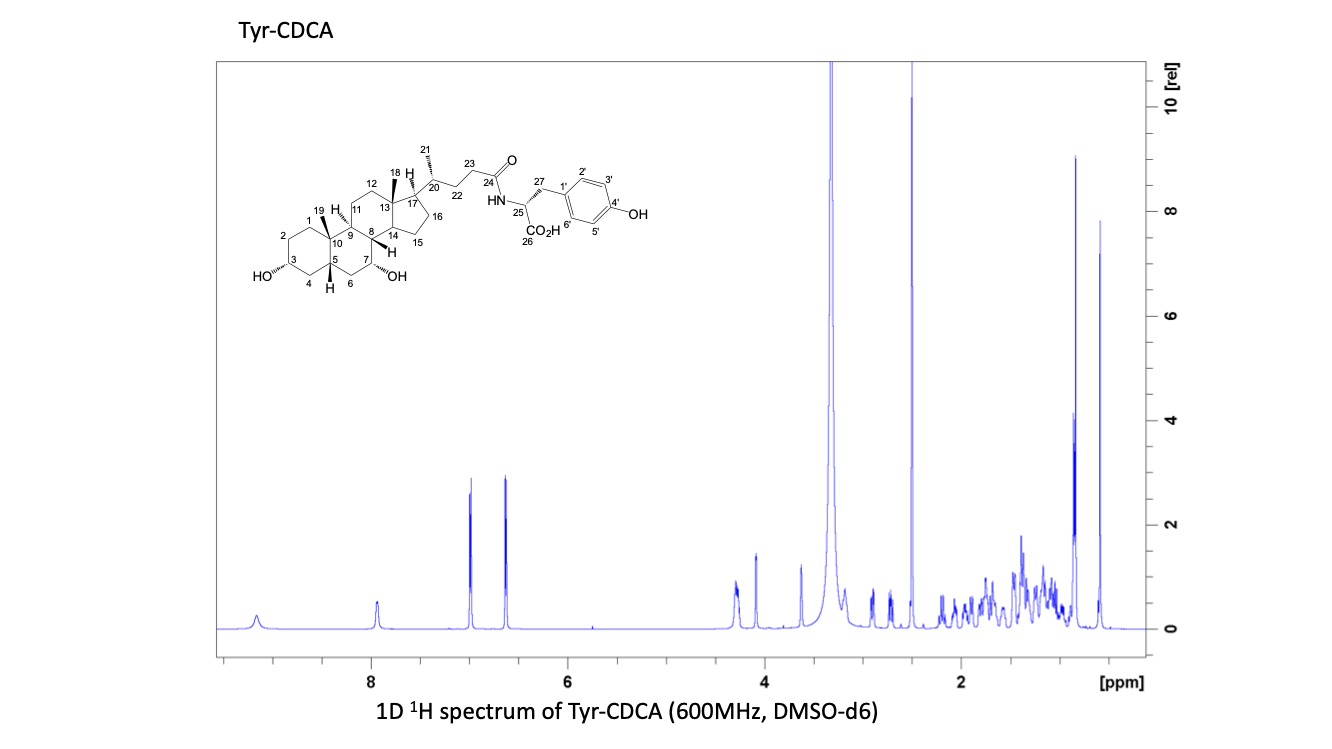
**

**
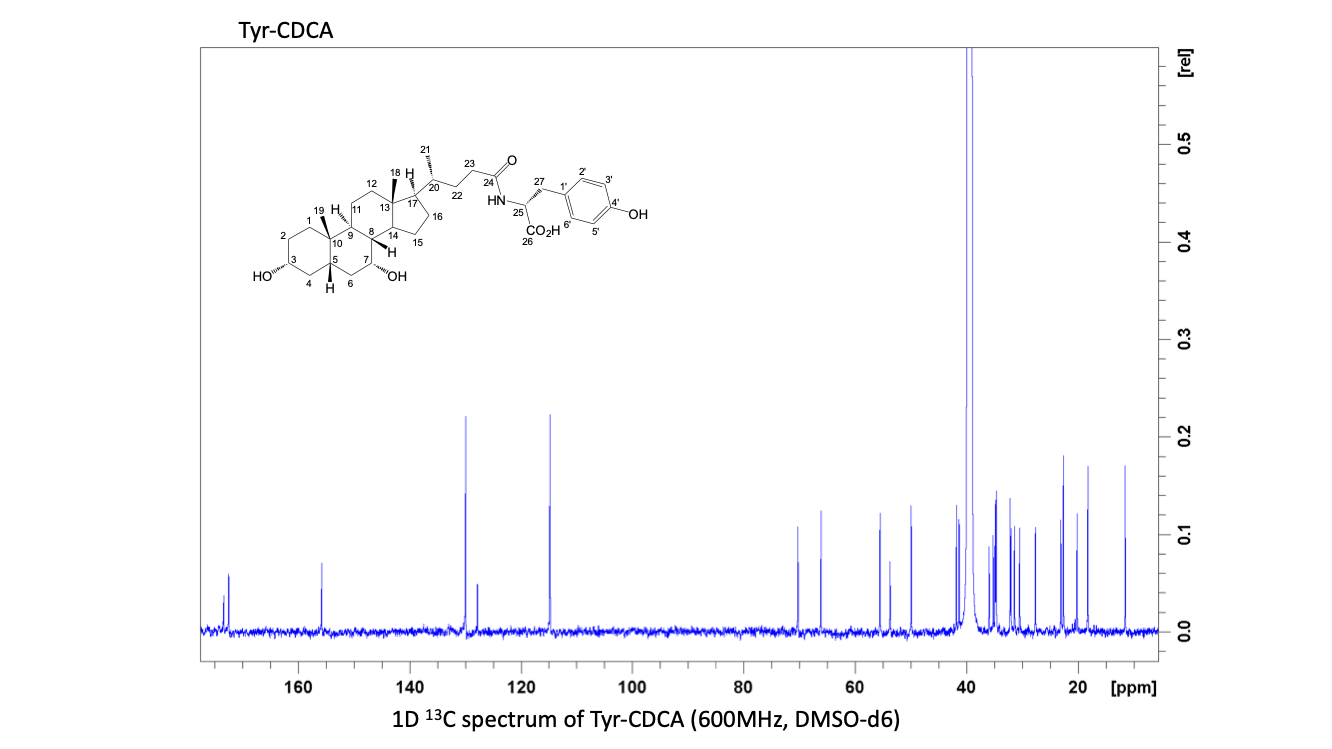
**

**Gating Strategy for Flow Cytometry**

**
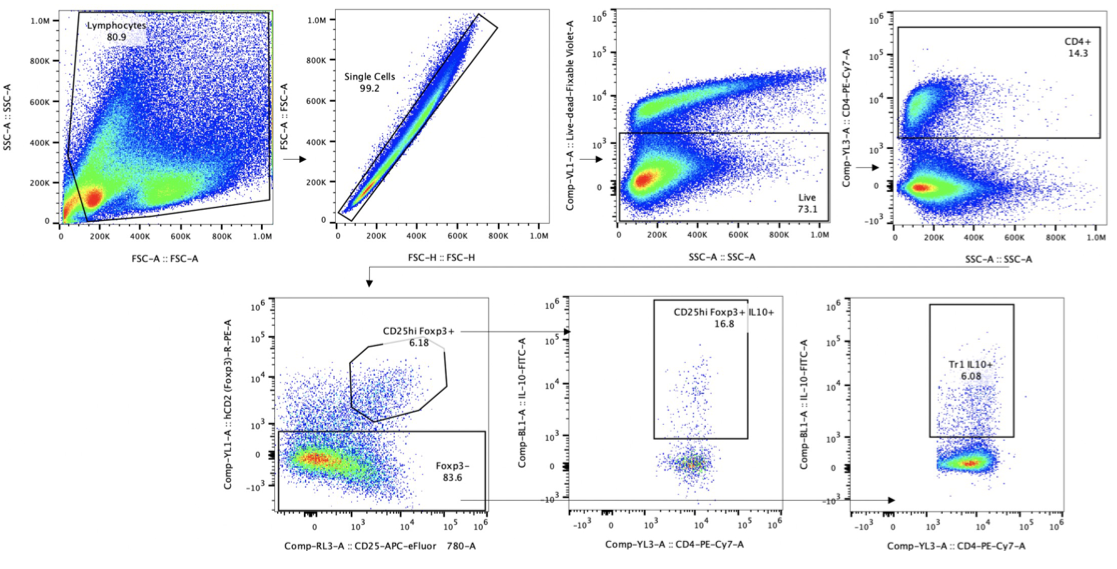
**

**Supplementary References**

[1. Katajamaa, M., Miettinen, J. & Oresic, M. MZmine: toolbox for processing and visualization of mass spectrometry based molecular profile data. Bioinforma. Oxf. Engl. 22, 634–636 (2006).](https://www.zotero.org/google-docs/?cYk7cw)

[2. Pluskal, T., Castillo, S., Villar-Briones, A. & Orešič, M. MZmine 2: Modular framework for processing, visualizing, and analyzing mass spectrometry-based molecular profile data.](https://www.zotero.org/google-docs/?cYk7cw) *[BMC Bioinformatics](https://www.zotero.org/google-docs/?cYk7cw)* **[11](https://www.zotero.org/google-docs/?cYk7cw)**[, 395 (2010).](https://www.zotero.org/google-docs/?cYk7cw)

[3. McDonald, J. A. K. et al. Evaluation of microbial community reproducibility, stability and composition in a human distal gut chemostat model. J. Microbiol. Methods 95, 167–174 (2013).](https://www.zotero.org/google-docs/?cYk7cw)

[4. Quinn, R. A.](https://www.zotero.org/google-docs/?cYk7cw) *[et al.](https://www.zotero.org/google-docs/?cYk7cw)* [Global chemical effects of the microbiome include new bile-acid conjugations.](https://www.zotero.org/google-docs/?cYk7cw) *[Nature](https://www.zotero.org/google-docs/?cYk7cw)* **[579](https://www.zotero.org/google-docs/?cYk7cw)**[, 123–129 (2020).](https://www.zotero.org/google-docs/?cYk7cw)
