# Supplementary material for: Reverse metabolomics for the discovery of chemical structures from humans
Source: Nature. Author manuscript; Available in PMC 2024 Mar 8. (PMC10849969; doi:10.1038/s41586-023-06906-8)

Conjugated bile acids found in HMP bacterial cultures

Bile Acid	predicted m/z $[M+H]^+$	observed m/z $[M+H]^+$	abs. ppm diff	standard RT (min)	observed RT (min)
Ala-CA	480.3321	480.3326	1.04	3.9	3.9
Ala-DCA	464.3371	464.3375	0.86	4.8	4.8
Arg-CA	565.3961	565.3962	0.18	3.1	3.1
Arg-DCA	549.4011	549.4012	0.18	3.7	3.7
Asn-DCA	507.3429	507.3435	1.18	4.2	4.1
Asp-CA	524.3219	524.3220	0.19	3.6	3.6
Cit-DCA	550.3851	550.3858	1.30	4.2	4.2
Gln-DCA	521.3585	521.3589	0.77	4.2	4.2
Glu-CA	538.3376	538.3379	0.56	3.7	3.7
Glu-DCA	522.3426	522.3428	0.38	4.5	4.5
His-CA	546.3539	546.3542	0.55	3.0	3.0
His-DCA	530.3589	530.3592	0.62	3.6	3.6
Ile/Leu-CA	522.3790	522.3791	0.19	4.7	4.7
Ile/Leu-DCA	506.3840	506.3845	0.99	5.8	5.8
Lys-CA	537.3899	537.3904	0.93	3.4	3.4
Lys-DCA	521.3949	521.3951	0.38	3.8	3.9
Met-DCA	524.3404	524.3406	0.38	5.4	5.4
Phe-CA	556.3634	556.3636	0.36	4.9	4.9
Phe-DCA	540.3684	540.3687	0.56	5.8	5.8
Ser-CA	496.3270	496.3275	1.00	3.6	3.6
Thr-CA	510.3426	510.3425	0.2	3.7	3.7
Thr-DCA	494.3476	494.3482	1.21	4.5	4.5
Tyr-CDCA/ DCA	556.3634	556.3643	1.61	4.9;5.0	4.9; 5.0

Alanine conjugated cholic acid (Ala-CA)

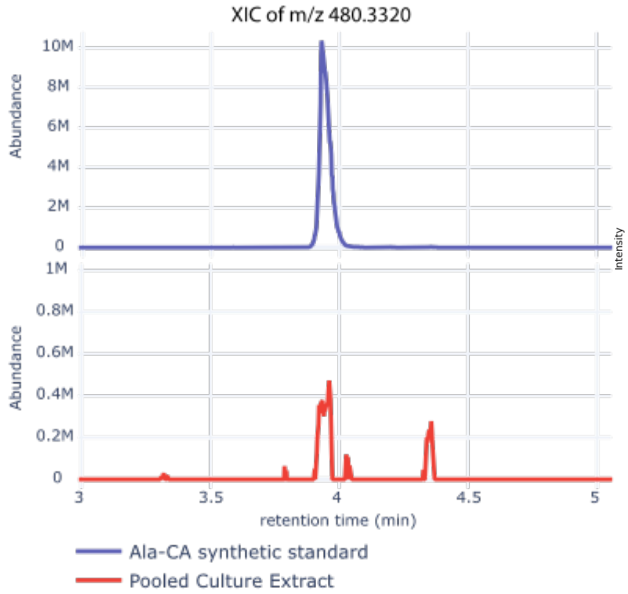

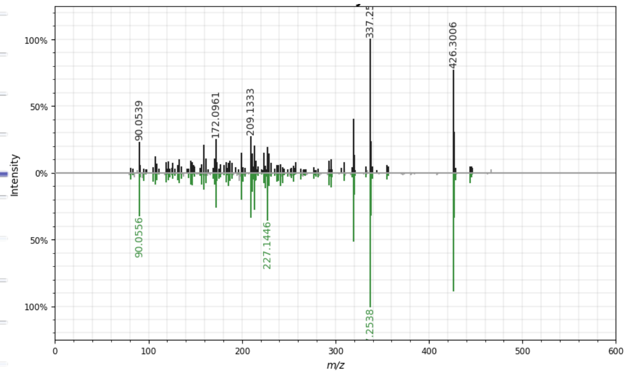

Alanine conjugated deoxycholic acid (Ala-DCA)

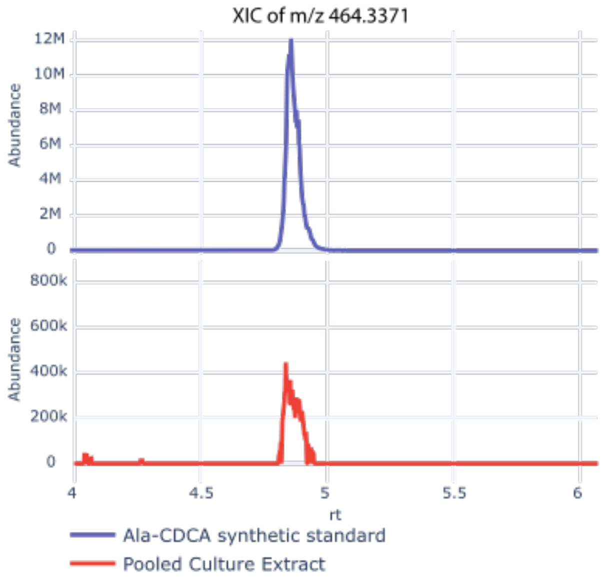

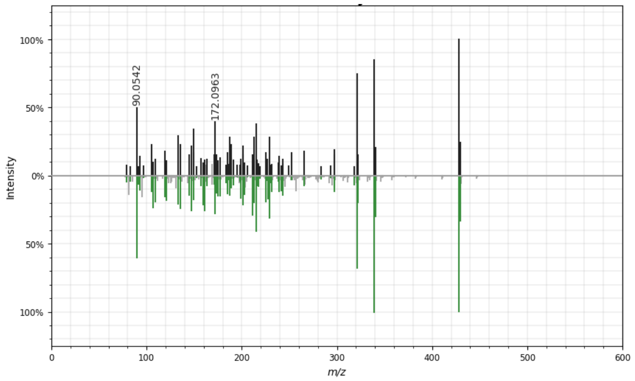

Arginine conjugated cholic acid (Arg-CA)

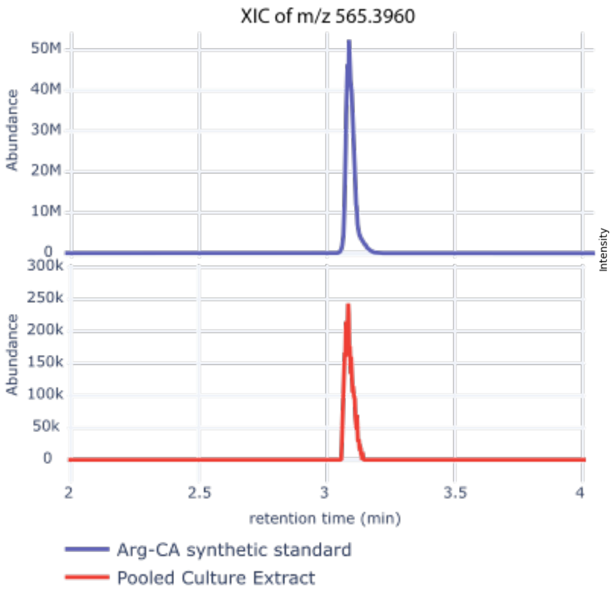

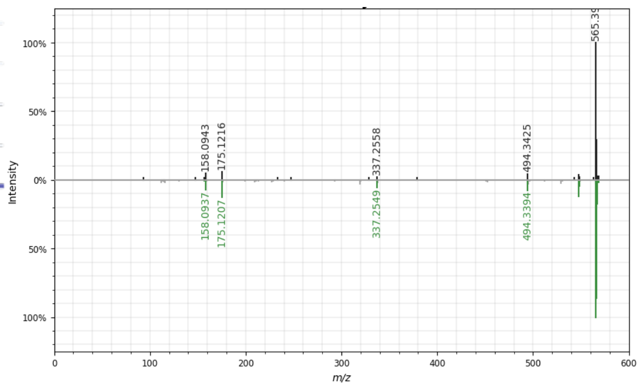

Arginine conjugated deoxycholic acid (Arg–DCA)

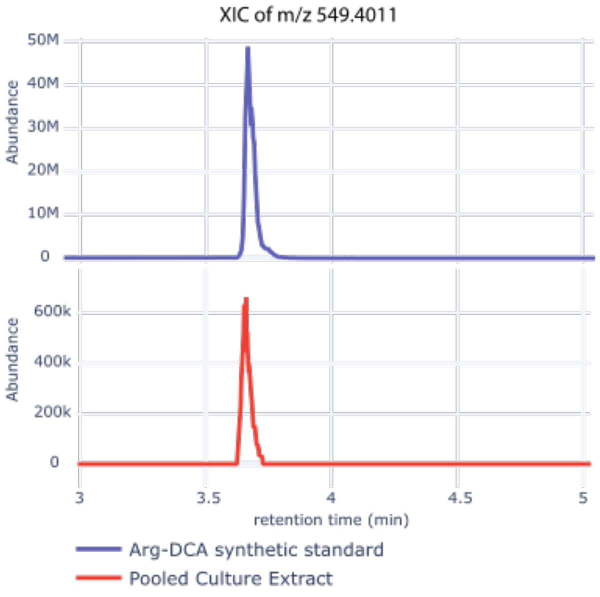

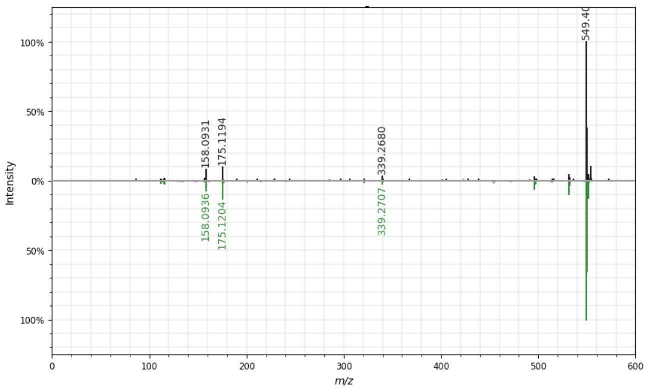

Asparagine conjugated deoxycholic acid (Asn-DCA)

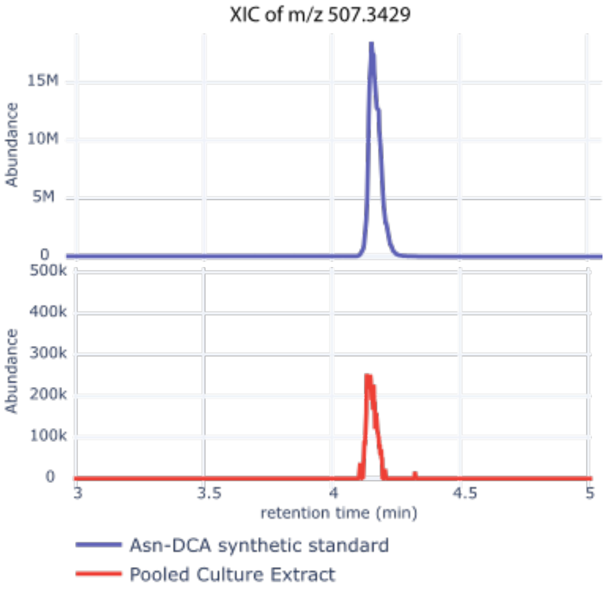

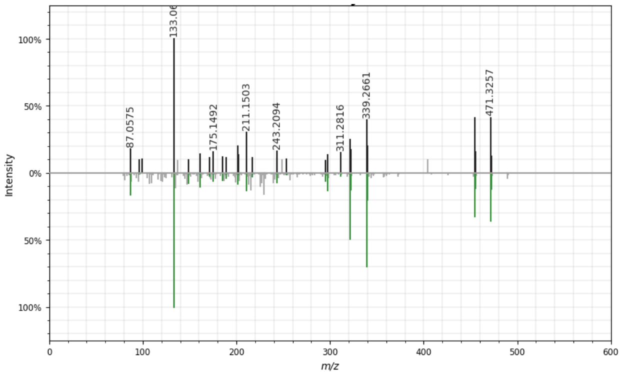

Aspartate conjugated cholic acid (Asp-CA)

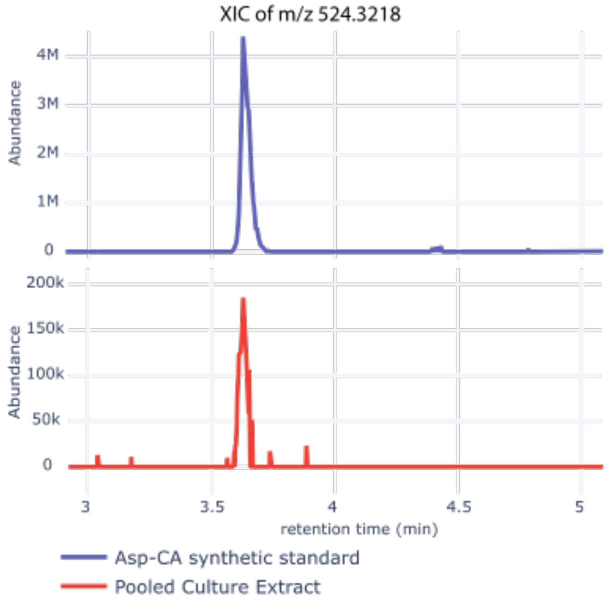

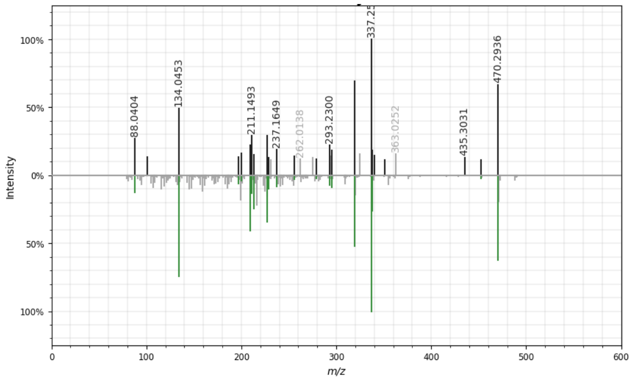

Citrulline conjugated deoxycholic acid (Cit-DCA)

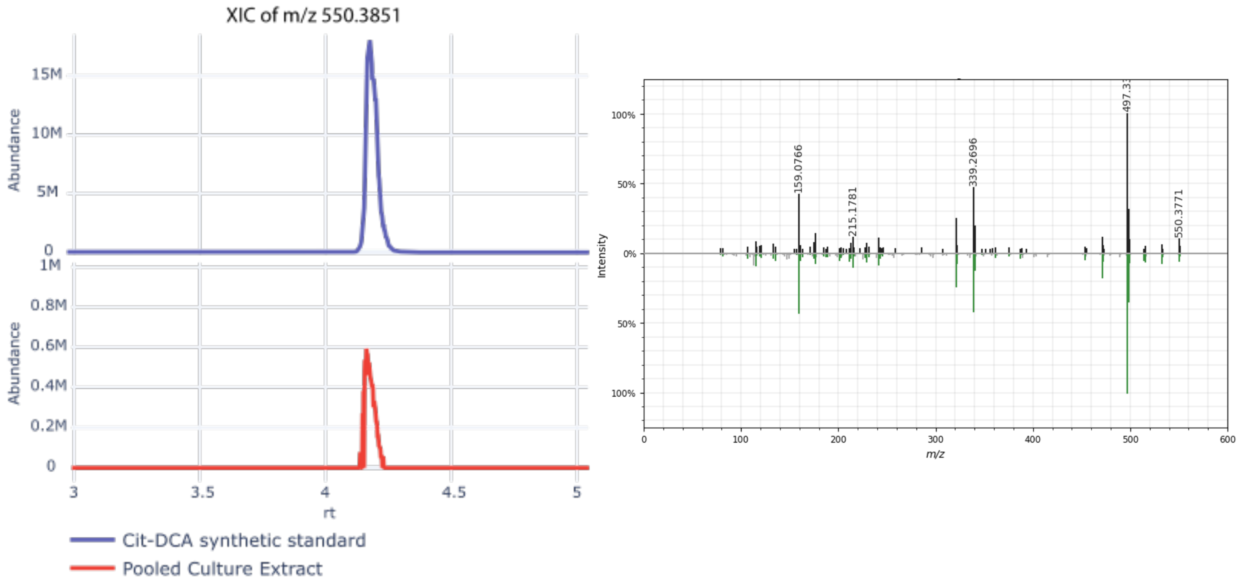

Glutamine conjugated deoxycholic acid (Gln-DCA)

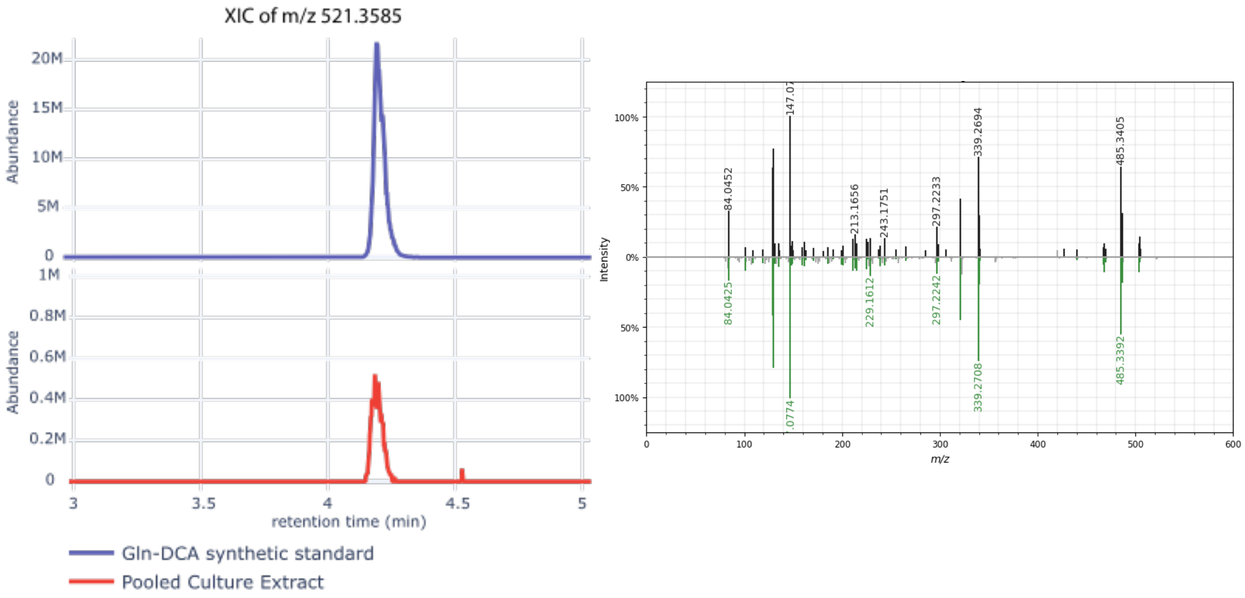

Glutamate conjugated cholic acid (Glu-CA)

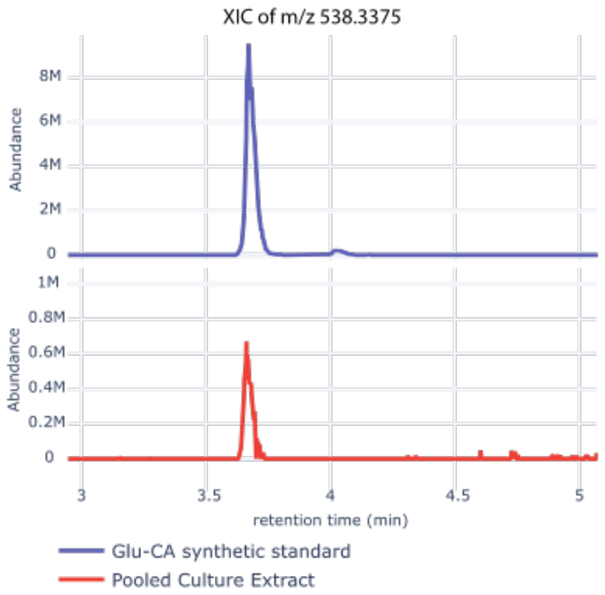

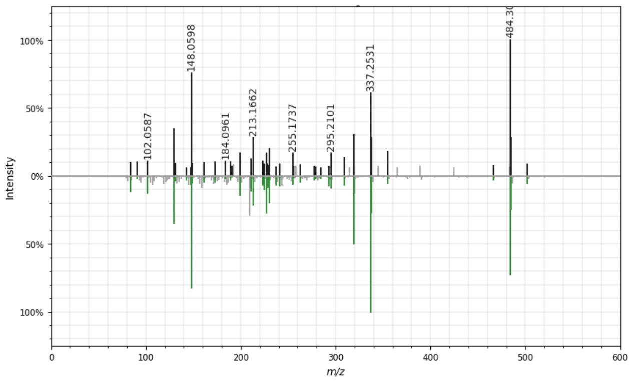

Glutamate conjugated deoxycholic acid (Glu-DCA)

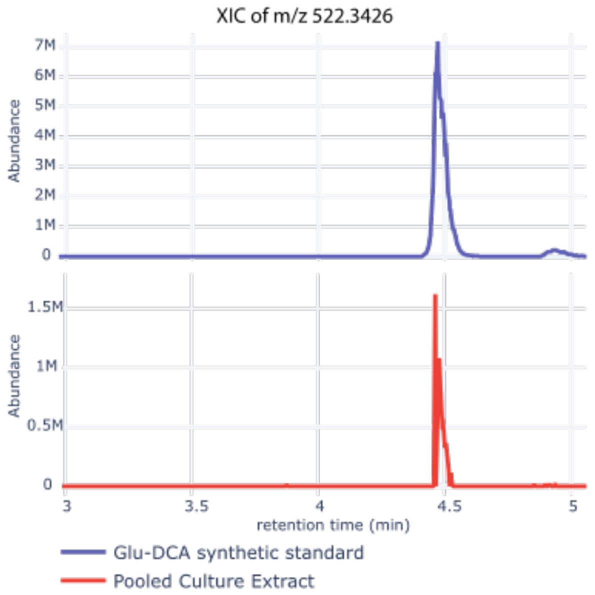

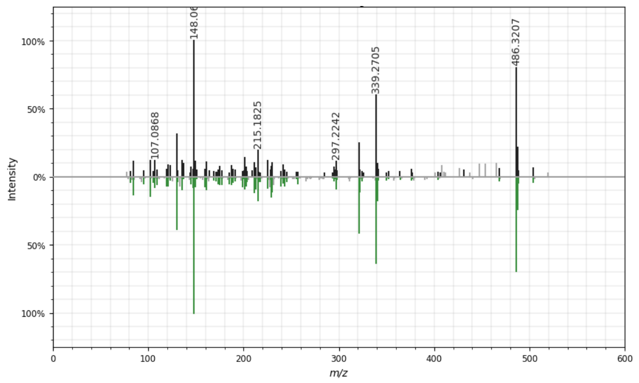

Histidine conjugated cholic acid (His-CA)

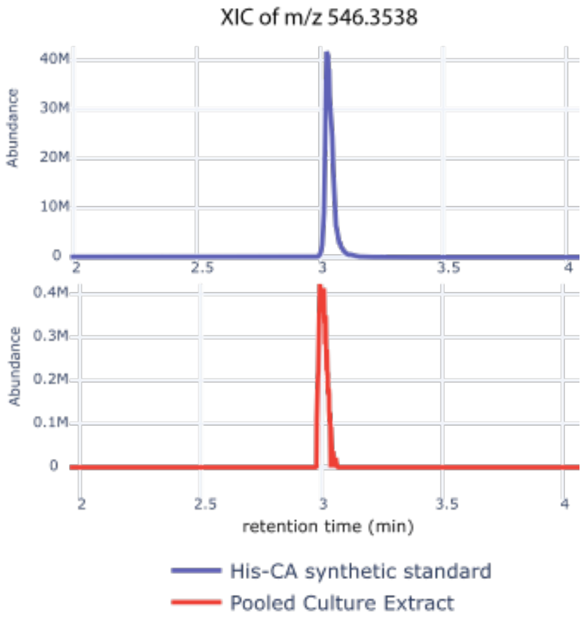

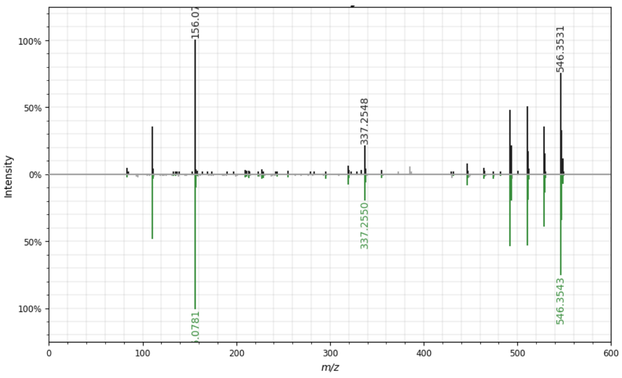

Histidine conjugated deoxycholic acid (His-DCA)

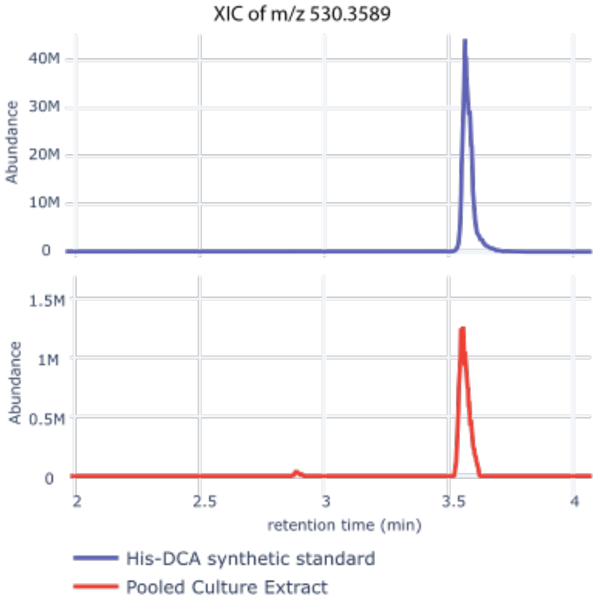

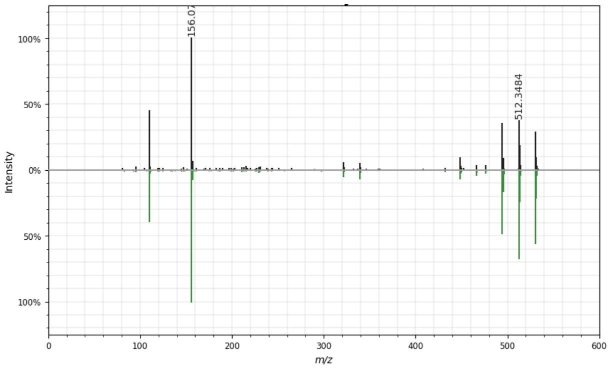

Isoleucine/Leucine conjugated cholic acid (Ile/Leu-CA)

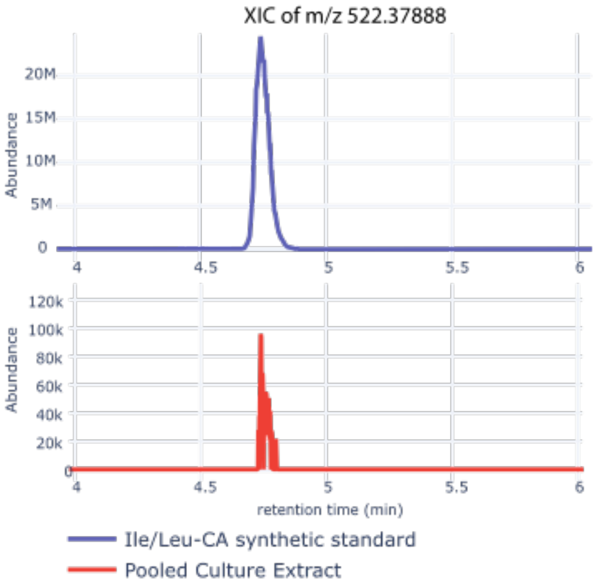

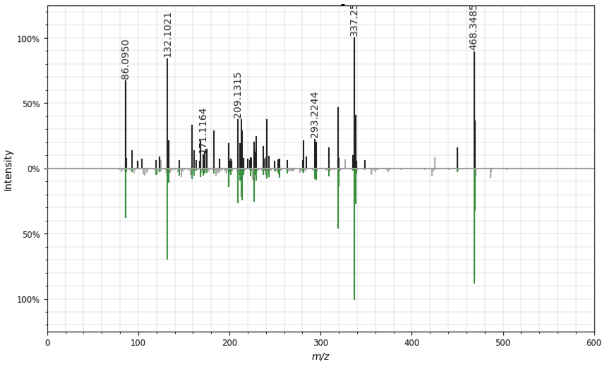

Isoleucine/Leucine conjugated deoxycholic acid (Ile/Leu-DCA)

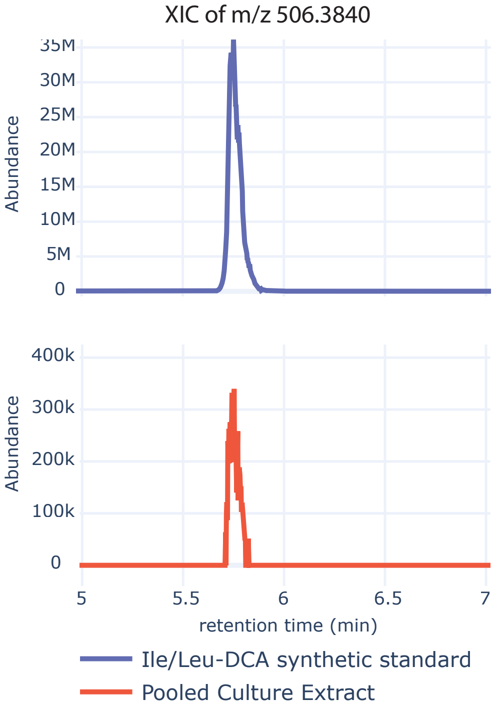

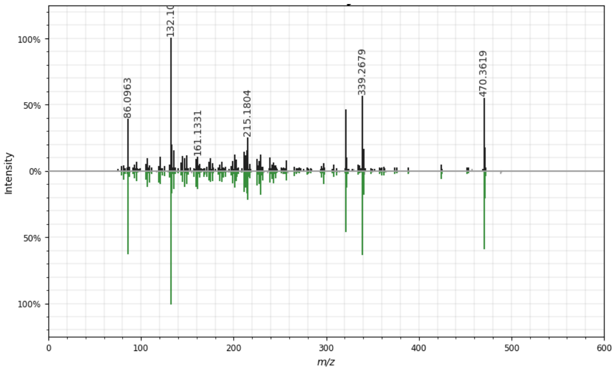

Lysine conjugated cholic acid (Lys-CA)

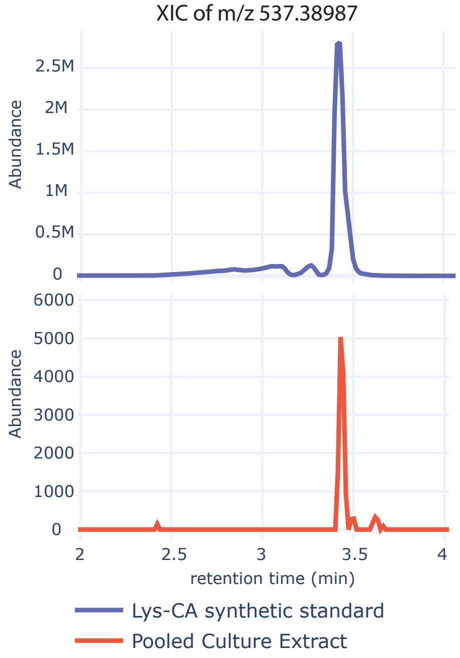

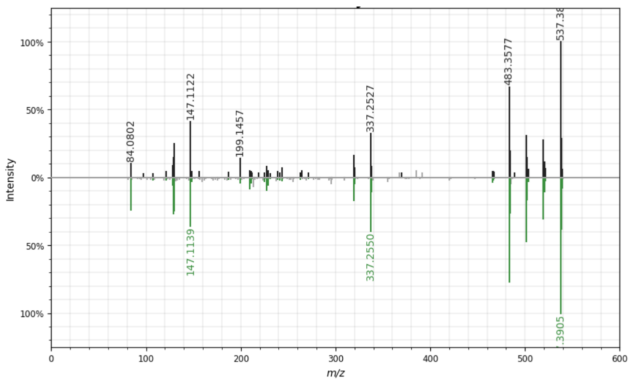

Lysine conjugated deoxycholic acid (Lys-DCA)

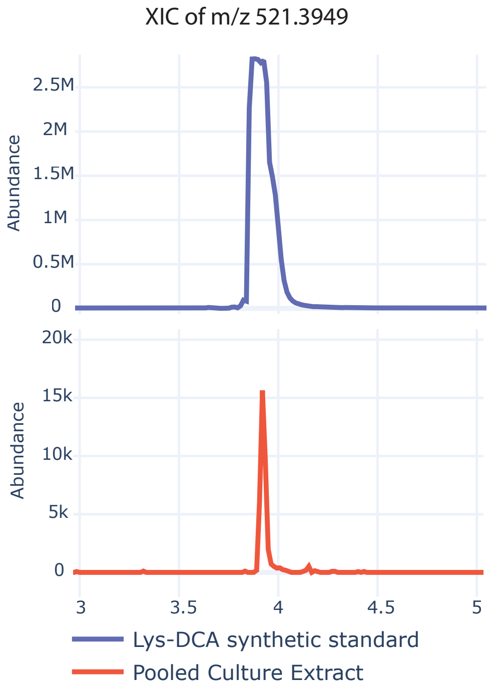

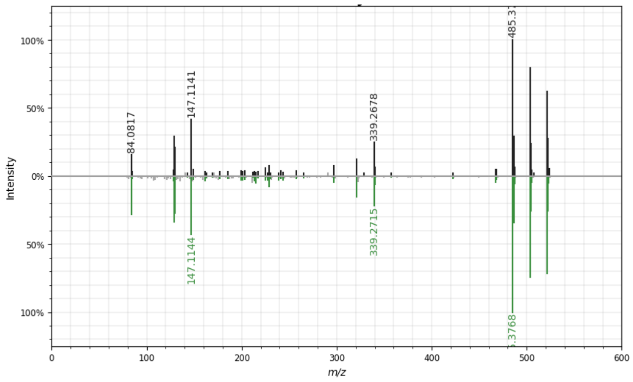

Methionine conjugated deoxycholic acid (Met-DCA)

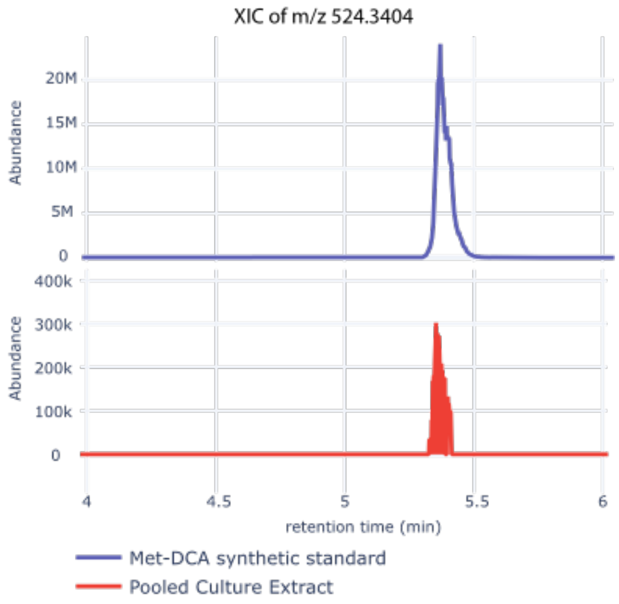

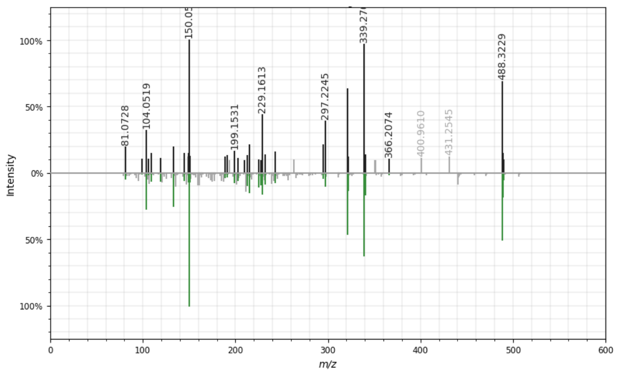

Phenylalanine conjugated cholic acid (Phe-CA)

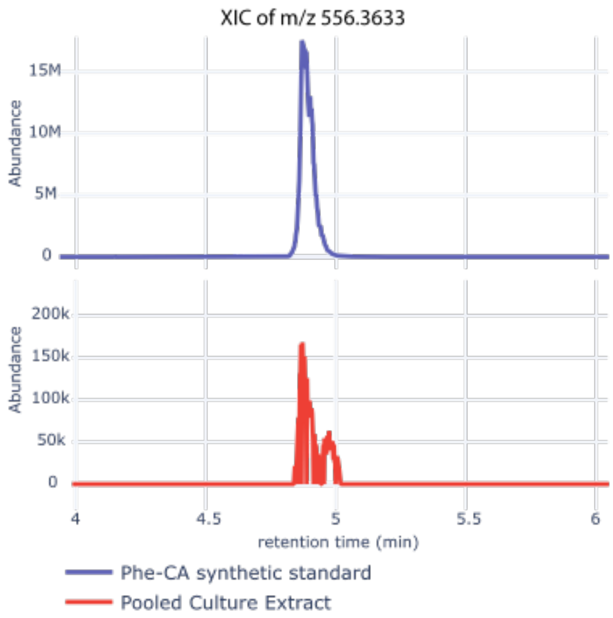

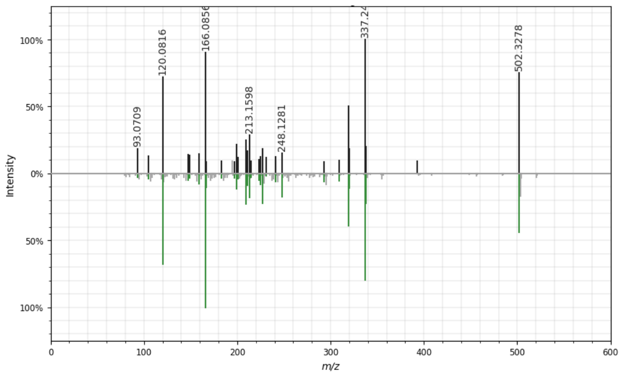

Phenylalanine conjugated deoxycholic acid (Phe-DCA)

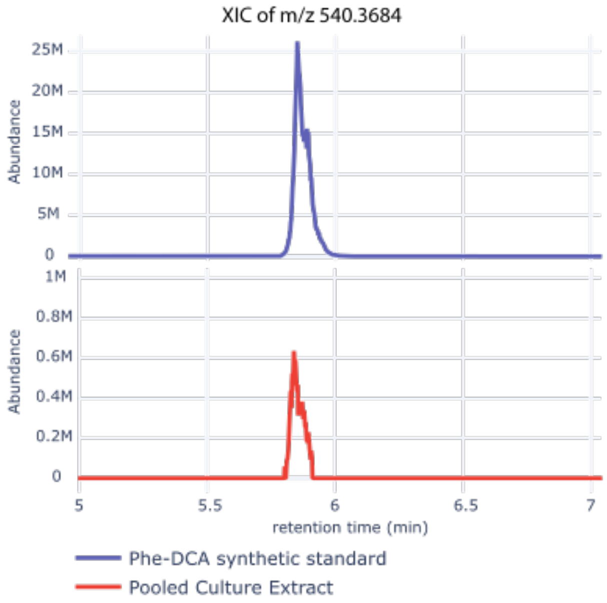

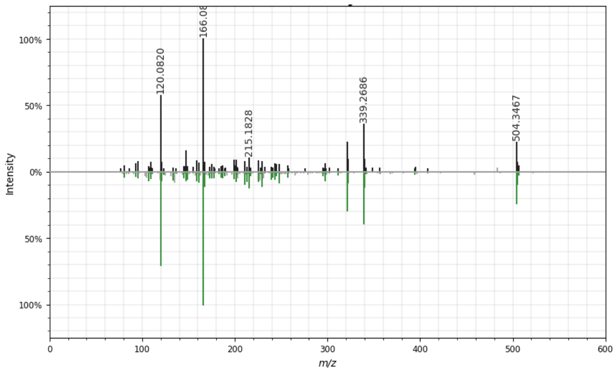

Serine conjugated cholic acid (Ser-CA)

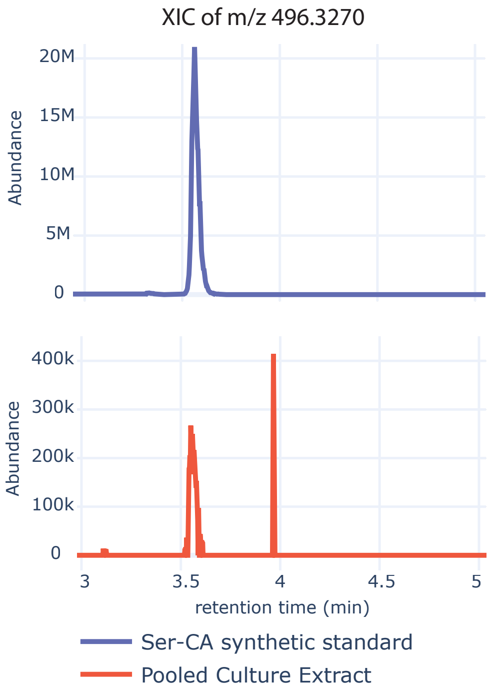

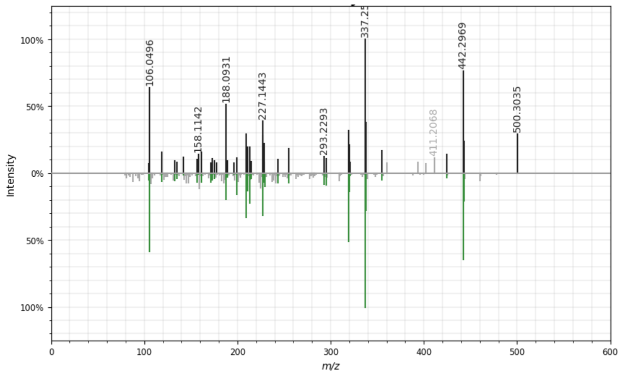

Threonine conjugated cholic acid (Thr-CA)

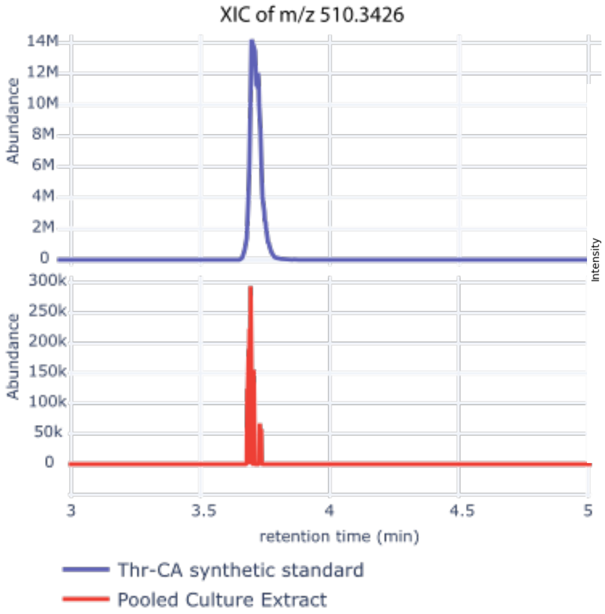

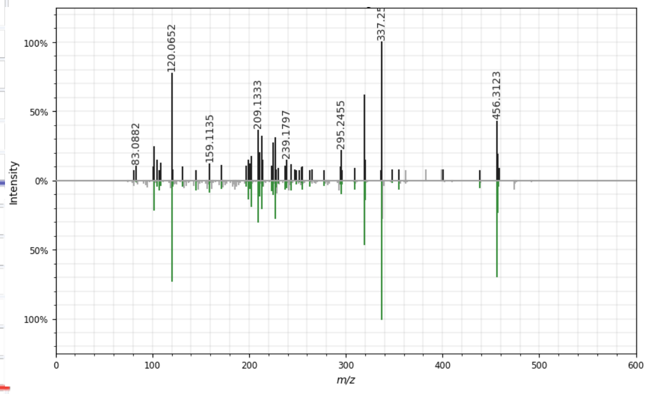

Threonine conjugated deoxycholic acid (Thr-DCA)

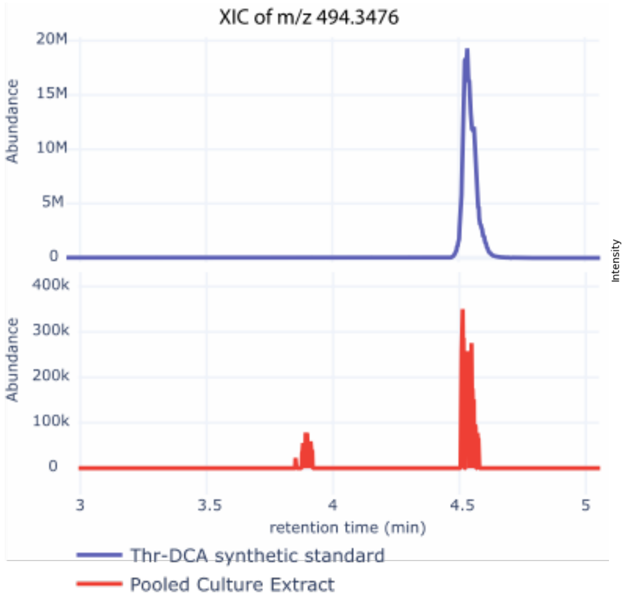

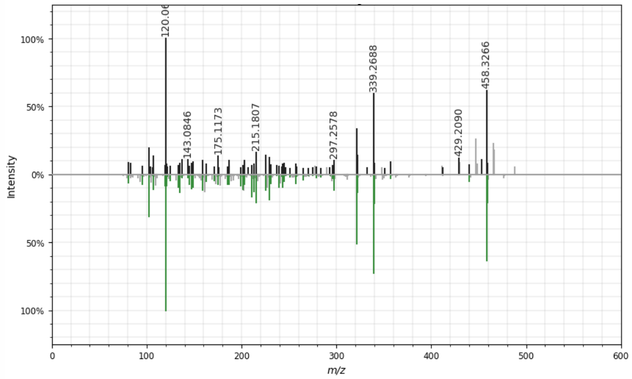

Tyrosine conjugated chenodeoxycholic/deoxycholic acid (Tyr-CDCA/DCA)

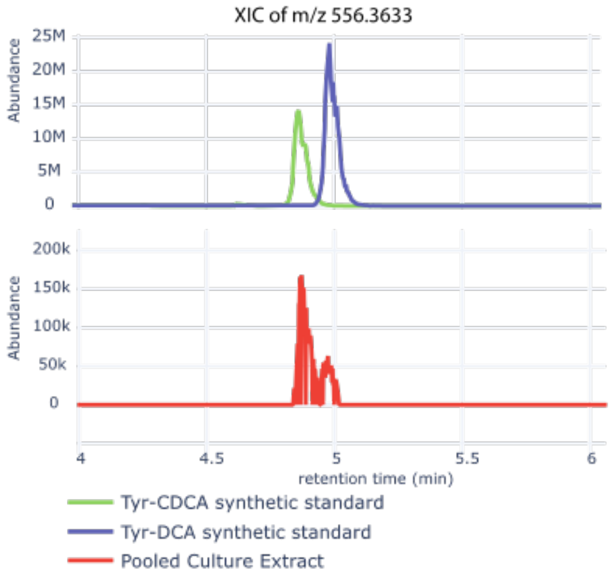

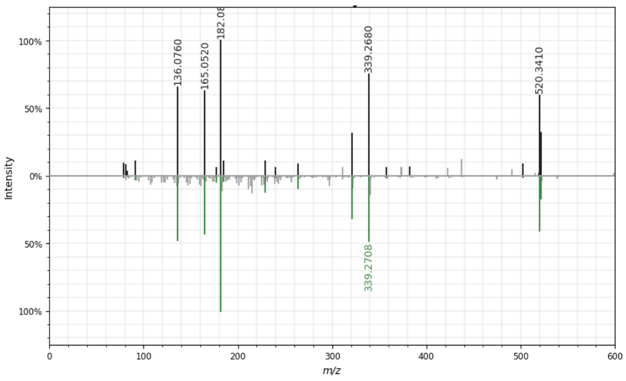

Supplement: TableS8 [file NIHMS1962612-supplement-TableS8.pdf]
