# Supplementary material for: Reverse metabolomics for the discovery of chemical structures from humans
Source: Nature. Author manuscript; Available in PMC 2024 Mar 8. (PMC10849969; doi:10.1038/s41586-023-06906-8)

Conjugated BAs detected in iHMP2 stool samples (negative mode)

Bile Acid	Predicted m/z $[M-H]^-$	Observed m/z $[M-H]^-$	Absolute ppm diff	Standard RT (min)	Sample RT (Min)
Asp-CDCA	506.3123	506.3121	0.40	9.0	9.0
Asp-CA	522.3073	522.3073	0	7.9	7.9
Asp-DCA	506.3123	506.3121	0.40	9.3	9.3
Cit-CDCA	548.3705	548.3706	0.18	8.5	8.5
Cit-CA	564.3654	564.3653	0.18	7.5	7.6
Cit-DCA	548.3705	548.3707	0.36	8.8	8.7
Glu-CDCA	520.3280	520.3278	0.38	9.0	9.0
Glu-CA	536.3230	536.3227	0.56	7.9	7.9
Glu-DCA	520.3280	520.3277	0.58	9.2	9.2
His-CDCA	528.3443	528.3438	0.95	7.3	7.3
His-CA	544.3393	544.3392	0.18	6.6	6.6
His-DCA	528.3443	528.3441	0.38	7.5	7.5
Ile/Leu-CA	520.3644	520.3642	0.38	9.5, 9.6	9.6
Met-CDCA	522.3258	522.3265	1.34	10.3	10.3
Met-CA	538.3208	538.3212	0.74	9.0	9.0
Met-DCA	522.3258	522.3265	1.34	10.6	10.6
Phe-CDCA	538.3538	538.3534	0.74	11.0	11.0
Phe-CA	554.3488	554.3484	0.72	9.8	9.8
Phe-DCA	538.3538	538.3531	1.30	11.3	11.3
Thr-CDCA	492.3330	492.3331	0.20	9.1	9.1
Thr-CA	508.3280	508.3278	0.39	8.0	8.0
Thr-DCA	492.3330	492.3331	0.20	9.4	9.4
Trp-CDCA	577.3647	577.3641	1.04	10.6	10.6
Trp-CA	593.3596	593.3590	1.01	9.5	9.5
Trp-DCA	577.3647	577.3639	1.39	10.9	10.9
Tyr-CDCA	554.3487	554.3480	1.26	9.6	9.6

Bile Acid	Predicted m/z [M+H]+	Observed m/z [M+H]+	Absolute ppm diff	Standard RT (min)	Sample RT (Min)
Tyr-CA	570.3436	570.3433	0.53	8.5	8.5
Tyr-UDCA/ HDCA	554.3487	554.3477	1.80	8.5	8.5
Tyr-UDCA/ HDCA	554.3487	554.3477	1.80	8.5	8.5
Tyr-UDCA/ HDCA	554.3487	554.3477	1.80	8.5	8.5

RT and MS/MS Matching to Synthetic Standards

Aspartate conjugated chenodeoxycholic acid (Asp-CDCA)

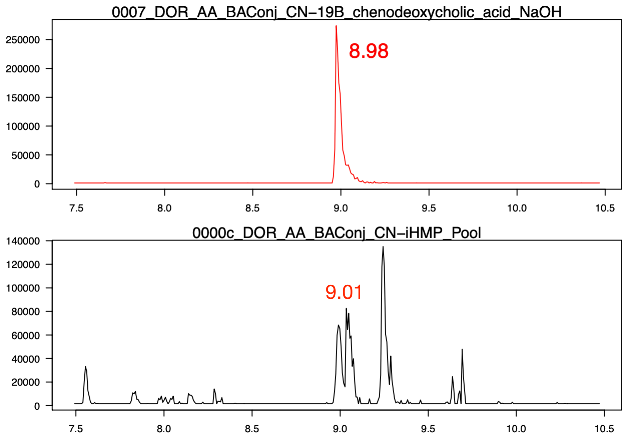

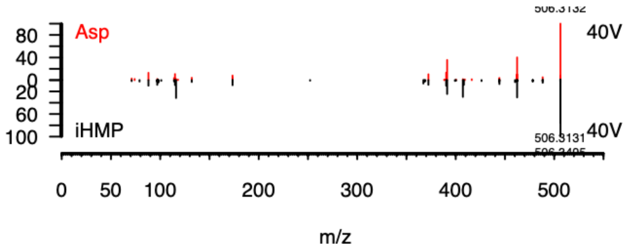

Aspartate conjugated cholic acid (Asp-CA)

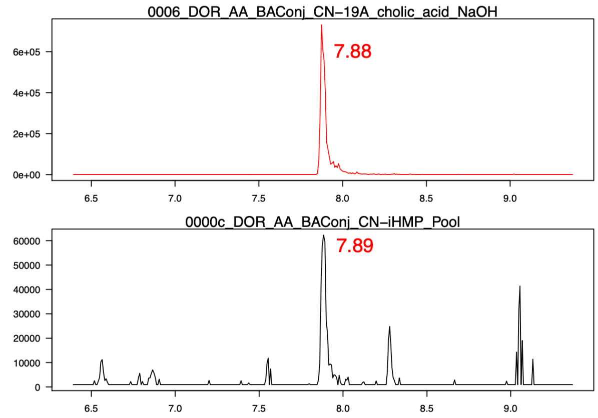

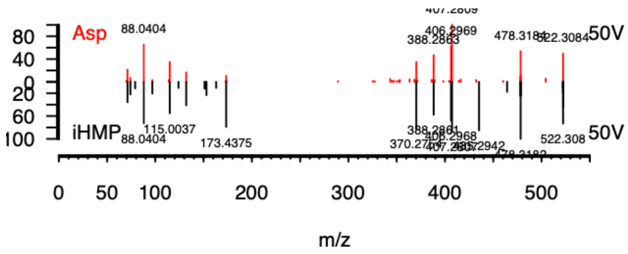

Aspartate conjugated deoxycholic acid (Asp-DCA)

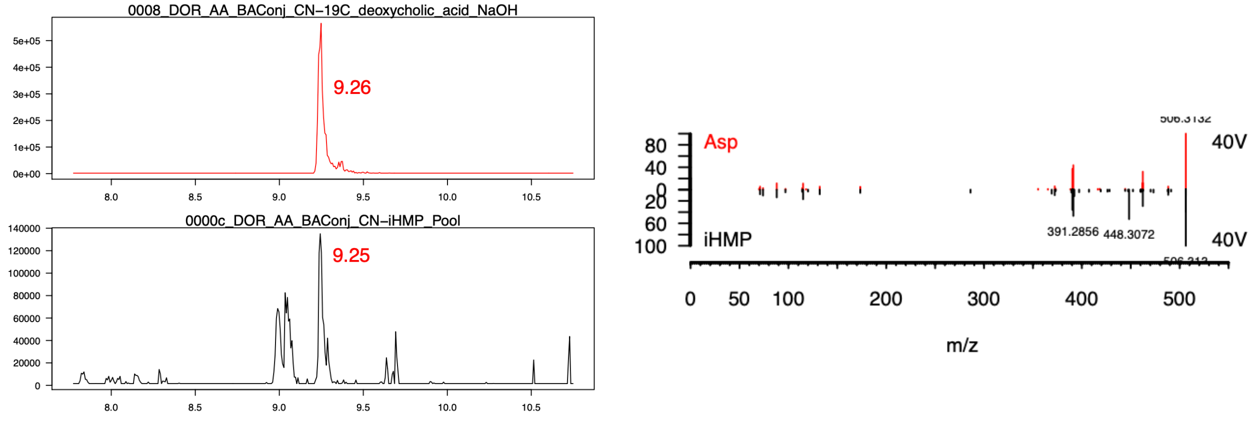

Citrulline conjugated chenodeoxycholic acid (Cit-CDCA)

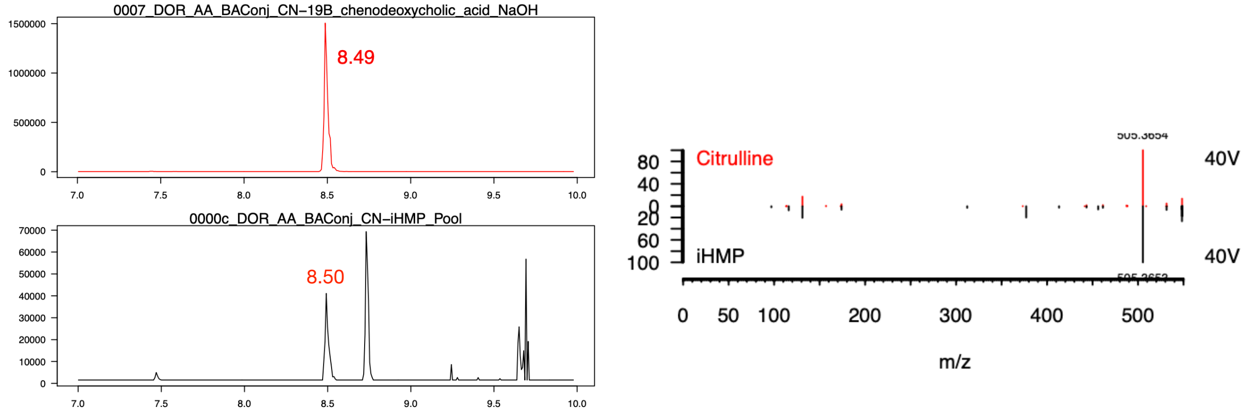

Citrulline conjugated cholic acid (Cit-CA)

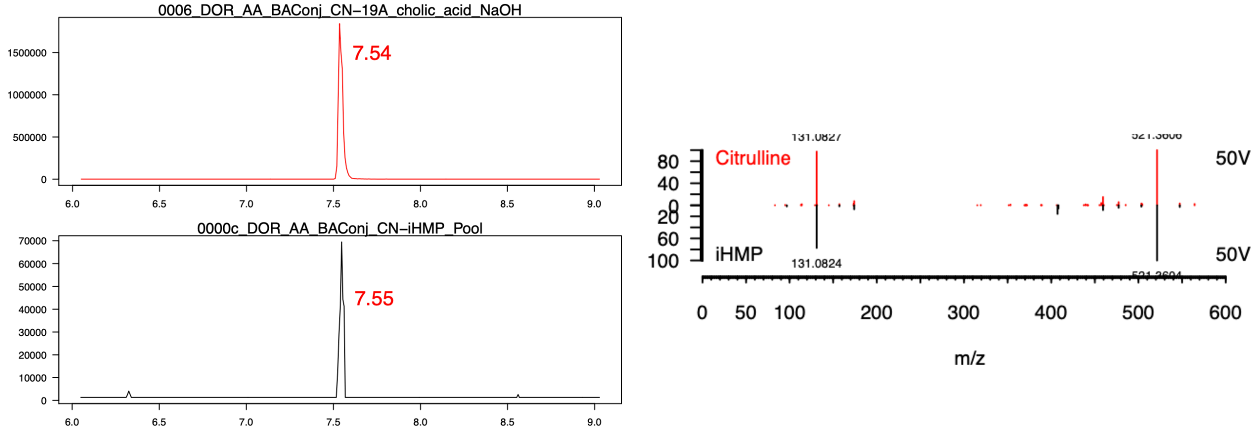

Citrulline conjugated deoxycholic acid (Cit-DCA)

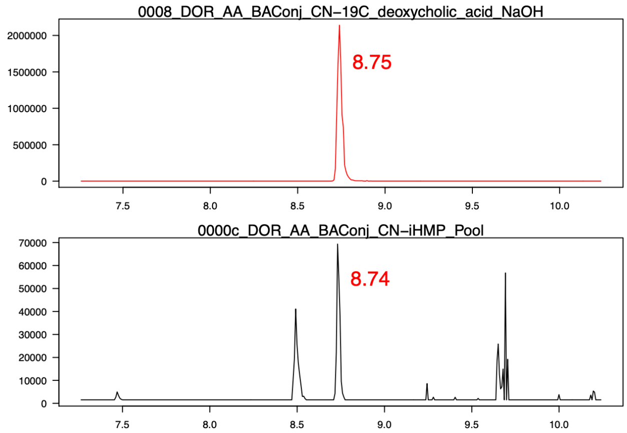

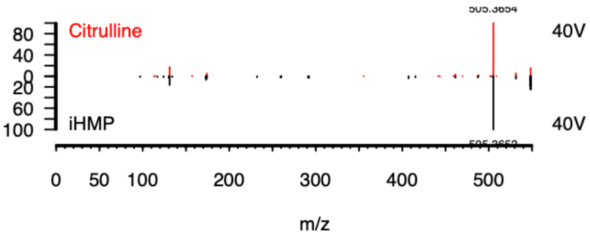

Glutamate conjugated chenodeoxycholic acid (Glu-CDCA)

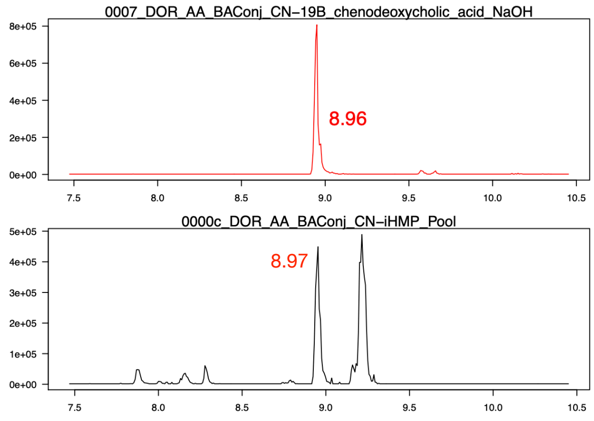

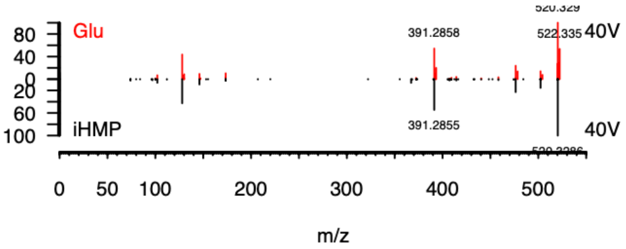

Glutamate conjugated cholic acid (Glu-CA)

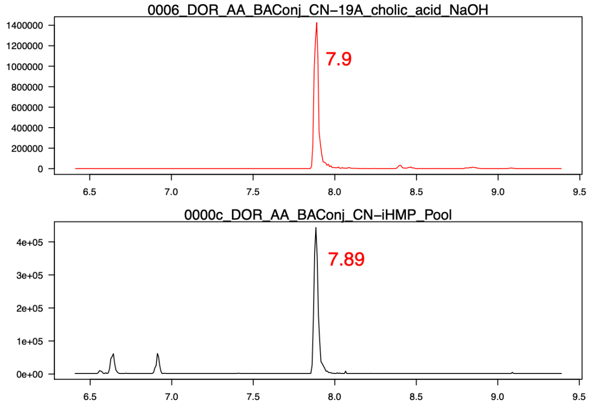

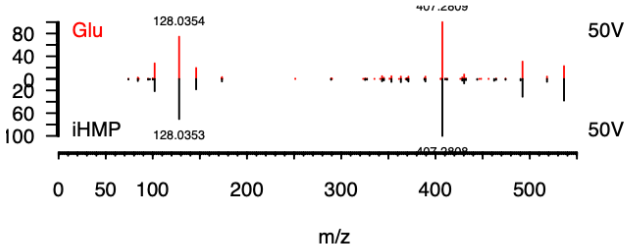

Glutamate conjugated deoxycholic acid (Glu-DCA)

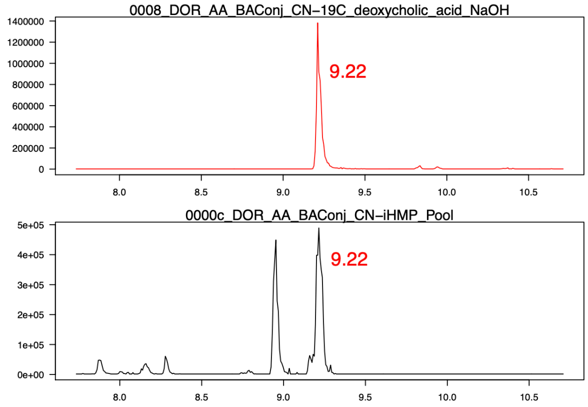

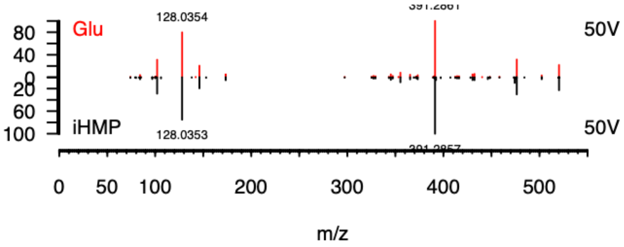

Histidine conjugated chenodeoxycholic acid (His-CDCA)

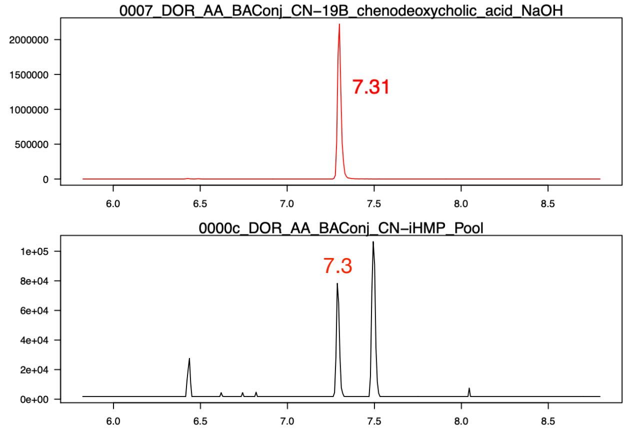

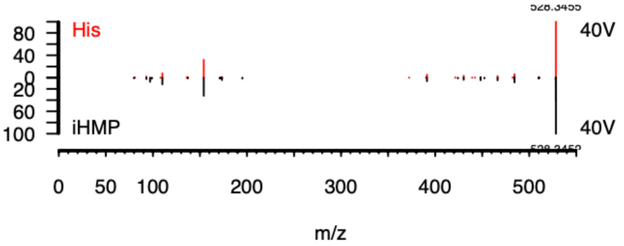

Histidine conjugated cholic acid (His-CA)

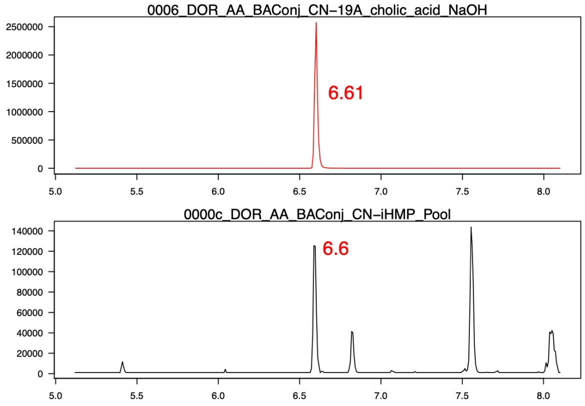

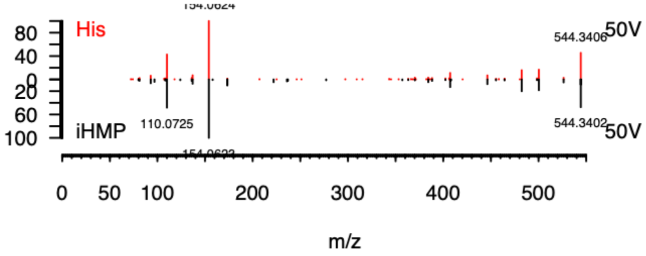

Histidine conjugated deoxycholic acid (His-DCA)

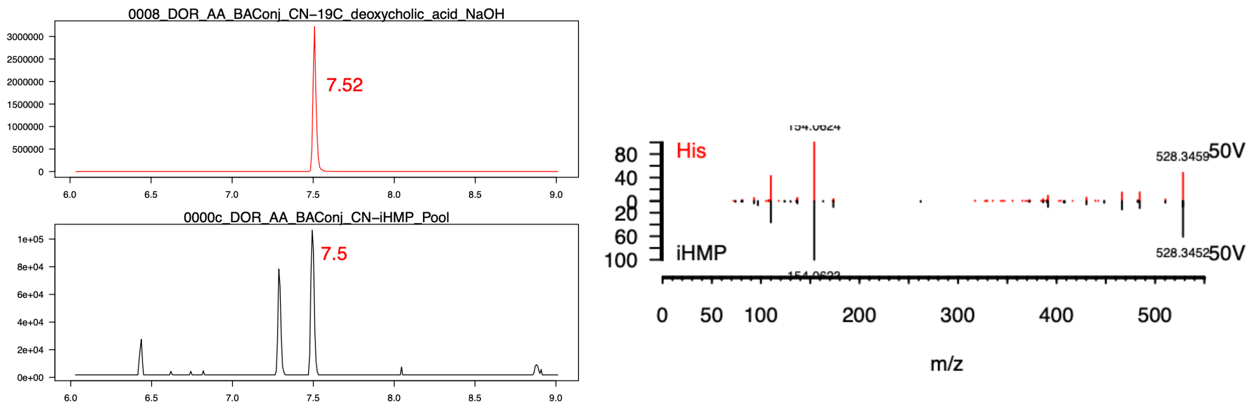

Isoleucine/Leucine conjugated cholic acid (Ile/Leu-CA)

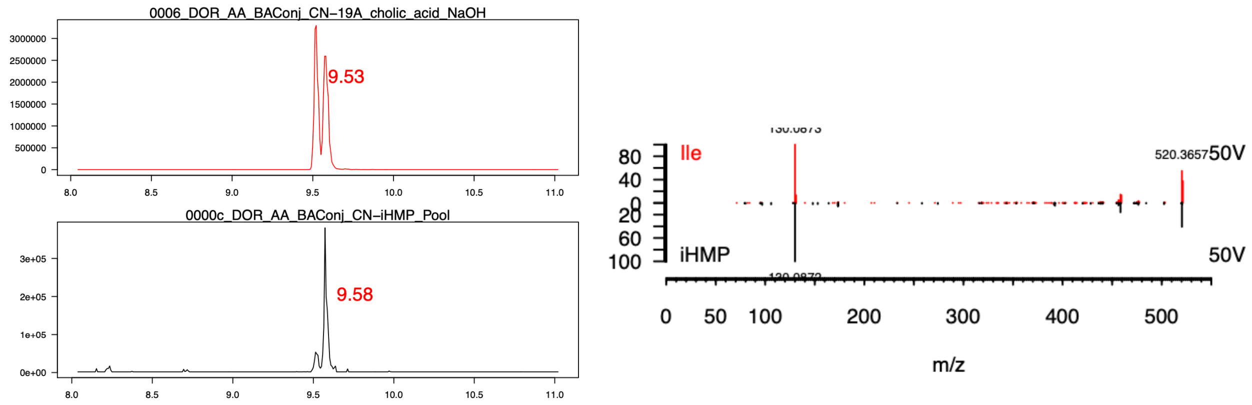

Methionine conjugated chenodeoxycholic acid (Met-CDCA)

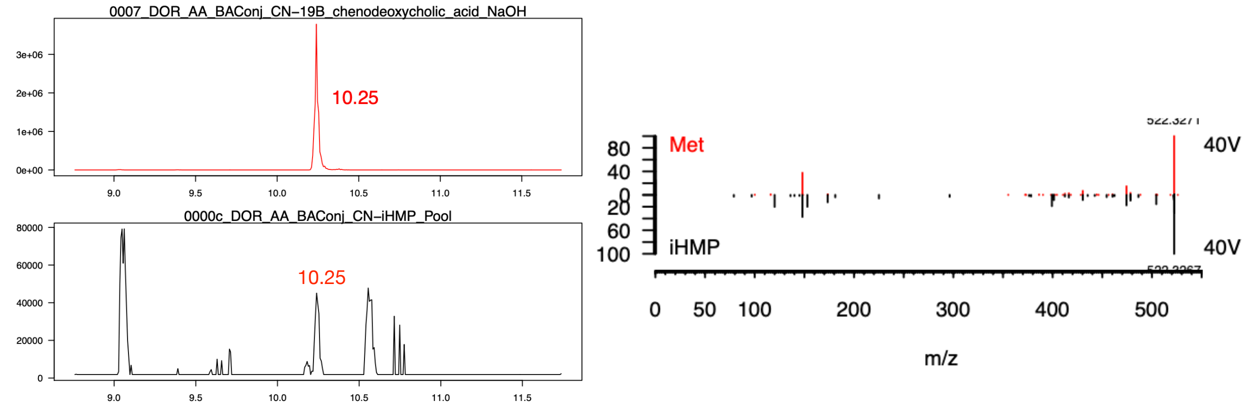

Methionine conjugated cholic acid (Met-CA)

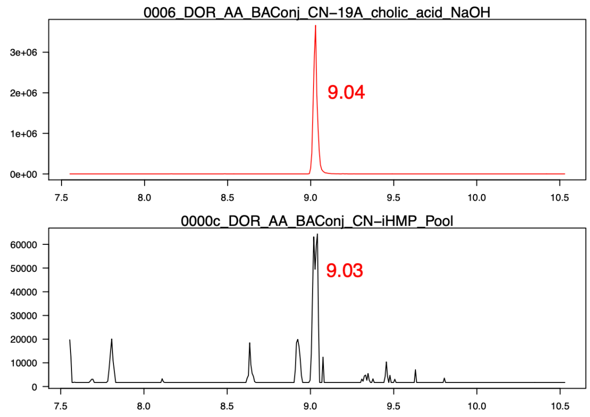

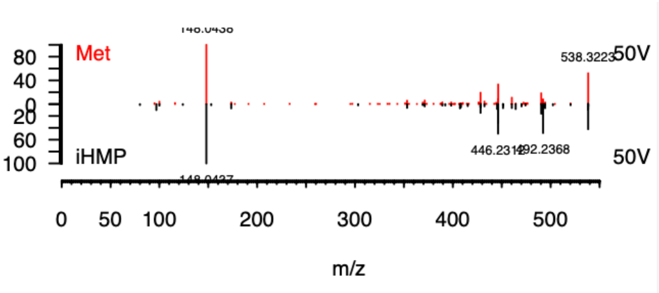

Methionine conjugated deoxycholic acid (Met-DCA)

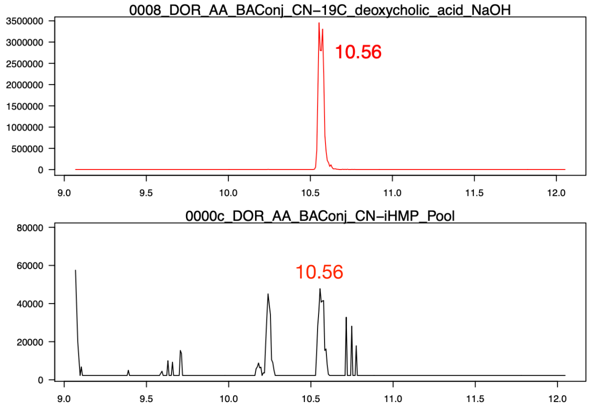

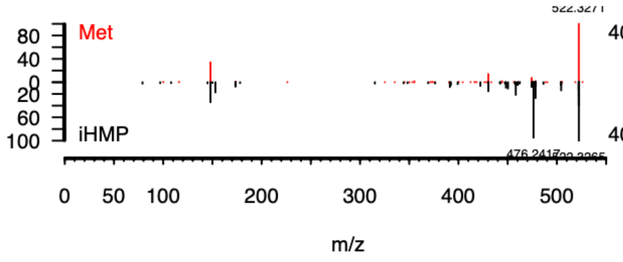

Phenylalanine conjugated chenodeoxycholic acid (Phe-CDCA)

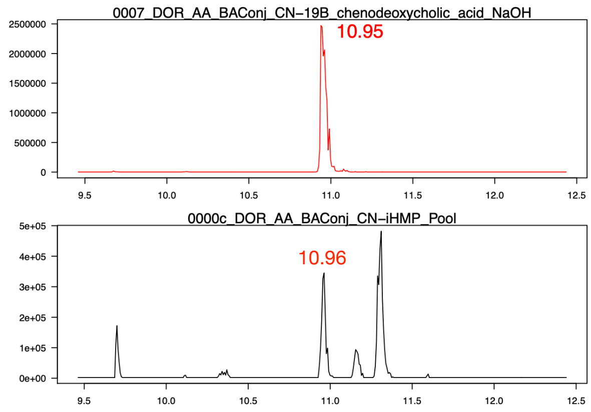

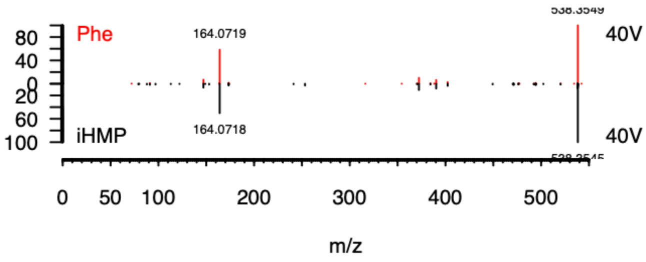

Phenylalanine conjugated cholic acid (Phe-CA)

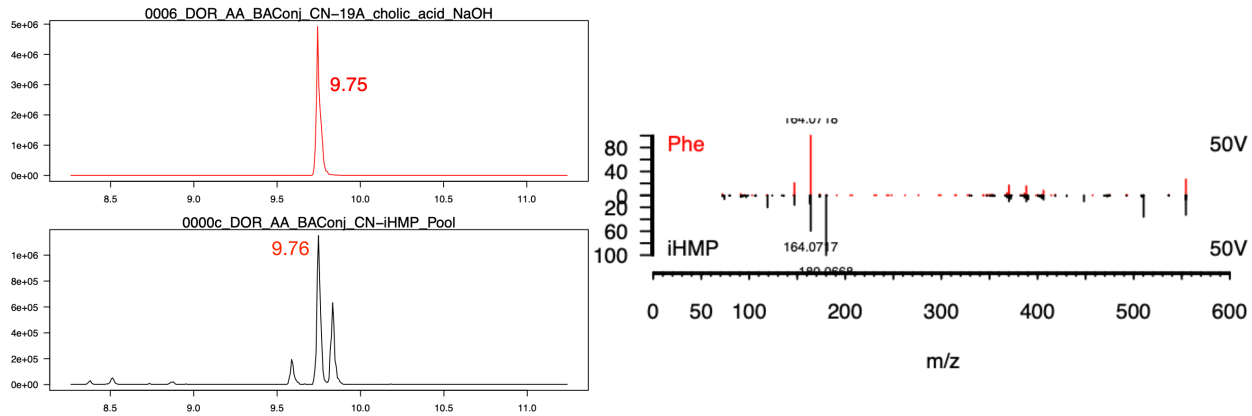

Phenylalanine conjugated deoxycholic acid (Phe-DCA)

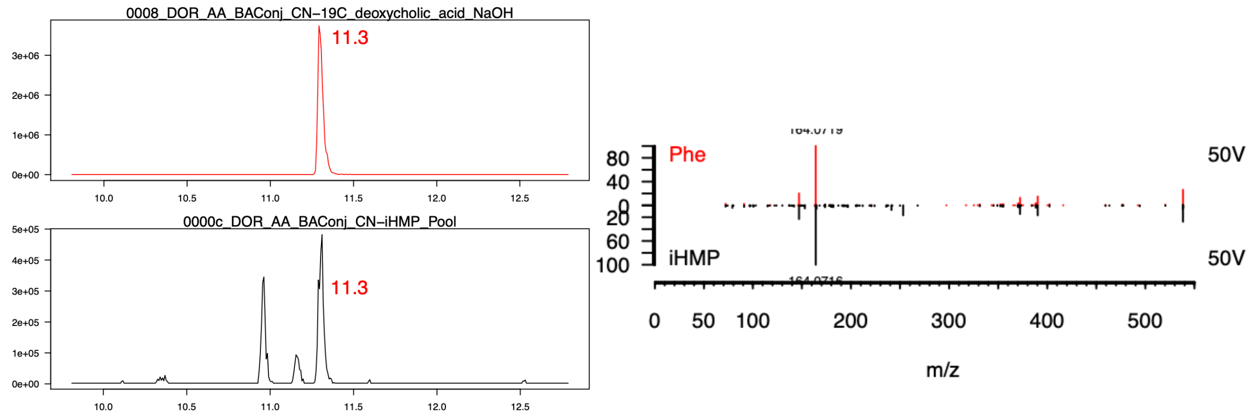

Threonine conjugated chenodeoxycholic acid (Thr-CDCA)

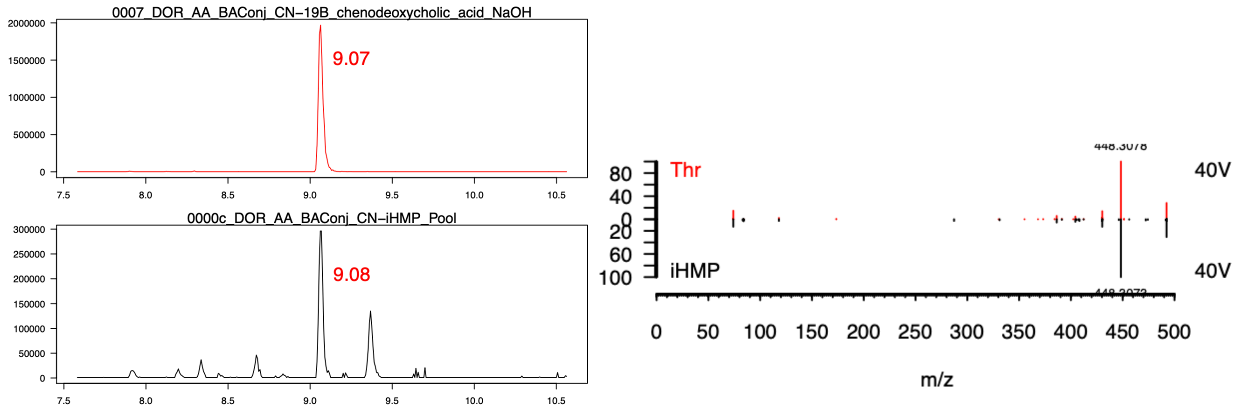

Threonine conjugated cholic acid (Thr-CA)

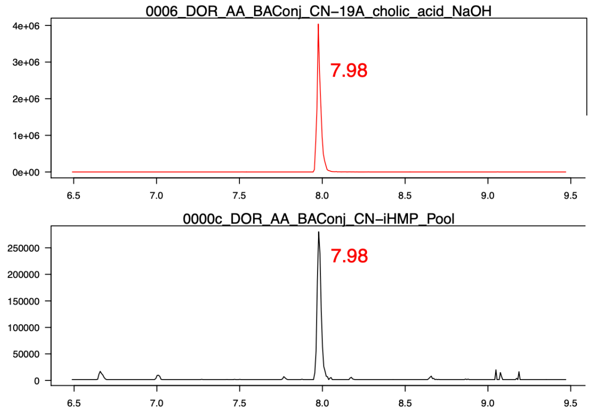

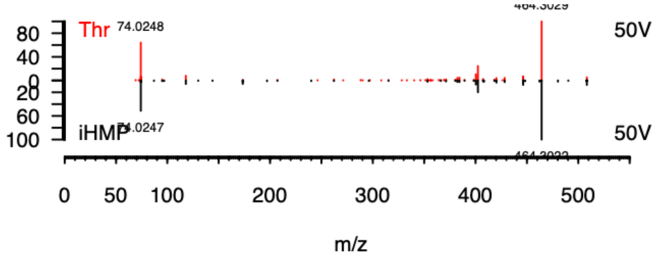

Threonine conjugated deoxycholic acid (Thr-DCA)

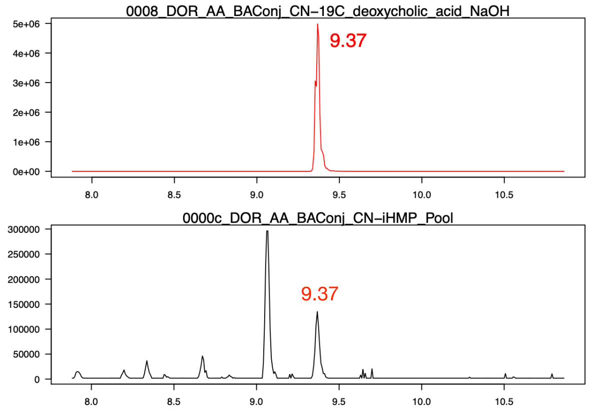

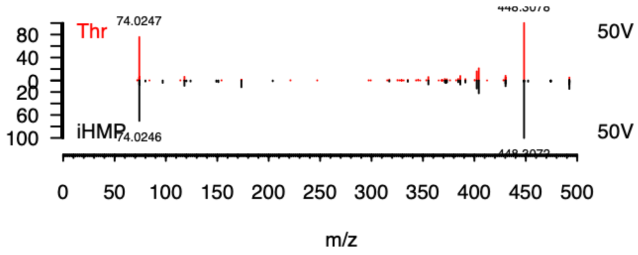

Tryptophan conjugated chenodeoxycholic acid (Trp-CDCA)

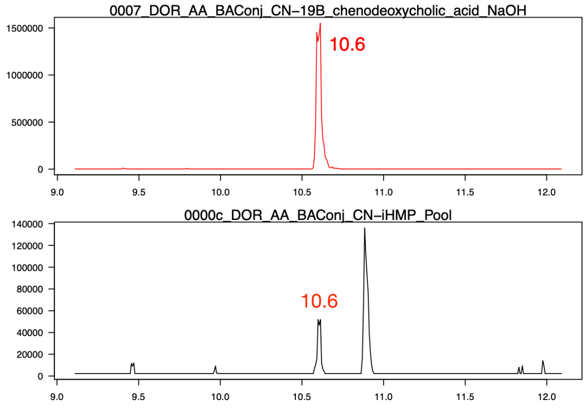

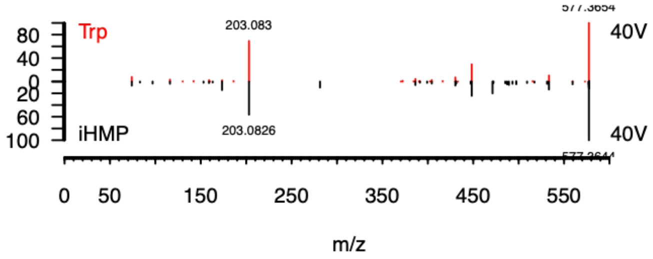

Tryptophan conjugated cholic acid (Trp-CA)

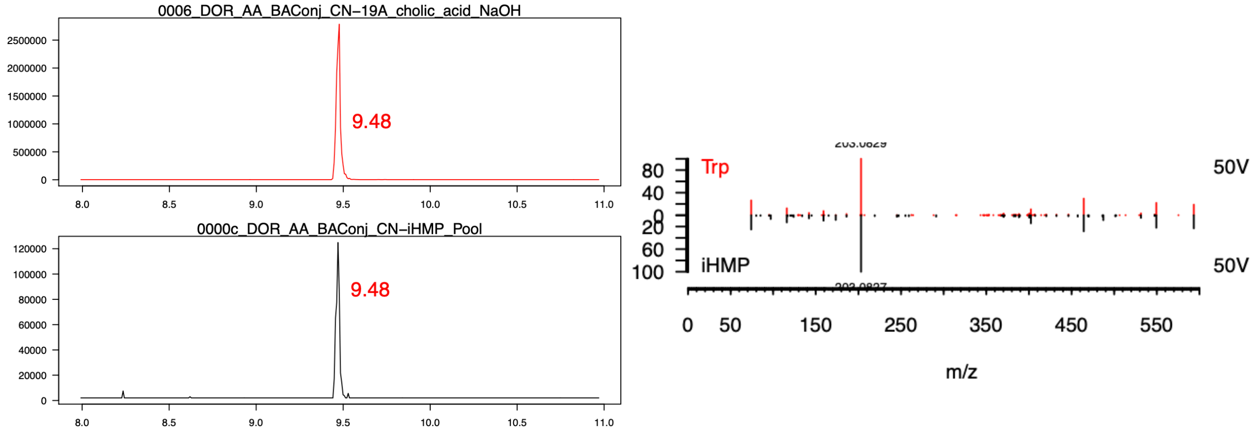

Tryptophan conjugated deoxycholic acid (Trp-DCA)

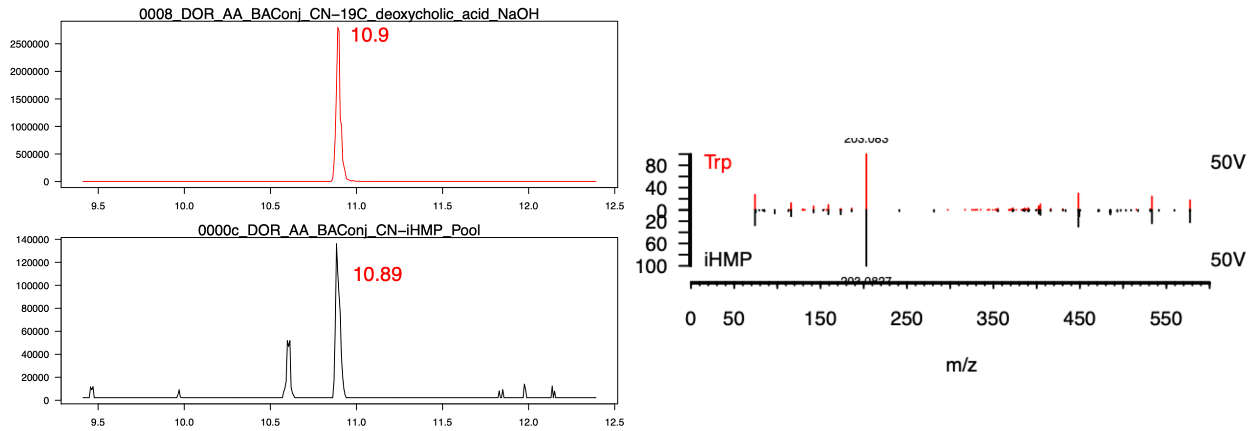

Tyrosine conjugated chenodeoxycholic acid (Tyr-CDCA)

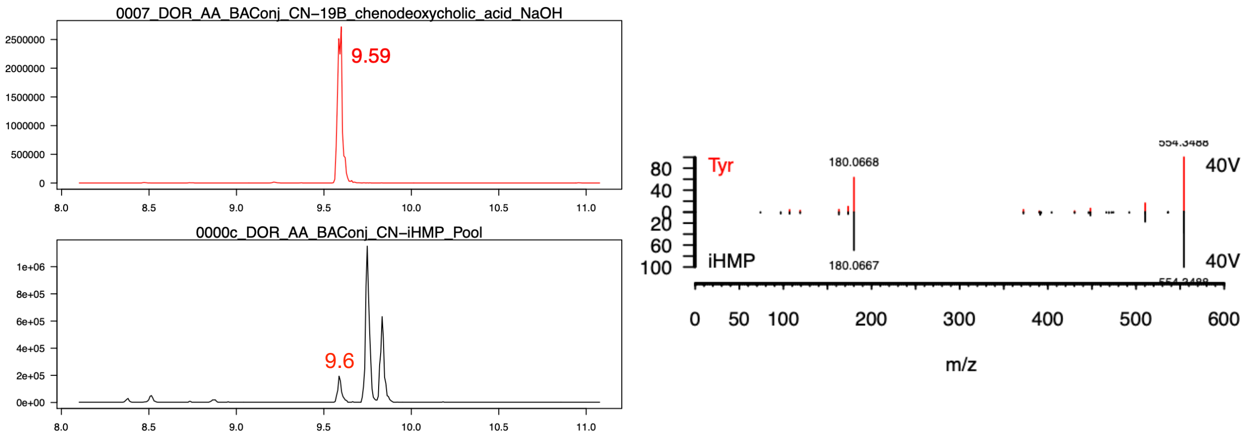

Tyrosine conjugated cholic acid (Tyr-CA)

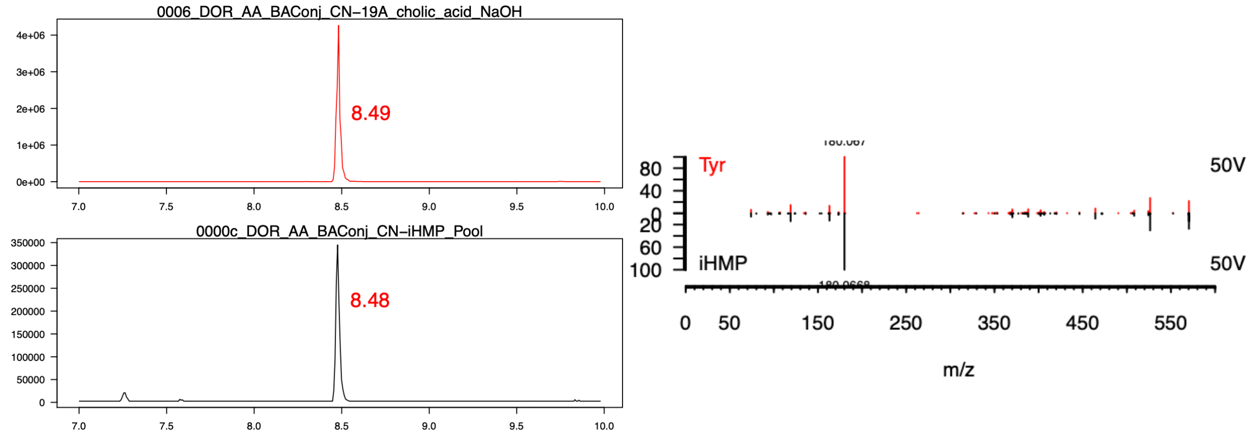

Tyrosine conjugated hyodeoxycholic acid/ursodeoxycholic acid (Tyr-HDCA/UDCA)

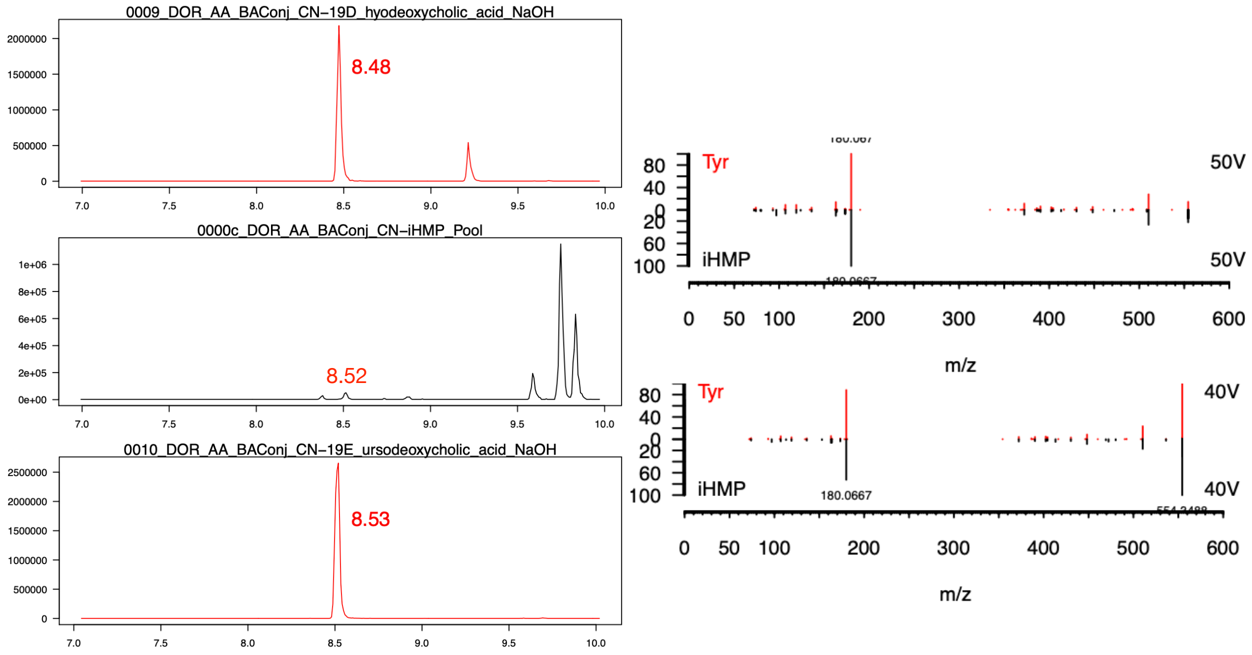

Supplement: TableS6 [file NIHMS1962612-supplement-TableS6.pdf]
