# Supplementary material for: Reverse metabolomics for the discovery of chemical structures from humans
Source: Nature. Author manuscript; Available in PMC 2024 Mar 8. (PMC10849969; doi:10.1038/s41586-023-06906-8)
Supplement: TableS4 [file NIHMS1962612-supplement-TableS4.pdf]

Isolation mass value (m/z)	Charge state value (z)	Isolation width value (m/z)	Collision energy value (eV)
100	1	4	22
300	1	5	27
500	1	6	35
1000	1	8	45
2000	1	10	50
100	2	4	18
300	2	5	22
500	2	6	30
1000	2	8	35
2000	2	10	50
