# Supplementary material for: Reverse metabolomics for the discovery of chemical structures from humans
Source: Nature. Author manuscript; Available in PMC 2024 Mar 8. (PMC10849969; doi:10.1038/s41586-023-06906-8)

Conjugated bile acids detected in human IBD fecal samples

Bile Acid	predicted m/z $[M+H]^+$	observed m/z $[M+H]^+$	Absolute ppm diff	Standard RT (min)	Sample RT (min)
Glu-CA	538.3376	538.3376	0	4.0	4.0
Glu-CDCA	522.3426	522.3427	0.19	4.6	4.6
Glu-DCA	522.3426	522.3424	0.38	4.7	4.7
Ile/Leu-CA	522.3790	522.3788	0.38	5.0	5.0
Met-CDCA	524.3404	524.3406	0.38	5.5	5.5
Met-DCA	524.3404	524.3407	0.57	5.6	5.6
Phe-CA/Tyr-CDCA	556.3633	556.3633	0	5.1	5.1
Phe-CDCA	540.3684	540.3687	0.56	5.9	5.9
Phe-DCA	540.3684	540.3683	0.19	6.1	6.1
Thr-CA	510.3426	510.3427	0.20	4.0	4.0
Trp-CA	595.3743	595.3747	0.67	5.0	5.0
Trp-CDCA	579.3793	579.3794	0.17	5.8	5.8
Tyr-CA	572.3583	572.3581	0.35	4.4	4.4

RT and MS/MS Matching to Synthetic Standards

Glutamate conjugated cholic acid (Glu-CA)

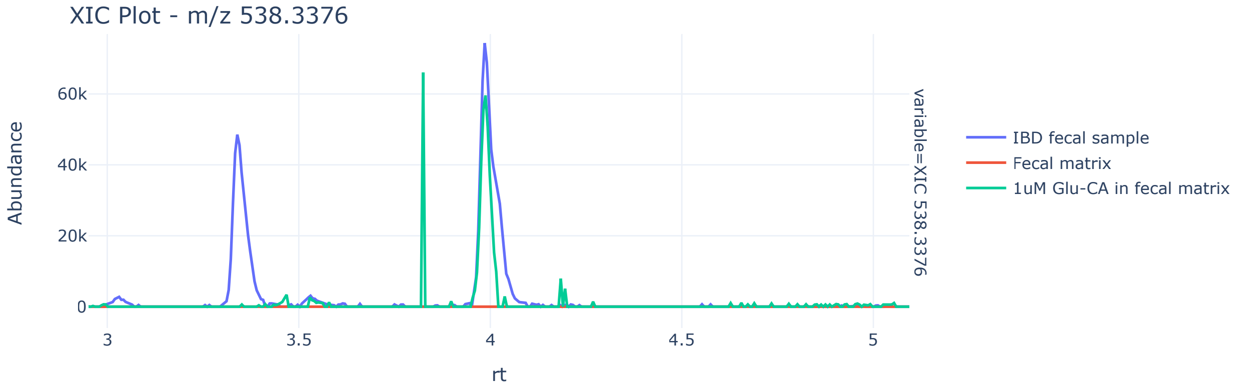

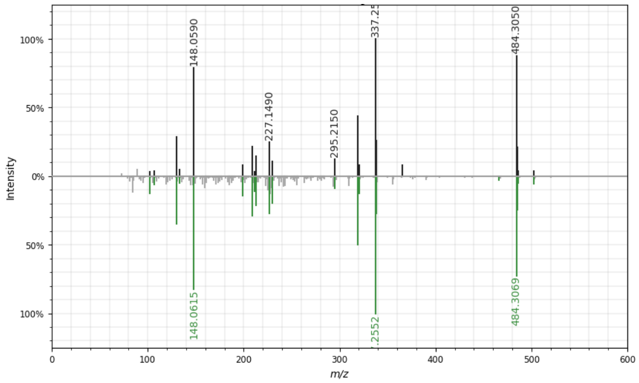

Glutamate conjugated chenodeoxycholic acid (Glu-CDCA)

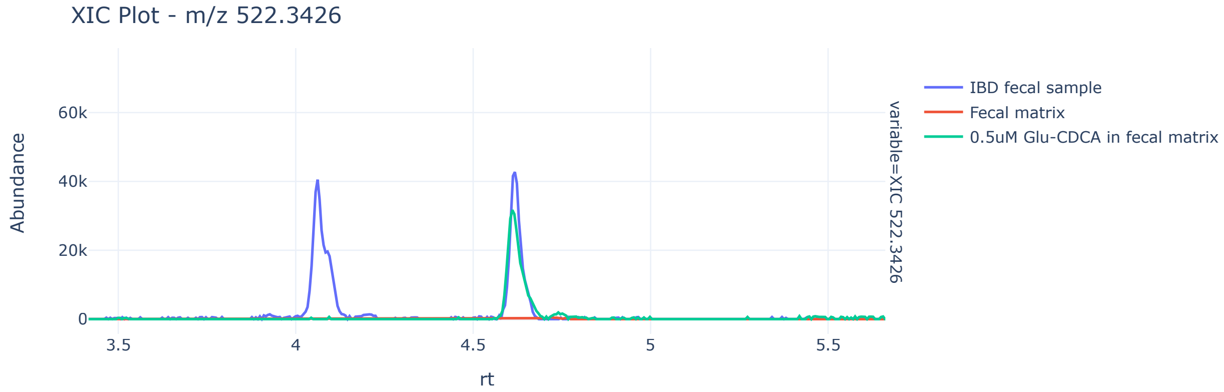

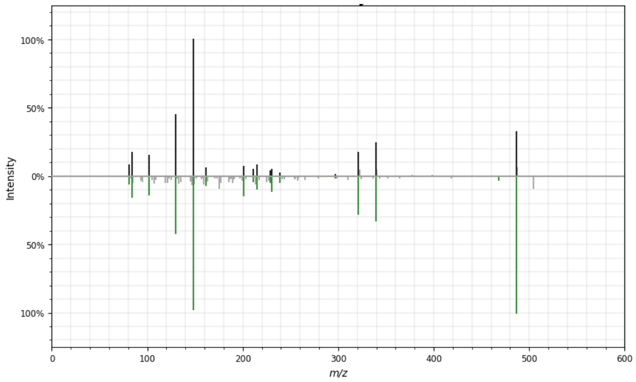

Glutamate conjugated deoxycholic acid (Glu-DCA)

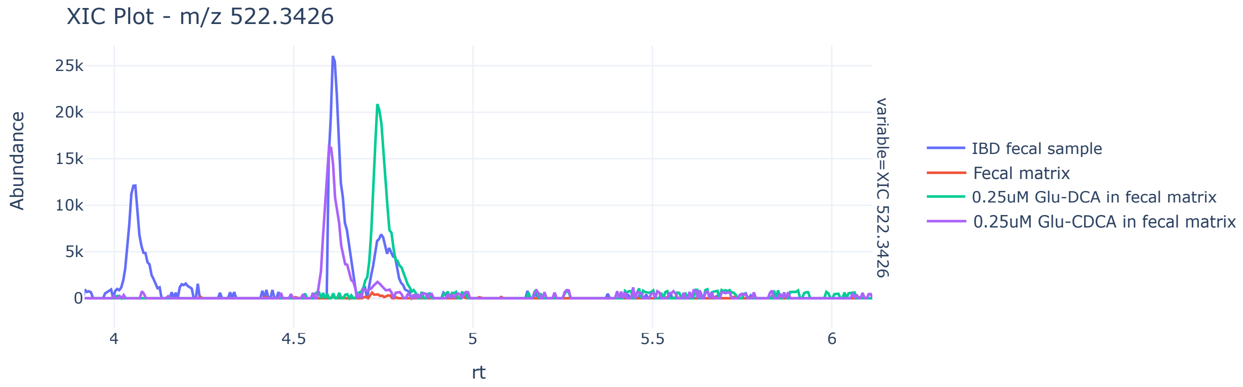

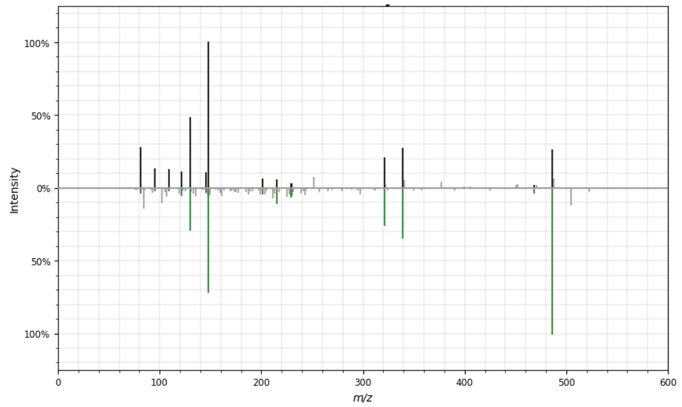

Isoleucine/Leucine conjugated cholic acid (Ile/Leu-CA)

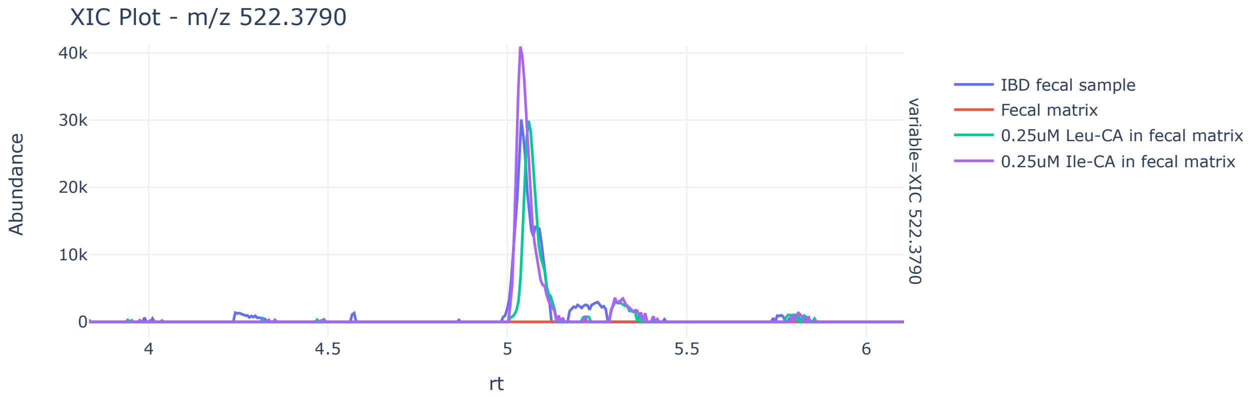

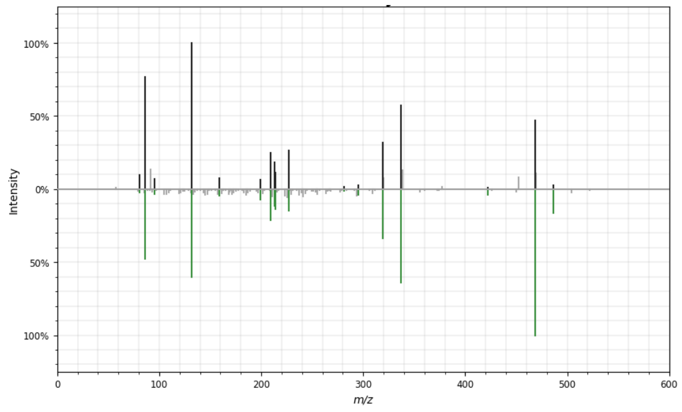

Methionine conjugated chenodeoxycholic acid (Met-CDCA)

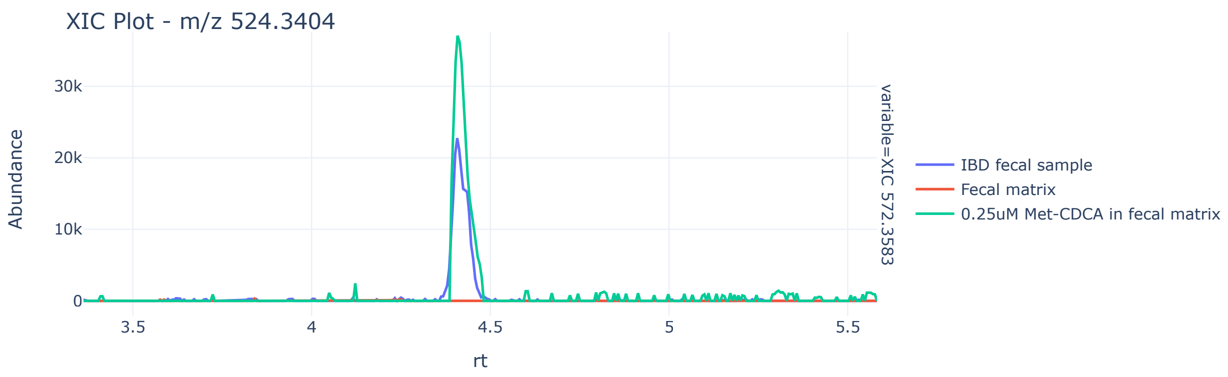

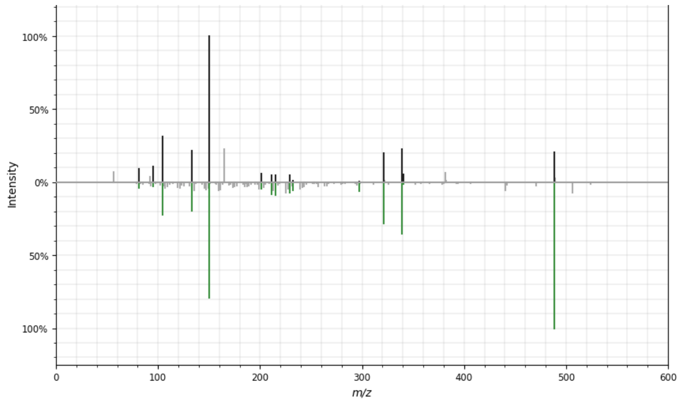

Methionine conjugated deoxycholic acid (Met-DCA)

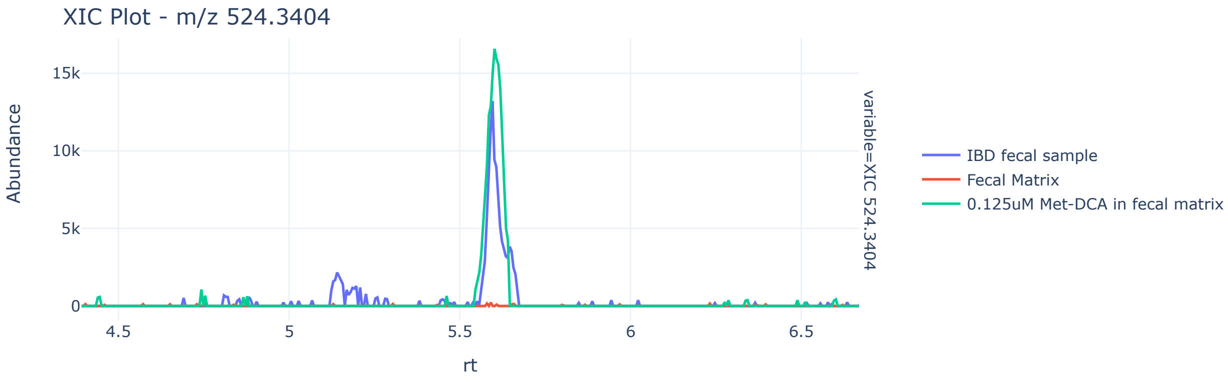

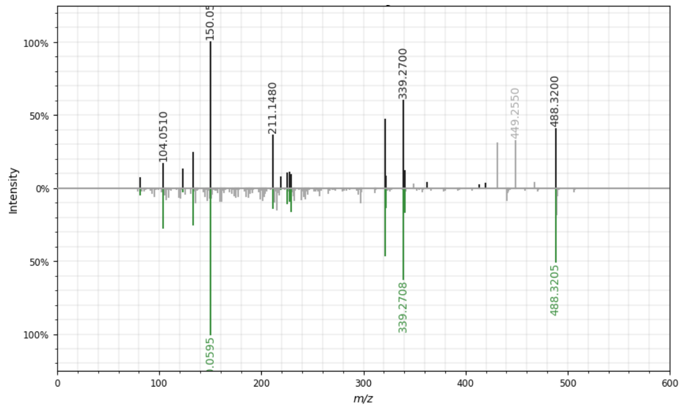

Phenylalanine conjugated cholic acid (Phe-CA)

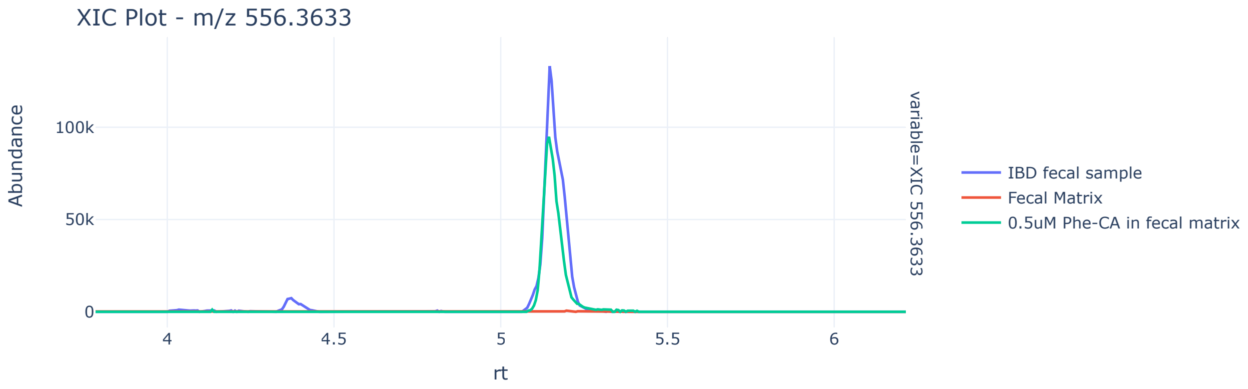

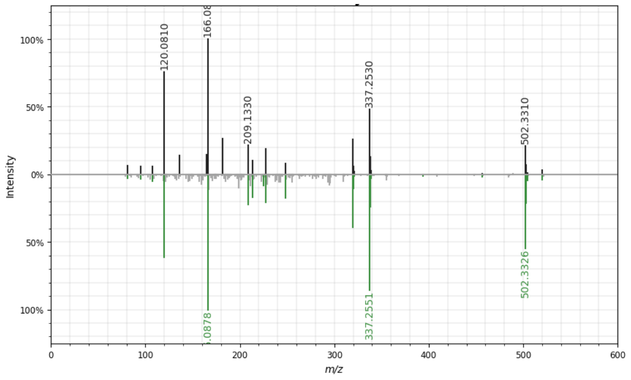

Phenylalanine conjugated chenodeoxycholic acid (Phe-CDCA)

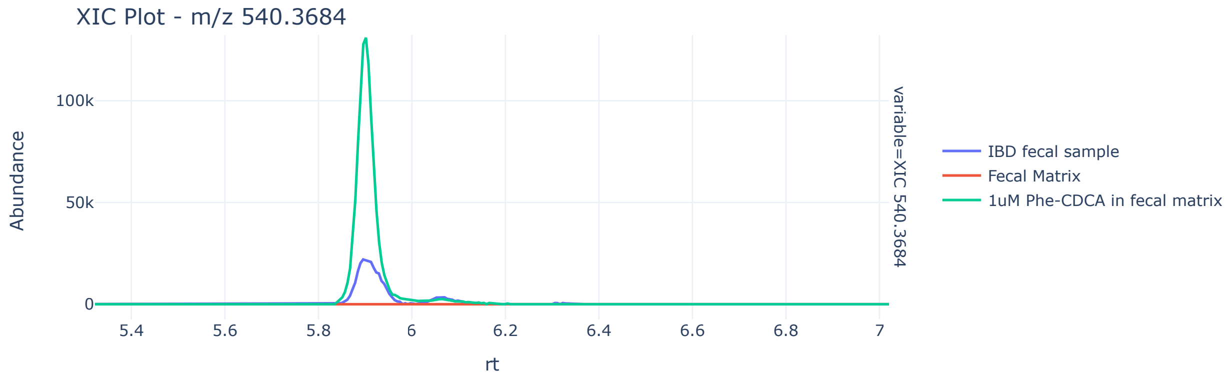

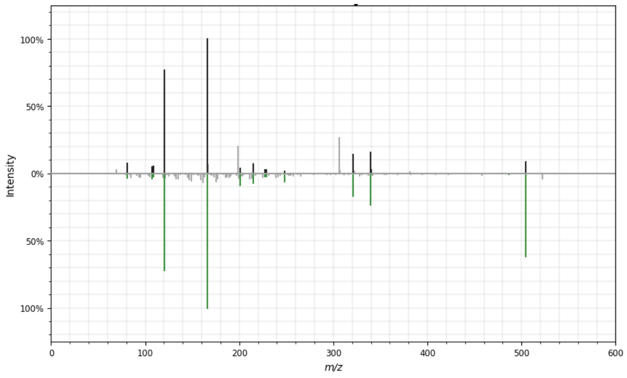

Phenylalanine conjugated deoxycholic acid (Phe-DCA)

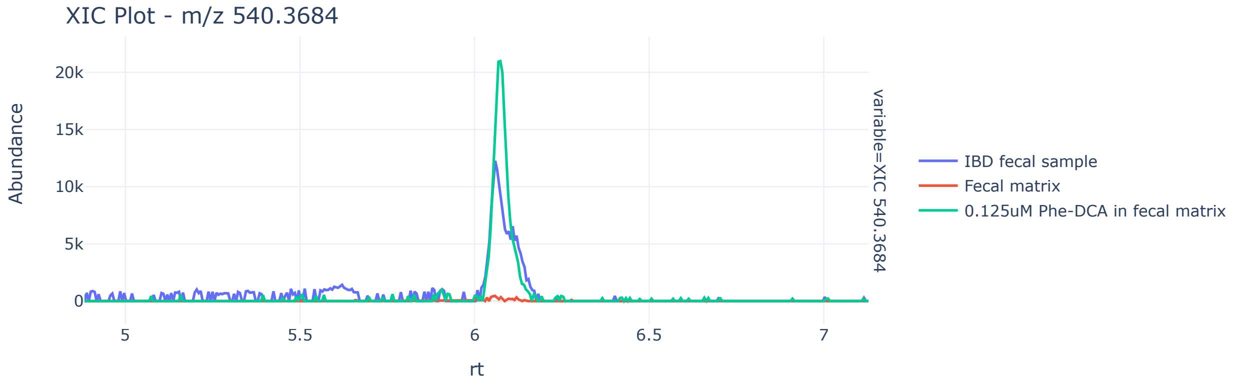

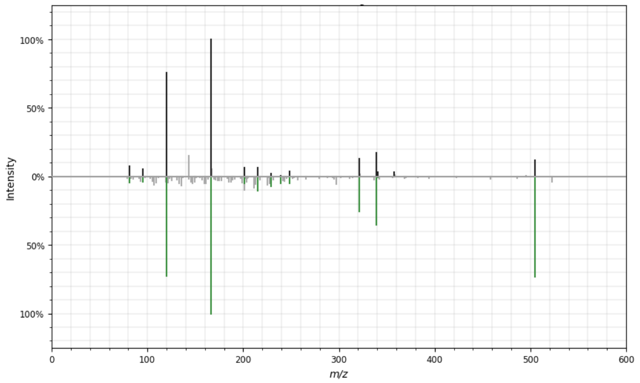

Threonine conjugated cholic acid (Thr-CA)

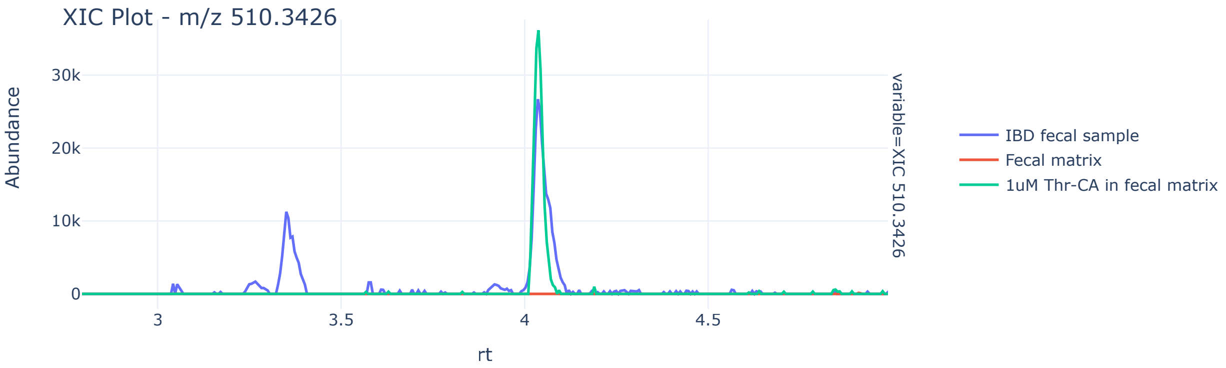

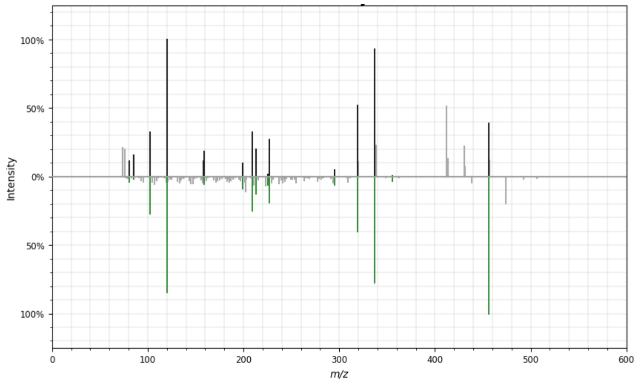

Tryptophan conjugated cholic acid (Trp-CA)

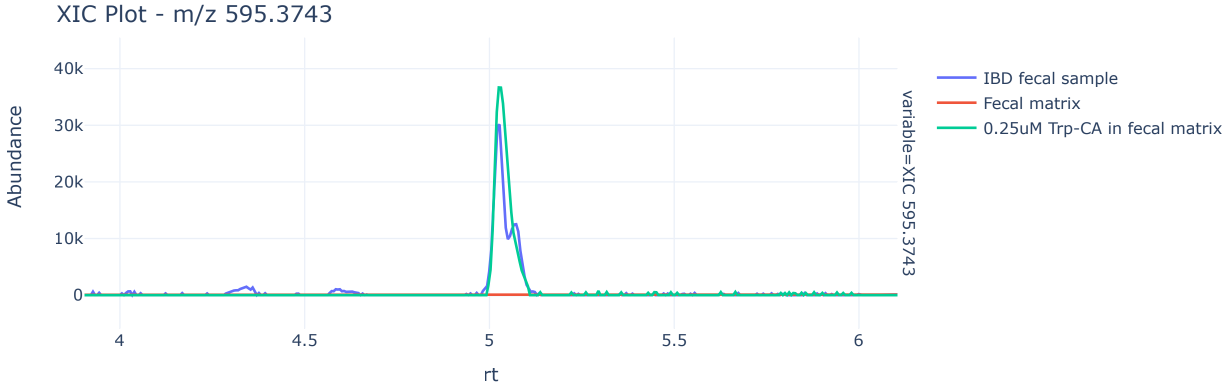

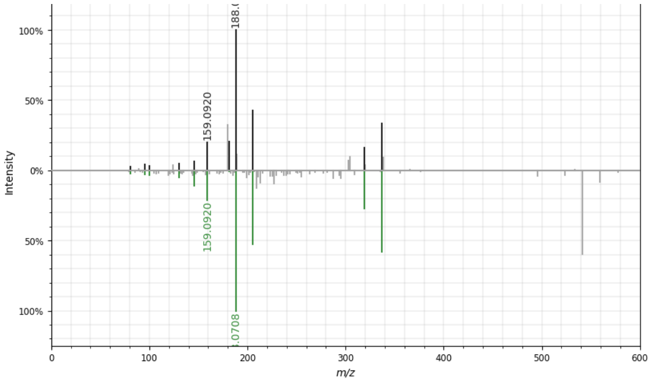

Tryptophan conjugated chenodeoxycholic acid (Trp-CDCA)

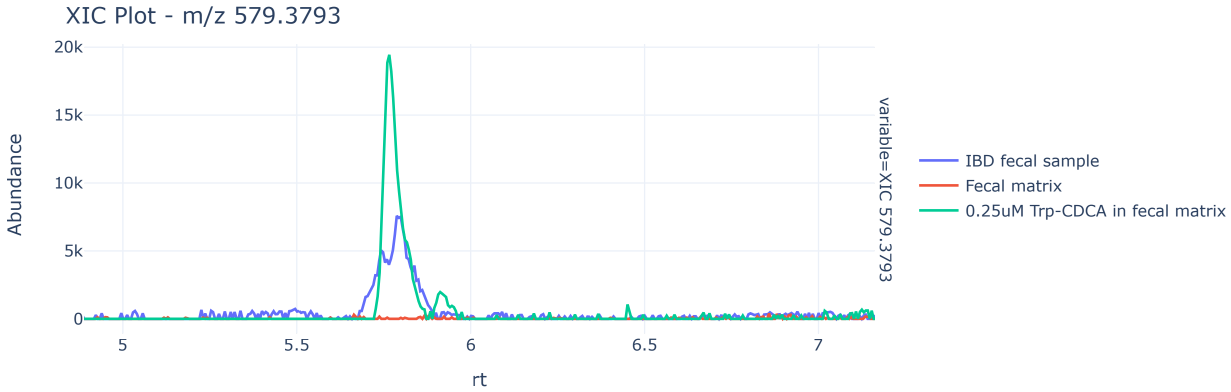

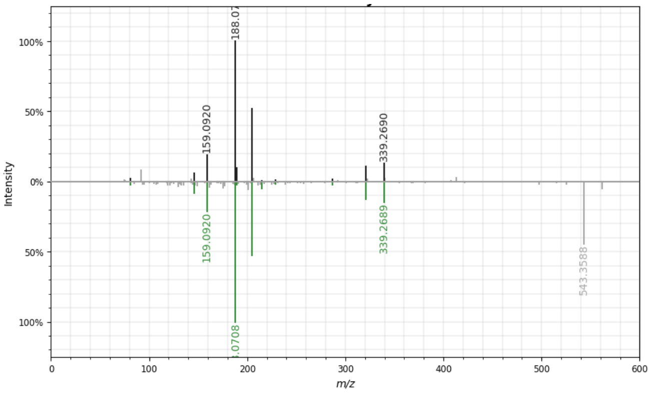

Tyrosine conjugated cholic acid (Tyr-CA)

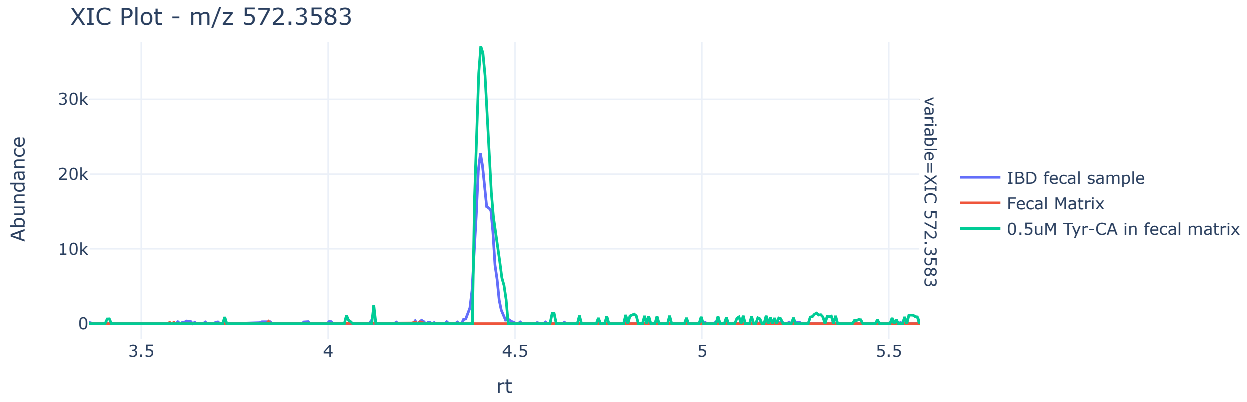

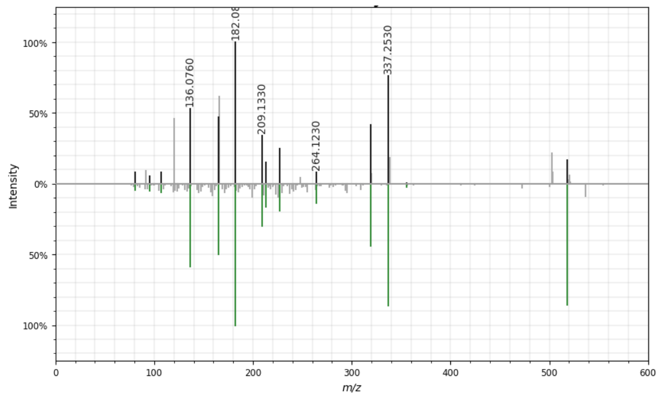

Tyrosine conjugated chenodeoxycholic acid (Tyr-CDCA)

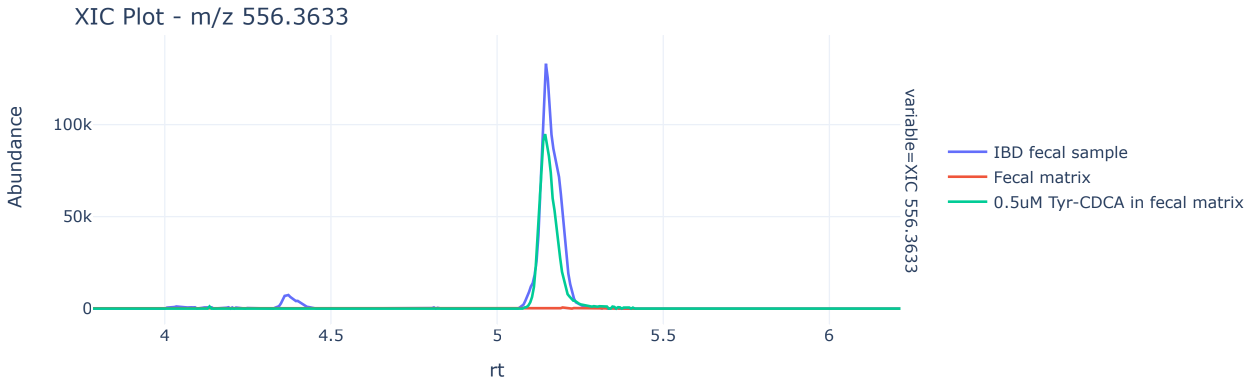

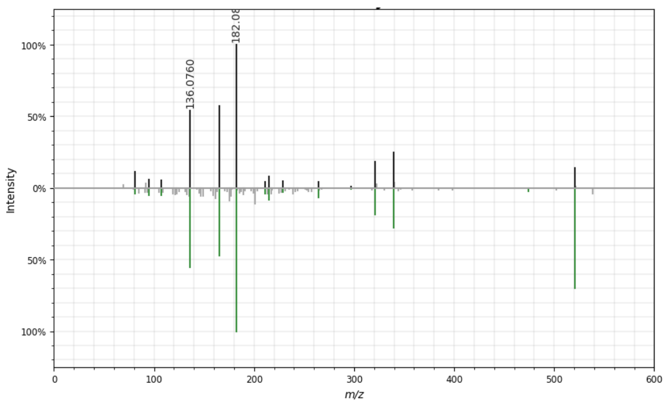

Supplement: TableS10 [file NIHMS1962612-supplement-TableS10.pdf]
