# Supplementary material for: Reverse metabolomics for the discovery of chemical structures from humans
Source: Nature. 2023 Dec 5;626(7998):419–26. doi: 10.1038/s41586-023-06906-8 (PMC10849969; doi:10.1038/s41586-023-06906-8)
Supplement: Supplementary file 1 [file 41586_2023_6906_MOESM2_ESM.pdf]

nature portfolio

Corresponding author(s): Pieter C. Dorrestein

Last updated by author(s): 10/5/2023

Reporting Summary

Nature Portfolio wishes to improve the reproducibility of the work that we publish. This form provides structure for consistency and transparency in reporting. For further information on Nature Portfolio policies, see our Editorial Policies and the Editorial Policy Checklist.

Statistics

For all statistical analyses, confirm that the following items are present in the figure legend, table legend, main text, or Methods section.

n/a	Confirmed	
☐	☒	The exact sample size (*n*) for each experimental group/condition, given as a discrete number and unit of measurement
☐	☒	A statement on whether measurements were taken from distinct samples or whether the same sample was measured repeatedly
☐	☒	The statistical test(s) used AND whether they are one- or two-sided<br>*Only common tests should be described solely by name; describe more complex techniques in the Methods section.*
☐	☒	A description of all covariates tested
☐	☒	A description of any assumptions or corrections, such as tests of normality and adjustment for multiple comparisons
☐	☒	A full description of the statistical parameters including central tendency (e.g. means) or other basic estimates (e.g. regression coefficient) AND variation (e.g. standard deviation) or associated estimates of uncertainty (e.g. confidence intervals)
☐	☒	For null hypothesis testing, the test statistic (e.g. F, t, r) with confidence intervals, effect sizes, degrees of freedom and P value noted<br>*Give P values as exact values whenever suitable.*
☒	☐	For Bayesian analysis, information on the choice of priors and Markov chain Monte Carlo settings
☒	☐	For hierarchical and complex designs, identification of the appropriate level for tests and full reporting of outcomes
☒	☐	Estimates of effect sizes (e.g. Cohen's *d*, Pearson's *r*), indicating how they were calculated

Our web collection on statistics for biologists contains articles on many of the points above.

Software and code

Policy information about availability of computer code

Data collection	Thermo XCalibur and Bruker Compass were used for LC-MS/MS data acquision. LC-IMS-MS data was acquired using Agilent MassHunter Acquisition Software. BD FACS DIVA Software (v8.0.2) were used to collect flow cytometry data.
Data analysis	Bruker DataAnalysis and GNPS Mass Spectrometry File Conversion (https://ccms-ucsd.github.io/GNPSDocumentation/fileconversion/) software were used to convert raw LC-MS/MS data to .mzXML or .mzML format. MS/MS-Chooser (https://ccms-ucsd.github.io/GNPSDocumentation/msmschooser/) and MASST (https://masst.ucsd.edu/) were used to analyze the LC-MS/MS data for the synthetic bile acid mixtures. Skyline was used for peak peaking and compound annotation of LC-IMS-MS data. FlowJo software suite (v10.8.0) was used for single-cell flow cytometry analysis. PRISM v7.0a, Excel 16.61.1, and RStudio 2022.02.3 were used to analyze data and generate graphs. No custom packages or software were developed for this analysis. Code used in this study are available at: https://github.com/emgentry/Synthesis-based-reverse-metabolomics.

For manuscripts utilizing custom algorithms or software that are central to the research but not yet described in published literature, software must be made available to editors and reviewers. We strongly encourage code deposition in a community repository (e.g. GitHub). See the Nature Portfolio guidelines for submitting code & software for further information.

Data

Policy information about availability of data

All manuscripts must include a data availability statement. This statement should provide the following information, where applicable:

- Accession codes, unique identifiers, or web links for publicly available datasets
- A description of any restrictions on data availability
- For clinical datasets or third party data, please ensure that the statement adheres to our policy

All untargeted LC-MS/MS data used in this study are publicly available at MassIVE (https://massive.ucsd.edu/) under the following accession numbers: MSV000089491 (N-acyl amide synthetic mixtures), MSV000089493 (fatty ester synthetic mixtures), MSV000087522 (conjugated bile acid synthetic mixtures acquired on Q-ToF), MSV000087523 (conjugated bile acid synthetic mixtures acquired on Orbitrap), MSV000084908 (IBD dataset used to create Figure 2e), MSV000088735 (IBD dataset used to create Figure 2f), MSV000087562 (pooled fecal samples from iHMP2 cohort - Figure 3a), MSV000092337 (IBD fecal samples used for quantitation - Figure 3b), MSV000084475 (LC-MS/MS data for bacterial culture extracts - Figure 4a and 4b), and MSV000087889 (LC-IMS-MS data for bacterial culture extracts - Figure 4c). Source data for iHMP2 and PRISM datasets are available through Metabolomics Workbench (www.metabolomicsworkbench.org) under project IDs PR000639 and PR000677, respectively. All spectra used in MASST searches are provided as a resource and can be directly downloaded from GNPS (https://gnps.ucsd.edu/ProteoSAFe/gnpslibrary.jsp?library=BILELIB19, https://gnps.ucsd.edu/ProteoSAFe/gnpslibrary.jsp?library=ECG-ACYL-AMIDES-C4-C24-LIBRARY, https://gnps.ucsd.edu/ProteoSAFe/gnpslibrary.jsp?library=ECG-ACYL-ESTERS-C4-C24-LIBRARY) and their universal spectral identifiers (USIs) and MASST job links are provided in Supplementary Table 8.

Human research participants

Policy information about studies involving human research participants and Sex and Gender in Research.

Reporting on sex and gender	This study includes information about the biological sex of patients assigned at birth, but does not include information about the gender of individuals. Here, there were an equal distribution of females (n=92) and males (n=90), as defined by sex, included in the study. Sex-based analyses are not included in this manuscript as there were no significant findings with relation to the abundance of these bile acids in different sexes.
Population characteristics	In this study, diagnosis and symptom activity were population characteristics used for the analysis. Clinical diagnosis was defined as either Crohn's disease or ulcerative colitis, based on histological and clinical features of inflammatory bowel disease. Symptom activity was self-reported as either yes or no by patients. Patients were 18-79 years old with an average age of 45.
Recruitment	Patients aged 18 years and older with a confirmed diagnosis of IBD were recruited during routine visits with no bias toward any particular group. Informed consent was obtained.
Ethics oversight	Human fecal samples were collected under UCSD IRB approval #131487.

Note that full information on the approval of the study protocol must also be provided in the manuscript.

Field-specific reporting

Please select the one below that is the best fit for your research. If you are not sure, read the appropriate sections before making your selection.

☒ Life sciences ☐ Behavioural & social sciences ☐ Ecological, evolutionary & environmental sciences

For a reference copy of the document with all sections, see nature.com/documents/nr-reporting-summary-flat.pdf

Life sciences study design

All studies must disclose on these points even when the disclosure is negative.

Sample size	For repository-scale analysis, we did not use statistical methods to determine sample size, but rather the sample size was selected by the number of files publicly available in the GNPS database and ReDU. We found an association of some conjugated bile acids with IBD which was validated in independent clinical cohorts. Therefore, the study was sufficiently powered to produce useful results. Sample size for iHMP2 (https://pubmed.ncbi.nlm.nih.gov/31142855/) and PRISM (https://www.ncbi.nlm.nih.gov/pmc/articles/PMC6342642/) cohorts were not pre-determined, but rationale for their respective sample sizes were included in reporting summaries of their associated publications. To quantify conjugated bile acids in fecal samples from IBD patients and healthy controls, 162 (72 Crohn's patients and 90 UC patients) and 15 subjects were included, respectively. For PXR and immunological assays, at least three biological replicates were used to determine fold induction of experimental samples vs. untreated control. For bacterial cultures, biological duplicates of individual strains were deemed sufficient to characterize bile acid conjugating abilities on the genus and class level.<br>For public datasets used in this manuscript,<br>MSV000084908 IBD 200 study: 203<br>MSV000088735 Pediatric IBD study: 27

Data exclusions	No data were excluded from the manuscript, but some figure panels include representative examples (e.g. conjugated bile acids elevated in IBD) rather than all of the data.
Replication	Reproducibility was verified by performing independent biological replicates and attempts at replication were successful. For PXR reporter assays and organoid experiments, each concentration of each bile acid was performed four times. For T cell differentiation assay, each experiment was performed at least three times. For culturing bacterial isolates, experiments were performed in duplicate.
Randomization	Not applicable to human IBD cohorts as data was from a cross-sectional study in which multiple pre-defined cohorts were determined by diagnosis prior to the study. For all LCMS analyses, the order in which samples were placed in 96-well plates was randomized. The order in which samples were run was also randomized per 96-well plate with the exception of the bacterial cultures. In this case, blanks were run after every six sample injections and QCs were checked to ensure no carry over or instrument drift occurred during analysis.
Blinding	For human IBD cohorts, blinding was not possible because study subjects were diagnosed prior to this study. For all other experiments, researchers were not blinded, but data was analyzed in an objective fashion.

Reporting for specific materials, systems and methods

We require information from authors about some types of materials, experimental systems and methods used in many studies. Here, indicate whether each material, system or method listed is relevant to your study. If you are not sure if a list item applies to your research, read the appropriate section before selecting a response.

Materials & experimental systems

n/a	Involved in the study	
☐	☒	Antibodies
☐	☒	Eukaryotic cell lines
☒	☐	Palaeontology and archaeology
☒	☐	Animals and other organisms
☒	☐	Clinical data
☒	☐	Dual use research of concern

Methods

n/a	Involved in the study	
☒	☐	ChIP-seq
☐	☒	Flow cytometry
☒	☐	MRI-based neuroimaging

Antibodies

Antibodies used

Rat anti-CD4 [RM4-5] APC-ef780 (Invitrogen #47-0042-82) (1:400 for flow cytometry)
Mouse anti-CD2 [RPA-2.10] PE (Invitrogen #12-0029-41) (1:400 for flow cytometry)
Rat anti-IL-17A [eBio17B7] PEcy7 (Invitrogen #25-7177-80) (1:200 for flow cytometry)
Rat anti-IFNg [XMG1.2] BV785 (Biolegend #505838) (1:400 for flow cytometry)
Rat anti-RORgt [AFKJS-9] APC (Invitrogen #17-6988-82) (1:200 for flow cytometry)
Rat anti-T-bet [4B102] PE/Dazzle 594 (Biolegend #644828) (1:200 for flow cytometry)
Live/Dead fixable aqua dead cell stain kit (Invitrogen #L34966) (1:1000 for flow cytometry)
anti-mouse CD3 [17A2] (Invitrogen # 14-0032-82) (1:500 for flow cytometry)

Validation

Rat anti-CD4 [RM4-5] APC-ef780 (Invitrogen #47-0042-82) (1:400 for flow cytometry)
-https://www.thermofisher.com/antibody/product/CD4-Antibody-clone-RM4-5-Monoclonal/47-0042-82

Mouse anti-CD2 [RPA-2.10] PE (Invitrogen #12-0029-41) (1:400 for flow cytometry)
-https://www.thermofisher.com/antibody/product/CD2-Antibody-clone-RPA-2-10-Monoclonal/12-0029-41

Rat anti-IL-17A [eBio17B7] PEcy7 (Invitrogen #25-7177-80) (1:200 for flow cytometry)
-https://www.thermofisher.com/antibody/product/IL-17A-Antibody-clone-eBio17B7-Monoclonal/25-7177-80

Rat anti-IFNg [XMG1.2] BV785 (Biolegend #505838) (1:400 for flow cytometry)
-https://www.biolegend.com/en-us/products/brilliant-violet-785-anti-mouse-ifn-gamma-antibody-7987?GroupID=GROUP24

Rat anti-RORgt [AFKJS-9] APC (Invitrogen #17-6988-82) (1:200 for flow cytometry)
-https://www.thermofisher.com/antibody/product/ROR-gamma-t-Antibody-clone-AFKJS-9-Monoclonal/17-6988-82

Rat anti-T-bet [4B102] PE/Dazzle 594 (Biolegend #644828) (1:200 for flow cytometry)
-https://www.biolegend.com/en-us/products/pe-dazzle-594-anti-t-bet-antibody-11891

Live/Dead fixable aqua dead cell stain kit (Invitrogen #L34966) (1:1000 for flow cytometry)
-https://www.thermofisher.com/order/catalog/product/L34965

anti-mouse CD3 [17A2] (Invitrogen # 14-0032-82) (1:500 for flow cytometry)
https://www.thermofisher.com/antibody/product/CD3-Antibody-clone-17A2-Monoclonal/14-0032-82

Eukaryotic cell lines

Policy information about cell lines and Sex and Gender in Research

Cell line source(s)	Bone marrow-derived dendritic cells were derived from WT C57BL6/J mice and CD4+ T cells were derived from Foxp3-hCD2 reporter mice. Huh7 cells were obtained from Xenotech. Organoids were derived from the small intestine of PXR-humanized mice in C57Bl6/N background.
Authentication	Cell lines used were not authenticated.
Mycoplasma contamination	Cell lines were not tested for mycoplasma contamination.
Commonly misidentified lines (See ICLAC register)	No commonly misidentified lines were used.

Flow Cytometry

Plots

Confirm that:

☒ The axis labels state the marker and fluorochrome used (e.g. CD4-FITC).

☒ The axis scales are clearly visible. Include numbers along axes only for bottom left plot of group (a 'group' is an analysis of identical markers).

☒ All plots are contour plots with outliers or pseudocolor plots.

☒ A numerical value for number of cells or percentage (with statistics) is provided.

Methodology

Sample preparation	Naïve CD4+ T cells were isolated from Foxp3-hCD2 reporter mice and cultured with bone marrow derived dendritic cells from WT C57BL6/J mice under Th17 polarizing conditions. Cells were treated with 100 µM of bile acids on day 0 and collected on day 3 for flow cytometric analysis. Cells were first stained with Live/Dead dye and surface markers and then fix/permed with eBioscience™ Foxp3 / Transcription Factor Staining Buffer Set. RORgt were stained for overnight and other intracellular markers and cytokines were stained for 3h.
Instrument	BD LSR II
Software	BD FACS DIVA Software (v8.0.2) were used to collect data. FlowJo (v10.8.0) software were used to analyze data. No custom code has been deposited into a community repository.
Cell population abundance	The purity of Th17 cells were determined by the labeling of specific markers. Th17 cells were defined as CD4+ hCD2 (Foxp3)- Tbet- RORgt+ cells. The abundance of Th17 cells were 46.1 ± 6.83%.
Gating strategy	Samples were first gated to distinguish population of cells based on the side and forward scatter properties (SSC-A vs FSC-A), followed by singlets (FSC-H vs FSC-A and SSC-H vs SSC-A). Live cells were identified by using live/dead staining. Th17 cells were defined as CD4+ hCD2 (Foxp3)- Tbet- RORgt+ cells. IL-17A and IFNg expression were examined on Th17 populations.

☒ Tick this box to confirm that a figure exemplifying the gating strategy is provided in the Supplementary Information.
